# A General Synthesis of Cross-Conjugated Enynones through Pd Catalyzed Sonogashira Coupling with Triazine Esters

**DOI:** 10.3390/molecules28114364

**Published:** 2023-05-26

**Authors:** Dezhi Lin, Yunfang Liu, Hongyu Yang, Xiao Zhang, Huaming Sun, Yajun Jian, Weiqiang Zhang, Jianming Yang, Ziwei Gao

**Affiliations:** 1 Key Laboratory of Applied Surface and Colloid Chemistry (MOE), Xi’an Key Laboratory of Organometallic Material Chemistry, School of Chemistry and Chemical Engineering, Shaanxi Normal University, Xi’an 710119, China; 2 South China Institute of Environmental Science, Ministry of Ecology and Environment, Guangzhou 510655, China; 3 Xi’an Modern Chemistry Research Institute, Xi’an 710065, China

**Keywords:** palladium catalysis, cross-coupling, C-O activation, ketone synthesis

## Abstract

The palladium-catalyzed Sonogashira coupling of α, β-unsaturated acid derivatives offers a diversity-oriented synthetic strategy for cross-conjugated enynones. However, the susceptibility of the unsaturated C-C bonds adjacent to the carbonyl group toward Pd catalysts makes the direct conversion of α, β-unsaturated derivatives as acyl electrophiles to cross-conjugated ketones rare. This work presents a highly selective C-O activation approach to prepare cross-conjugated enynones using α, β-unsaturated triazine esters as acyl electrophiles. Under base and phosphine ligand-free conditions, NHC-Pd(II)-Allyl precatalyst alone catalyzed the cross-coupling of α, β-unsaturated triazine esters with terminal alkynes efficiently, yielding 31 cross-conjugated enynones with diverse functional groups. This method demonstrates the potential of triazine-mediated C-O activation for preparing highly functionalized ketones.

## 1. Introduction

Cross-conjugated enynones, or 1-en-4-yn-3-ones, play a crucial role in organic chemistry due to their unique reaction centers. These enynones consist of carbon–carbon double and triple bonds linked with a carbonyl group, which makes them valuable building blocks for synthesizing complex heterocycles and carbocycles [1,2,3,4]. The resulting compounds can contain oxygen, nitrogen, and sulfur and are used in pharmaceuticals and materials science, among other fields [5,6,7]. It is worth noting that the cross-conjugated enynone structure is not exclusive to synthetic compounds; it can also be found in nature in various plants, fungi, and sea organisms [8]. Researchers have even found Petroacetylene, a compound containing two enynone units, in the sea sponge *Petrosia solida* [9]. Broad application and occurrence highlight the importance of preparing these compounds’ synthetic potential and their role in nature.

Since there is limited access to cross-conjugated enynones from natural resources, considerable effort has been put into preparing conjugated acetylenic carbonyl compounds through various synthetic methods from commercially available starting materials. These methods include oxidatively dimerizing phenylacetylene [10], oxidizing secondary vinyl ethynyl alcohols [11,12,13,14,15], and aldol crotonic condensation of ethynyl methyl ketones with aromatic and heteroaromatic aldehydes [16]. Alternatively, transition metal-catalyzed coupling reactions offer a gentler approach for preparing acetylenic carbonyl derivatives avoiding the oxidation step. One such reaction is the palladium-catalyzed cross-coupling of terminal alkynes and metallated organic halide derivatives in the presence of carbon monoxide [17]. Copper(I) iodide in triethylamine catalyzed two components cross-coupling of cinnamoyl chloride and phenylacetylenes; however, the yield of 1-en-4-yn-3-one is lower in this case than in Pd-catalyzed reactions [18].

Pd-catalyzed cross-coupling reactions using carboxylic acid derivatives have been widely used in ketone synthesis since Milstein and Stille demonstrated the synthetic utility of acid chlorides as coupling partners decades ago [19]. Owing to the availability and structural diversity of carboxylic acids, various coupling partners of acyl halides [20,21,22,23,24], amides [25], thioesters [26], and esters [27], have been applied as acyl electrophiles in Pd-catalyzed cross-coupling reactions for drug discovery, natural product synthesis, and polymer synthesis [28]. In this context, Sonogashira cross-coupling of carboxylic acids derivatives represents a central strategy for 1-yn-3-ones’ synthesis. [29]. Despite aromatic and alkyl carboxylic acids derivatives’ success in 1,3-ynone synthesis, α, β-unsaturated carboxylic acid derivatives are rarely investigated as acyl electrophiles for the preparation of 1-en-4-yn-3-ones. Although few examples of palladium-catalyzed cross-coupling of cinnamic chlorides with terminal acetylenes provide a carbon monoxide-free protocol for preparing enynones, the catalyst systems were employed to selectively cleavage C(acyl)-O in the presence of C=C bond near the carbonyl group in acyl electrophile. The catalyst systems used for this reaction include Pd(PPh_3_)_4_ with ZnCl_2_ or ZnBr_2_ [30], Pd/BaSO_4_ [31], Pd/C [32], Pd(OAc)_2_ [33], Pd(PPh_3_)_2_Cl/CuI [34], and KF/Al_2_O_3_/Pd(PPh_3_)_2_Cl_2_/CuI with microwave activation [35], palladium nanoparticles embedded into the poly-1,4-phenylene sulfide polymer matrix [36], and even-more-complicated palladium-based catalyst systems [37,38]. To circumvent the complicated catalyst system, an α, β-unsaturated acyl electrophile with well-balanced stability and C(acyl)-O activity is highly demanding for the Pd-catalyzed preparation of cross-conjugated enynone.

To address this issue, we proposed that triazine esters are effective acylating reagents for Pd-catalyzed C-C coupling. Unlike traditional esters, the triazine ring is strongly electron-withdrawing to activate the C(acyl)-O bond. Meanwhile, the coordination ability of the triazine ring is advantageous for palladium-catalyzed carboxylate coupling reactions. Our group used acyl acid triazine esters as acylating reagents in Pd-catalyzed cross-coupling of C(acyl)-C(sp) bonds [27]. Using triazine as a core, we designed multifunctional phosphine ligands for Pd-catalyzed carbonylative reaction [39]. Recently, triazines as wingtips of NHC ligands accelerated the transmetallation of Pd-catalyzed carbonylative Sonogashira reactions [40]. Considering triazine’s unique ability to activate C(acyl)-O bonds and its catalytic implications, we envisioned triazine esters of α, β-unsaturated carboxylic acids as potent acyl electrophiles for Pd catalysis. Herein, we report Pd-catalyzed acyl Sonogashira reaction with bench-stable α, β-unsaturated carboxylic acids triazine esters as acyl electrophiles, by which cross-conjugated enynones were synthesized in a mild and selective manner.

## 2. Results and Discussion

We utilized a Pd-catalyzed Sonogashira reaction to synthesize cross-conjugated enynones from α, β-unsaturated carboxylic acid triazine esters as acylating reagents and alkynes. The triazine esters were prepared using Kamiński’s method in high yields from the α, β-unsaturated carboxylic acid using toluene as the solvent. The cross-coupling of cinnamic acid triazine ester **1a** with phenylacetylene **3a** was chosen to optimize palladium catalysts and solvents. Pd(OAc)_2_ alone catalyzed the reaction without ligand or base, yielding the desired enynone **3aa** in various organic solvents (Table 1, Entries **1**–**5**). The most suitable solvent for the reaction was MeCN, which yielded a 42% yield (Entry **6**). The cinnamic acid anhydrous was detected in the raw product, which might be formed by the degradation of unconverted triazine esters due to the low activity of Pd(OAc)_2_. We screened palladium catalysts for the Sonogashira reactions and found that Pd(OAc)_2_ gave a promising 42% yield in MeCN (Entry **6**), while PdCl_2_ and PdCl_2_(PPh)_2_ failed to catalyze the coupling reaction (Entries **7**–**8**).

It is worth noting that allylic Pd(II) precatalysts, such as [Pd(cinnamyl)Cl]_2_, [(2-Methylallyl)PdCl]_2_, and [(allyl)PdCl]_2_ (Entries **9**–**11**), substantially enhanced the activity of Pd, resulting in yields of 62–58%, respectively. NHC ligands further improved the allylic Pd(II) precatalysts Pd-1 and Pd-2, which yielded 75% and 78%, respectively (Entry **13**). Remarkably, Pd-3, containing a less bulky NHC ligand, catalyzed the coupling reaction with 95% yields (Entry **14**). The Pd-loading of Pd-3 as a precatalyst could be reduced to 1 mol% (Entry **16**) without significantly impacting the reaction outcome. During optimization, low-yielding reactions generally showed the degradation of triazine ester into the corresponding anhydrous and no evidence of C(acyl)-O cleavage or decarbonylation pathways was observed.

**Scheme 1:**
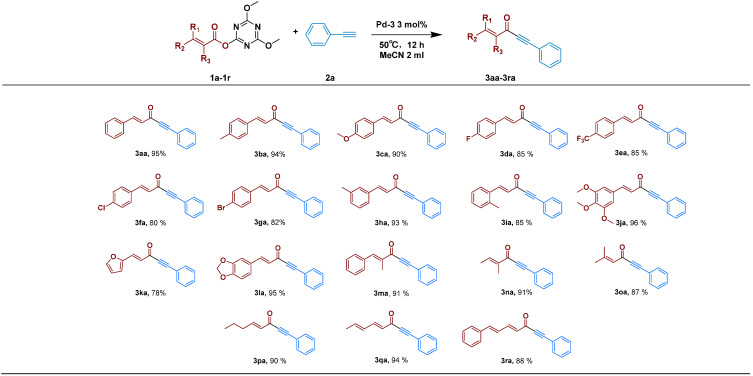
Scope and limitation of α, β- triazine esters in of Pd catalyzed Sonogashira coupling reaction. All reactions were performed with 0.25 mmol of triazine esters (**1a**–**1r**) and 0.5 mmol of terminal alkynes **2a**. Yields are of isolated products after purification by flash chromatography on silica gel.

Under mild optimal conditions, we evaluated the potential of triazine esters as acyl electrophiles in Pd-catalyzed Sonogashira coupling reactions. Without adding a base and phosphine ligand, Pd(II) efficiently catalyzed the coupling reactions at 50 °C in MeCN. As shown in Scheme 1, a broad range of triazine esters are compatible, converting into enynones with generally high to excellent yields. We began by exploring the coupling of cinnamic triazine esters with phenylacetylene **2a**. Our findings revealed that several substitution patterns and types of cinnamic triazine esters **1a**–**1k** provided satisfactory yields. For instance, both electron-rich and electron-poor cinnamic triazine esters, including those with *para*- (**1b, 1c**), *meta*- (**1h**), and sterically hindered *ortho*- (**1i**) substitution patterns, were effective in producing the desired enynone products. Additionally, the coupling reaction using 3,4,5-methoxyphenyl (**1j**), catecholyl (**1k**), and furanyl (**1l**) cinnamic triazine testers gave 96%, 78%, and 95% yields, respectively.

We have expanded the protocol to include acrylic triazine esters **1l–1r**, which are significant in synthetic chemistry. It is important to note that acrylic triazine esters with methyl or *n*-propyl at both *α*- and *β*- positions can be coupled with phenylacetylene and converted to the corresponding unsaturated enynones (**3ma**–**3oa**) with high yields of 87–91%.

Sorbic triazine esters, which are challenging substrates, can be selectively activated at C(acyl)-O in the presence of the diene close to carbonyl groups to produce two α, β, γ, *δ*-dienynones (**3qa**, **3ra**) with high yields of 94% and 88%, respectively. These extended conjugated systems would be difficult to be constructed using conventional nucleophilic addition methodologies, highlighting the practicality of these α, β-unsaturated carboxylic acid esters as acyl electrophiles.

**Scheme 2:**
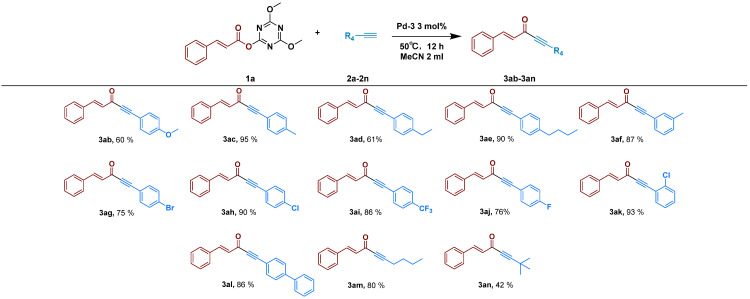
Scope and limitation of terminal alkynes of Pd catalyzed Sonogashira coupling reaction. All reactions were performed with 0.25 mmol of cinnamic triazine ester (**1a**) and 0.5 mmol of terminal alkynes (**2b**–**2n**). Yields are of isolated products after purification by flash chromatography on silica gel.

Various functionalized alkynes reacted efficiently with cinnamic triazine ester (**1a**) to produce the desired cross-conjugated enynones in high yields (Scheme 2). Aryl alkynes with electron-donating groups at para- and meta-positions (**2b**–**2f**) and electron-withdrawing groups (**2j**–**2k**) provided satisfactory yields ranging from 60% to 95%. Sterically bulky ortho-chlorine in **2k** was also fully compatible with Pd-catalyzed cross-couplings of cinnamic triazine (**1a**), producing the corresponding enynones with 93% yields. Enynones bearing biphenyl (**3al**) were isolated in 86% yields. Aliphatic alkynes (**2m**) also acted as good nucleophiles coupled with the triazine ester. On the other hand, alkynes with sterically hindered groups, such as *i*- butyl (**3an**), produced the corresponding enynones 3am with a 42% yield. These results highlight the advantages of using triazine ester as acyl electrophiles for synthesizing cross-conjugated enynones with broad applicability.

## 3. Experimental

### 3.1. General Information

All reactions were performed using a 27.5 × 72.5 mm screw-caped vial under air without N_2_ protection. Glassware was dried in an oven before use. All reactions were stirred with magnetic followers. All stated temperatures refer to external bath/heating aluminum block temperatures. Reagents were purchased from commercial sources and were used as received unless mentioned otherwise. Reactions were monitored by thin-layer chromatography using silica gel. The thin-layer chromatography (TLC) employed glass 0.25 mm silica-gel plates. Flash chromatography columns were packed with 200–300 mesh silica-gel in petroleum (the boiling point was between 60–90 °C). Gradient flash chromatography was conducted eluting with a continuous gradient from petroleum to the indicated solvent, and they were listed as volume/volume ratios. ^1^H NMR and ^13^C NMR were recorded on a Bruker-400 MHz Spectrometer (^1^H: 400 MHz, ^13^C: 101 MHz), using Acetonitrile-d_3_ as the solvent at room temperature. The chemical shifts (δ) were expressed in ppm, and the coupling constants (J) were expressed in Hz. High-resolution mass spectra (HRMS) were recorded on a Bruker MAXIS spectrometer.

### 3.2. General Method for the Synthesis of α, β-Unsaturated Triazine Esters

Under an air atmosphere, a round-bottom flask of 150 mL equipped with a magnetic stir bar was charged successively with α-β unsaturated carboxylic acid (**1sa**–**1sr** 1.0 mmol), NMM (1.2 mmol) and 10 mL of toluene. The reaction mixture was stirred at room temperature to completely dissolve. Then, 2-Chloro-4,6-dimethoxy-1,3,5-triazine (CDMT) (1.2 mmol) was dissolved in 10 mL toluene into the reaction mixture dropwise. After the reaction, the resulting mixture was diluted with 20.0 mL of ethyl acetate and filtrated. The residue was washed with 1N citric acid solution, H_2_O, and 1N sodium bicarbonate, respectively. The organic layer was dried over anhydrous MgSO_4_ and concentrated under reduced pressure. Purification of the crude product by flash chromatography on silica gel using the mixed solvent system of petroleum ether (PE) and ethyl acetate (EA) afforded the desired products (**1a**–**1r**).

### 3.3. General Procedure for the Coupling of α, β-Unsaturated Triazine Esters and Alkyne

The general procedure for the optimization reactions: an oven-dried 30-mL screw-cap vial equipped with a magnetic stirring bar was charged with the corresponding α, β-unsaturated triazine esters (0.25 mmol, 1.0 equiv.), alkyne (0.5 mmol, 2 equiv.), Pd-3 (3.0 mol%). Then, MeCN (2.0 mL) was added. The reaction mixture was stirred at 50 °C. The conversion was monitored by TLC analysis, and unless otherwise noted, the triazine esters were fully converted within 12 h. The reaction mixture was diluted with 20.0 mL of ethyl acetate, filtrated, and concentrated under reduced pressure. Purification of the crude product by flash chromatography on silica gel using the mixed-solvent system of petroleum ether (PE) and ethyl acetate (EA) afforded the desired products.

(*E*)-1,5-diphenylpent-1-en-4-yn-3-one (**3aa**) [12], the general method using Triazine Ester **1a** and Phenylacetylene **2a** gave the title compound **3aa** in 95% yield; ^1^H NMR (400 MHz, Acetonitrile-d_3_): δ/ppm = 8.02 (d, J = 16.2 Hz, 1H), 7.73 (d, J = 7.3 Hz, 5H), 7.58–7.45 (m, 7H), 6.92 (d, J = 16.2 Hz, 1H). ^13^C NMR (101 MHz, Acetonitrile-d_3_): δ/ppm = 177.94, 148.62, 134.26, 133.04, 131.32, 131.02, 129.17, 128.97, 128.93, 128.55, 119.93, 90.75, 86.05.

(*E*)-5-phenyl-1-(p-tolyl)pent-1-en-4-yn-3-one (**3ba**) [41], the general method using Triazine Ester **1b** and Phenylacetylene **2a** gave the title compound **3ba** in 94% yield; ^1^H NMR (400 MHz, Acetonitrile-d_3_): δ/ppm = 7.97 (d, J = 16.1 Hz, 1H), 7.74–7.69 (m, 2H), 7.61 (d, J = 8.1 Hz, 2H), 7.55–7.43 (m, 3H), 7.27 (d, J = 8.0 Hz, 2H), 6.85 (d, J = 16.1 Hz), 2.37 (s, 3H). ^13^C NMR (400 MHz, Acetonitrile-d_3_): δ/ppm = 177.93, 148.77, 142.19, 133.00, 131.49, 130.94, 129.81, 128.98, 127.60, 119.98, 90.56, 86.10, 20.69.

(*E*)-1-(4-methoxyphenyl)-5-phenylpent-1-en-4-yn-3-one (**3ca**) [41], the general method using Triazine Ester **1c** and Phenylacetylene **2a** gave the title compound **3ca** in 90% yield; ^1^H NMR (400 MHz, Acetonitrile-d_3_): δ/ppm = 7.97 (d, J = 16.1 Hz, 1H), 7.75–7.65 (m, 4H), 7.58–7.51 (m, 1H), 7.48 (m, 2H), 7.04–6.97 (m, 2H), 6.79 (d, J = 16.1 Hz, 1H), 3.84(s, 3H). ^13^C NMR (400 MHz, Acetonitrile-d_3_): δ/ppm = 177.83, 162.43, 148.66, 132.96, 130.90, 128.94, 126.79, 126.33, 120.07, 114.61, 90.28, 86.13, 55.32.

(*E*)-1-(4-fluorophenyl)-5-phenylpent-1-en-4-yn-3-one (**3da**) [41], the general method using Triazine Ester **1d** and Phenylacetylene **2a** gave the title compound **3da** in 85% yield; ^1^H NMR (400 MHz, Acetonitrile-d_3_): δ/ppm = 7.97 (d, J = 16.0 Hz, 1H), 7.79–7.69 (m, 4H), 7.58–7.44 (m, 3H), 7.24–7.15 (m, 2H), 6.84 (d, J = 16.1 Hz, 1H). ^13^C NMR (400 MHz, Acetonitrile-d_3_): δ/ppm = 177.80, 164.39 (d, J = 251.6 Hz), 147.26, 133.04, 131.22 (d, J = 8.8 Hz), 131.02, 130.76 (d, J = 12.6 Hz), 128.96, 128.38, 119.89, 116.14 (d, J = 22.3 Hz), 90.77, 85.99.

(*E*)-5-phenyl-1-(4-(trifluoromethyl)phenyl)pent-1-en-4-yn-3-one (**3ea**) [42], the general method using Triazine Ester **1e** and Phenylacetylene **2a** gave the title compound **3ea** in 85% yield; ^1^H NMR (400 MHz, CDCl_3_): δ/ppm = 8.03 (d, J = 16.0 Hz, 1H), 7.88 (d, J = 8.3 Hz, 2H), 7.78–7.71 (m, 4H), 7.58–7.53 (m, 1H), 7.49 (m, 2H), 6.98 (d, J = 16.1 Hz, 1H). ^13^C NMR (400 MHz, CDCl_3_): δ/ppm = 177.71, 146.32, 138.04, 133.13, 131.16, 130.70, 129.32, 128.98, 125.93 (d, J = 17.2 Hz), 119.73, 91.30, 85.94.

(*E*)-1-(4-chlorophenyl)-5-phenylpent-1-en-4-yn-3-one (**3fa**) [41], the general method using Triazine Ester **1f** and Phenylacetylene **2a** gave the title compound **3fa** in 80% yield; ^1^H NMR (400 MHz, Acetonitrile-d_3_): δ/ppm = 7.95 (d, J = 16.1 Hz, 1H), 7.75–7.66 (m, 4H), 7.61–7.38 (m, 5H), 6.88 (d, J = 16.1 Hz, 1H). ^13^C NMR (400 MHz, Acetonitrile-d_3_): δ/ppm = 177.76, 146.98, 136.55, 133.07, 131.06, 130.39, 129.25, 129.07, 128.96, 119.84, 90.95, 85.99.

(*E*)-1-(4-bromophenyl)-5-phenylpent-1-en-4-yn-3-one (**3ga**) [43], the general method using Triazine Ester **1g** and Phenylacetylene **2a** gave the title compound **3ga** in 82% yield; 1H NMR (400 MHz, Acetonitrile-d_3_): δ/ppm = 7.95 (d, J = 16.0 Hz, 1H), 7.78–7.70 (m, 2H), 7.65–7.45 (m, 7H), 6.90 (d, J = 16.1 Hz, 1H). 13C NMR (400 MHz, Acetonitrile-d_3_): δ/ppm = 177.78, 147.08, 133.43, 133.07, 132.26, 131.07, 130.56, 129.14, 128.97, 125.01, 119.83, 90.98, 85.98.

(*E*)-5-phenyl-1-(*m*-tolyl)pent-1-en-4-yn-3-one (**3ha**), the general method using Triazine Ester **1h** and Phenylacetylene **2a** gave the title compound **3ha** in 93% yield; ^1^H NMR (400 MHz, Acetonitrile-d_3_): δ/ppm = 7.97 (d, J = 16.1 Hz, 1H), 7.80–7.65 (m, 2H), 7.63–7.41 (m, 5H), 7.41–7.19 (m, 2H), 6.89 (d, J = 16.1 Hz, 1H), 2.37(s, 3H). ^13^C NMR (400 MHz, Acetonitrile-d_3_): δ/ppm = 177.95, 148.87, 139.04, 134.19, 133.03, 132.09, 131.00, 129.47, 129.05, 128.97, 128.35, 126.15, 119.93, 86.05, 20.38. **IR(KBr)**: ν(C≡C): 2212 cm^−1^; ν(C=O): 1627 cm^−1^. **HRMS(ESI) *m*/*z*:** [M+H]^+^ calcd. for C_18_H_15_O: 247.1117; Found: 247.1114.

(*E*)-5-phenyl-1-(*o*-tolyl)pent-1-en-4-yn-3-one (**3ia**), the general method using Triazine Ester **1i** and Phenylacetylene **2a** gave the title compound **3ia** in 85% yield; ^1^H NMR (400 MHz, Acetonitrile-d_3_): δ/ppm = 8.03 (d, J = 16.1 Hz, 1H), 7.74–7.64 (m, 3H), 7.50–7.40 (m, 4H), 7.36 (d, J = 5.7 Hz, 1H), 7.28 (t, J = 5.6 Hz, 1H), 6.92 (d, J = 16.1 Hz, 1H), 2.57(s, 3H). ^13^C NMR (400 MHz, Acetonitrile-d_3_): δ/ppm = 177.99, 148.51, 142.20, 134.21, 133.53, 131.31, 131.05, 130.03, 129.17, 128.88, 128.61, 126.18, 119.78, 90.04, 89.74. **IR(KBr)**: ν(C≡C): 2212 cm^−1^; ν(C=O): 1626 cm^−1^. **HRMS(ESI) *m*/*z*:** [M+H]^+^ calcd. for C_18_H_15_O: 247.1117; Found: 247.1112.

(*E*)-5-phenyl-1-(3,4,5-trimethoxyphenyl)pent-1-en-4-yn-3-one (**3ja**), the general method using Triazine Ester **1j** and Phenylacetylene **2a** gave the title compound **3ja** in 96% yield; ^1^H NMR (400 MHz, Acetonitrile-d_3_): δ/ppm = 7.92 (d, J = 16.1 Hz, 1H), 7.75–7.71 (m, 2H), 7.58–7.45 (m, 3H), 7.01(s, 2H), 6.89 (d, J = 16.0 Hz, 1H), 3.86(s, 6H), 3.77(s, 3H). ^13^C NMR (400 MHz, Acetonitrile-d_3_): δ/ppm = 177.82, 153.67, 148.82, 140.80, 132.99, 130.94, 129.76, 128.95, 127.92, 119.97, 106.43, 90.49, 86.11, 60.07, 55.94. **IR(KBr)**: ν(C≡C): 2210 cm^−1^; ν(C=O): 1626 cm^−1^. **HRMS(ESI) *m*/*z*:** [M+H]^+^ calcd. for C_20_H_19_O_4_: 323.1277; Found: 323.1270.

(*E*)-1-(benzo[d][1,3]dioxol-5-yl)-5-phenylpent-1-en-4-yn-3-one (**3la**) [44], the general method using Triazine Ester **1k** and Phenylacetylene **2a** gave the title compound **3ka** in 91% yield; ^1^H NMR (400 MHz, Acetonitrile-d_3_): δ/ppm = 7.91 (d, J = 16.0 Hz, 1H), 7.71 (d, J = 7.2 Hz, 2H), 7.55–7.44 (m, 3H), 7.24 (d, J = 8.1 Hz, 2H), 6.91 (d, J = 7.9 Hz, 1H), 6.76 (d, J = 16.0 Hz, 1H), 6.04 (s, 2H). ^13^C NMR (400 MHz, Acetonitrile-d_3_): δ/ppm = 177.76, 150.73, 148.58, 132.97, 130.90, 128.94, 128.61, 126.72, 126.12, 120.02, 108.60, 106.76, 102.32, 90.39, 86.10. **IR(KBr)**: ν(C≡C): 2211 cm^−1^; ν(C=O): 1622 cm^−1^. **HRMS(ESI) *m*/*z*:** [M+H]^+^ calcd. for C_15_H_11_O_2_: 223.0753; Found: 223.0747.

(*E*)-1-(furan-2-yl)-5-phenylpent-1-en-4-yn-3-one (**3ka**), the general method using Triazine Ester **1l** and Phenylacetylene **2a** gave the title compound **3la** in 95% yield; ^1^H NMR (400 MHz, Acetonitrile-d_3_): δ/ppm = 7.75 (d, J = 15.9 Hz, 1H), 7.70–7.65 (m, 3H), 7.55–7.41 (m,3H), 6.95 (d, J = 3.6 Hz, 1H), 6.71–6.56 (m, 2H). ^13^C NMR (400 MHz, Acetonitrile-d_3_): δ/ppm = 177.22, 150.64, 146.68, 146.62, 134.30, 132.99, 130.96, 128.95, 125.55, 119.91, 118.03, 113.30, 90.43, 85.94. **IR(KBr)**: ν(C≡C): 2210 cm^−1^; ν(C=O): 1624 cm^−1^. **HRMS(ESI) *m*/*z*:** [M+H]^+^ calcd. for C_18_H_13_O_3_: 277.0859; Found: 277.0855.

(*E*)-2-methyl-1,5-diphenylpent-1-en-4-yn-3-one (**3ma**) [45], the general method using Triazine Ester **1m** and Phenylacetylene **2a** gave the title compound **3ma** in 78% yield; ^1^H NMR (400 MHz, Acetonitrile-d_3_): δ/ppm = 8.15(s, 1H), 7.71–7.67 (m, 2H), 7.60 (d, J = 7.3 Hz, 2H), 7.54–7.40 (m, 6H), 2.10 (d, J = 1.1 Hz, 3H). ^13^C NMR (400 MHz, Acetonitrile-d_3_): δ/ppm = 180.31, 145.48, 138.09, 135.50, 132.90, 130.86, 130.33, 129.47, 128.93, 128.71, 120.11, 91.37, 85.81, 11.61.

(*E*)-4-methyl-1-phenylhex-4-en-1-yn-3-one (**3na**) [12], the general method using Triazine Ester **1n** and Phenylacetylene **2a** gave the title compound **3na** in 91% yield; ^1^H NMR (400 MHz, Acetonitrile-d_3_): δ/ppm = 7.66–7.59 (m, 2H), 7.52–7.49 (m, 1H), 7.47–7.37 (m, 3H), 1.96 (d, J = 6.9 Hz, 3H), 1.81(s, 3H). ^13^C NMR (400 MHz, Acetonitrile-d_3_): δ/ppm = 179.71, 146.18, 139.19, 132.70, 130.71, 128.92, 120.16, 90.22, 85.66, 14.51, 9.44.

5-methyl-1-phenylhex-4-en-1-yn-3-one (**3oa**) [46], the general method using Triazine Ester **1o** and Phenylacetylene **2a** gave the title compound **3oa** in 87% yield; ^1^H NMR (400 MHz, Acetonitrile-d_3_): δ/ppm = 7.59–7.55 (m, 2H), 7.44 (t, J = 7.3 Hz, 1H), 7.37 (t, J = 7.3 Hz, 2H), 6.28(s, 1H), 2.28(s, 3H), 1.97(s, 3H). ^13^C NMR (400 MHz, Acetonitrile-d_3_): δ/ppm = 176.69, 158.34, 132.96, 130.46, 128.65, 126.26, 120.57, 90.42, 89.21, 28.06, 21.33.

(*E*)-1-phenyloct-4-en-1-yn-3-one (**3pa**) [47], the general method using Triazine Ester **1p** and Phenylacetylene **2a** gave the title compound **3pa** in 90% yield; ^1^H NMR (400 MHz, Acetonitrile-d_3_): δ/ppm = 7.67–7.62 (m, 2H), 7.56–7.49 (m, 1H), 7.49–7.43 (m, 2H), 7.36 (dt, J = 16.0, 6.9 Hz, 1H), 6.25–6.19 (m, 1H), 2.36–2.28 (m, 2H), 1.56 (h, J = 7.3 Hz, 2H), 0.96 (t, J = 7.3 Hz, 3H). ^13^C NMR (400 MHz, Acetonitrile-d_3_): δ/ppm = 178.16, 155.11, 132.88, 132.14, 130.92, 128.95, 119.89, 90.28, 85.76, 34.27, 20.96, 13.08.

(4*E*,6*E*)-1-phenylocta-4,6-dien-1-yn-3-one (**3qa**), the general method using Triazine Ester **1q** and Phenylacetylene **2a** gave the title compound **3qa** in 94% yield; ^1^H NMR (400 MHz, Acetonitrile-d_3_): δ/ppm = 7.70–7.65 (m, 2H), 7.64–7.50 (m,2H), 7.46 (t, J = 7.3 Hz, 2H), 6.53–6.33 (m, 2H), 6.20 (d, J = 15.6 Hz, 1H), 1.90 (d, J = 6.3 Hz, 3H). ^13^C NMR (400 MHz, Acetonitrile-d_3_): δ/ppm = 178.05, 149.21, 143.26, 132.90, 130.87, 129.93, 129.70, 128.93, 119.99, 90.15, 86.02, 18.28. **IR(KBr)**: ν(C≡C): 2210 cm^−1^; ν(C=O): 1619 cm^−1^. **HRMS(ESI) *m*/*z*:** [M+H]^+^ calcd. for C_14_H_13_O: 197.0961; Found: 197.0967.

(4*E*,6*E*)-1,7-diphenylhepta-4,6-dien-1-yn-3-one (**3ra**) [48], the general method using Triazine Ester **1r** and Phenylacetylene **2a** gave the title compound **3ra** in 88% yield; ^1^H NMR (400 MHz, Acetonitrile-d_3_): δ/ppm = 7.78 (dd, J = 11.3, 10.5 Hz, 1H), 7.73–7.68 (m, 2H), 7.59–7.52 (m, 3H), 7.51–7.45 (m, 2H), 7.43–7.35 (m, 3H), 7.26–7.09 (m, 2H), 6.41 (d, J = 15.3 Hz, 1H). ^13^C NMR (400 MHz, Acetonitrile-d_3_): δ/ppm = 177.68, 148.79, 143.06, 136.10, 132.96, 131.66, 130.94, 129.68, 129.06, 128.96, 127.61, 126.52, 120.00, 90.48, 86.18.

(*E*)-5-(4-methoxyphenyl)-1-phenylpent-1-en-4-yn-3-one (**3ab**) [7], the general method using Triazine Ester **1a** and 1-ethynyl-4-methoxybenzene **2b** gave the title compound **3ab** in 60% yield; ^1^H NMR (400 MHz, Acetonitrile-d_3_): δ/ppm = 7.97 (d, J = 16.1 Hz, 1H), 7.72 (dd, J = 6.5, 2.9 Hz, 2H), 7.67 (d, J = 8.8 Hz, 2H), 7.50–7.43 (m, 3H), 7.00 (d, J = 8.8 Hz, 2H), 6.88 (d, J = 16.1 Hz, 1H), 3.84(s, 3H). ^13^C NMR (400 MHz, Acetonitrile-d_3_): δ/ppm = 177.90, 161.94, 147.97, 135.16, 134.33, 131.17, 129.14, 128.85, 128.64, 114.64, 111.51, 91.92, 85.96, 55.38.

(*E*)-1-phenyl-5-(p-tolyl)pent-1-en-4-yn-3-one (**3ac**) [41], the general method using Triazine Ester **1a** and 4-ethynyltoluene **2c** gave the title compound **3ac** in 95% yield; ^1^H NMR (400 MHz, Acetonitrile-d_3_): δ/ppm = 7.99 (d, J = 16.2 Hz, 1H), 7.72 (dd, J = 6.7, 2.9 Hz, 2H), 7.61 (d, J = 8.1 Hz, 2H), 7.47 (dd, J = 5.2, 2.0 Hz, 3H), 7.29 (d, J = 8.0 Hz, 2H), 6.90 (d, J = 16.1 Hz, 1H), 2.39(s, 3H). ^13^C NMR (400 MHz, Acetonitrile-d_3_): δ/ppm = 177.93, 148.33, 141.96, 134.27, 133.08, 131.25, 129.66, 129.15, 128.89, 128.59, 116.79, 91.38, 85.91, 20.84.

(*E*)-5-(4-ethylphenyl)-1-phenylpent-1-en-4-yn-3-one (**3ad**), the general method using Triazine Ester **1a** and 4-Ethylphenylacetylene **2d** gave the title compound **3ad** in 61% yield; ^1^H NMR (400 MHz, Acetonitrile-d_3_): δ/ppm = 8.00 (d, J = 16.2 Hz, 1H), 7.72 (dd, J = 6.5, 2.9 Hz, 2H), 7.64 (d, J = 8.1 Hz, 2H), 7.47 (dd, J = 5.1, 1.7 Hz, 3H), 7.32 (d, J = 8.1 Hz, 2H), 6.90 (d, J = 16.2 Hz, 1H), 2.70 (q, J = 7.6 Hz, 2H), 1.23 (t, J = 7.6 Hz, 3H). ^13^C NMR (400 MHz, Acetonitrile-d_3_): δ/ppm = 177.94, 148.35, 148.13, 134.28, 133.21, 131.25, 129.15, 128.89, 128.59, 128.53, 117.04, 91.39, 85.90, 28.64, 14.73. **IR(KBr)**: ν(C≡C): 2208 cm^−1^; ν(C=O): 1625 cm^−1^. **HRMS(ESI) *m*/*z*:** [M+H]^+^ calcd. for C_19_H_17_O: 261.1274; Found: 261.1277.

(*E*)-5-(4-butylphenyl)-1-phenylpent-1-en-4-yn-3-one (**3ae**), the general method using Triazine Ester **1a** and 1-Butyl-4-eth-1-ynylbenzene **2e** gave the title compound **3ae** in 90% yield; ^1^H NMR (400 MHz, CDCl_3_): δ/ppm = 7.91 (d, J = 16.1 Hz, 1H), 7.63–7.54 (m, 4H), 7.46–7.41 (m, 3H), 7.23 (d, J = 8.0 Hz, 2H), 6.87 (d, J = 16.1 Hz, 1H), 2.65 (t, J = 7.7 Hz, 2H), 1.61 (p, J = 7.3 Hz, 2H), 1.37 (hept, J = 6.8 Hz, 2H), 0.94 (t, J = 7.3 Hz, 3H). ^13^C NMR (400 MHz, CDCl_3_): δ/ppm = 178.45, 148.20, 146.41, 134.21, 133.12, 131.21, 129.18, 128.93, 128.78, 128.72, 117.33, 92.42, 86.55, 35.87, 33.35, 22.40, 14.02. **IR(KBr)**: ν(C≡C): 2194 cm^−1^; ν(C=O): 1627 cm^−1^. **HRMS(ESI) *m*/*z*:** [M+H]^+^ calcd. for C_21_H_21_O: 289.1587; Found: 289.1585.

(*E*)-1-phenyl-5-(m-tolyl)pent-1-en-4-yn-3-one (**3af**) [4], the general method using Triazine Ester **1a** and 1-Ethynyl-3-methylbenzene **2f** gave the title compound **3af** in 87% yield; ^1^H NMR (400 MHz, Acetonitrile-d_3_): δ/ppm = 8.00 (d, J = 16.1 Hz, 1H), 7.72 (dd, J = 6.5, 2.9 Hz, 2H), 7.56–7.44 (m, 5H), 7.38–7.34 (m, 2H), 6.90 (d, J = 16.1 Hz, 1H), 2.37(s, 3H). ^13^C NMR (400 MHz, Acetonitrile-d_3_): δ/ppm = 177.93, 148.51, 139.02, 134.25, 133.43, 131.85, 131.29, 130.15, 129.16, 128.91, 128.86, 128.57, 119.74, 91.09, 85.82, 20.28.

(*E*)-5-(4-bromophenyl)-1-phenylpent-1-en-4-yn-3-one (**3ag**) [49], the general method using Triazine Ester **1a** and 1-bromo-4-ethynylbenzene **2g** gave the title compound **3ag** in 75% yield; ^1^H NMR (400 MHz, CDCl_3_): δ/ppm = 7.89 (d, J = 16.1 Hz, 1H), 7.61 (dd, J = 6.5, 2.8 Hz, 2H), 7.56 (d, J = 8.5 Hz, 2H), 7.51 (d, J = 8.5 Hz, 2H), 7.44 (dd, J = 5.1, 1.7 Hz, 3H), 6.87 (d, J = 16.1 Hz, 1H). ^13^C NMR (400 MHz, CDCl_3_): δ/ppm = 178.08, 148.65, 134.32, 134.04, 132.17, 131.41, 129.22, 128.84, 128.45, 125.49, 119.21, 90.23, 87.51.

(*E*)-5-(4-chlorophenyl)-1-phenylpent-1-en-4-yn-3-one (**3ah**) [49], the general method using Triazine Ester **1a** and 1-chloro-4-ethynylbenzene **2h** gave the title compound **3ag** in 90% yield; ^1^H NMR (400 MHz, CDCl_3_): δ/ppm = 7.89 (d, J = 16.1 Hz, 1H), 7.64–7.56 (m, 4H), 7.47–7.38 (m, 5H), 6.87 (d, J = 16.1 Hz, 1H). ^13^C NMR (400 MHz, CDCl_3_): δ/ppm = 178.09, 148.62, 137.09, 134.22, 134.05, 131.39, 129.55–129.09 (m), 128.83, 128.47, 118.75, 90.20, 87.40.

(*E*)-1-phenyl-5-(4-(trifluoromethyl)phenyl)pent-1-en-4-yn-3-one (**3ai**) [4], the general method using Triazine Ester **1a** and 1-Ethynyl-4-(trifluoromethyl)benzene **2i** gave the title compound **3ai** in 86% yield; ^1^H NMR (400 MHz, Acetonitrile-d_3_): δ/ppm = 8.03 (d, J = 16.0 Hz, 1H), 7.88 (d, J = 8.0 Hz, 2H), 7.78 (d, J = 8.1 Hz, 2H), 7.73 (m, 2H), 7.50–7.43 (m, 3H), 6.93 (d, J = 16.1 Hz, 1H). ^13^C NMR (400 MHz, Acetonitrile-d_3_): δ/ppm = 177.65, 149.26, 134.14, 133.53, 131.46, 129.18, 128.99, 128.33, 125.74 (d, J = 11.5 Hz), 124.16, 88.39, 87.30.

(*E*)-5-(4-fluorophenyl)-1-phenylpent-1-en-4-yn-3-one (**3aj**) [49], the general method using Triazine Ester **1a** and 1-fluoro-4-ethynylbenzene **2j** gave the title compound **3aj** in 76% yield; ^1^H NMR (400 MHz, Acetonitrile-d_3_): δ/ppm = 8.00 (d, J = 16.1 Hz, 1H), 7.80–7.71 (m, 4H), 7.47 (m, 3H), 7.26–7.19 (m, 2H), 6.90 (d, J = 16.1 Hz, 1H). ^13^C NMR (400 MHz, Acetonitrile-d_3_): δ/ppm = 177.83, 164.02 (d, J = 249.8 Hz), 148.64, 135.64 (d, J = 9.1 Hz), 134.23, 131.32, 129.16, 128.92, 128.47, 116.27 (d, J = 22.6 Hz), 89.72, 85.92.

(*E*)-5-(2-chlorophenyl)-1-phenylpent-1-en-4-yn-3-one (**3ak**), the general method using Triazine Ester **1a** and 1-chloro-2-ethynylbenzene **2k** gave the title compound **3ak** in 93% yield; ^1^H NMR (400 MHz, CDCl_3_): δ/ppm = 8.14 (d, J = 16.1 Hz, 1H), 7.68 (dd, J = 7.6, 1.5 Hz, 1H), 7.60 (dd, J = 6.5, 2.8 Hz, 2H), 7.50 (d, J = 8.0 Hz, 1H), 7.46–7.38 (m, 4H), 7.34–7.29 (m, 1H), 6.88 (d, J = 16.1 Hz, 1H). ^13^C NMR (400 MHz, CDCl_3_): δ/ppm = 178.38, 149.90, 137.47, 135.06, 134.19, 131.80, 131.41, 129.75, 129.22, 128.84, 128.65, 127.01, 120.58, 90.61, 87.77. **IR(KBr)**: ν(C≡C): 2213 cm^−1^; ν(C=O): 1630 cm^−1^. **HRMS(ESI) *m*/*z*:** [M+H]^+^ calcd. for C_17_H_11_OCl: 267.0571; Found: 267.0568.

(*E*)-5-([1,1′-biphenyl]-4-yl)-1-phenylpent-1-en-4-yn-3-one (**3al**) [4], the general method using Triazine Ester **1a** and 4-ethynyl-1,1′-biphenyl **2l** gave the title compound **3al** in 86% yield; ^1^H NMR (400 MHz, CDCl_3_): δ/ppm = 7.94 (d, J = 16.1 Hz, 1H), 7.74 (d, J = 8.3 Hz, 2H), 7.68–7.60 (m, 6H), 7.51–7.37 (m, 6H), 6.90 (d, J = 16.1 Hz, 1H). ^13^C NMR (400 MHz, CDCl_3_): δ/ppm = 178.33, 148.41, 143.54, 139.89, 134.18, 133.58, 131.30, 129.21, 129.10, 128.83, 128.66, 128.29, 127.43, 127.24, 119.00, 91.76, 87.42.

(*E*)-1-phenylnon-1-en-4-yn-3-one (**3am**) [50], the general method using Triazine Ester **1a** and hex-1-yne **2m** gave the title compound **3am** in 80% yield; ^1^H NMR (400 MHz, Acetonitrile-d_3_): δ/ppm = 7.87 (d, J = 16.1 Hz, 1H), 7.73–7.60 (m, 2H), 7.45 (dd, J = 5.1, 2.0 Hz, 3H), 6.79 (d, J = 16.1 Hz, 1H), 2.50 (t, J = 7.1 Hz, 2H), 1.63 (dt, J = 14.8, 7.1 Hz, 2H), 1.49 (dq, J = 14.4, 7.2 Hz, 2H), 0.96 (t, J = 7.3 Hz, 3H). ^13^C NMR (400 MHz, Acetonitrile-d_3_): δ/ppm = 174.23, 170.70, 162.38, 149.11, 133.75, 131.40, 129.17, 128.68, 115.88, 56.01.

(*E*)-6,6-dimethyl-1-phenylhept-1-en-4-yn-3-one (**3an**) [51], the general method using Triazine Ester **1a** and 3,3-dimethylbut-1-yne **2n** gave the title compound **3an** in 42% yield; ^1^H NMR (400 MHz, Acetonitrile-d_3_): δ/ppm = 7.84 (d, J = 16.1 Hz, 1H), 7.70–7.65 (m, 2H), 7.46 (dd, J = 5.1, 1.7 Hz, 3H), 6.79 (d, J = 16.1 Hz, 1H), 1.36 (s, 9H). ^13^C NMR (400 MHz, Acetonitrile-d_3_): δ/ppm = 178.36, 148.16, 134.22, 131.15, 129.13, 128.79, 128.69, 102.04, 77.23, 29.38, 27.69.

## 4. Conclusions

This report presents a diversity-oriented synthesis of cross-conjugated enynones through Pd-catalyzed Sonogashira coupling using triazine esters as acyl electrophiles. The study demonstrates that many triazine esters can produce cross-conjugated enynones with high-to-excellent yields under base- and phosphine-ligand-free conditions. The proposed mechanism for the reaction provides a better understanding of the reaction pathway and highlights the significance of triazine-stabilized intermediates in the Pd-catalyzed Sonogashira coupling reaction.

Overall, this method can potentially expand the possibilities of triazine-mediated C-O activation in various fields. Further research is needed to investigate the full potential of this Pd-catalyzed transformation of triazine ester electrophiles, with possible future studies focusing on more challenging substrates and the development of new earth-abundant metal–catalyst systems.

## Figures and Tables

**Table 1:** The optimization of the palladium-catalyzed cross-coupling of cinnamic triazine ester **1a** and phenylacetylene **3a**.

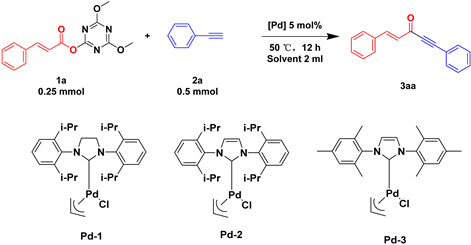			
Entry	Catalyst	Solvent	Yield [%] ^a^
1	Pd(OAc)_2_	Toluene	10
2	Pd(OAc)_2_	1,4-Dioxane	16
3	Pd(OAc)_2_	THF	13
4	Pd(OAc)_2_	DMF	N.D.
5	Pd(OAc)_2_	Anisole	6
6	Pd(OAc)_2_	MeCN	42
7	PdCl_2_	MeCN	2
8	PdCl_2_(PPh)_2_	MeCN	N.D.
9	[(Cinnamyl)PdCl]_2_	MeCN	62
10	[(2-Methylallyl)PdCl]_2_	MeCN	60
11	[(allyl)PdCl]_2_	MeCN	58
12	Pd-1	MeCN	75
13	Pd-2	MeCN	78
14	Pd-3	MeCN	96
15 ^b^	Pd-3	MeCN	95
16 ^c^	Pd-3	MeCN	95

^a^ All reactions were performed on a 0.25 mmol scale in 2 mL of solvent, and yields were determined by calibrated GC analysis with biphenyl as an internal standard. ^b^ catalyst (3% mol) was used. ^c^ catalyst (1% mol) was used, 24 h.

## Data Availability

Data are contained within the article. The data can be found in the Supplementary Materials.

## Supplementary Materials

The following supporting information can be downloaded at: https://www.mdpi.com/article/10.3390/molecules28114364/s1. This file contains details about the spectrum of the product.

## Sample Availability

Samples of the compounds are available from the authors.
