# Supplementary material for: A General Synthesis of Cross-Conjugated Enynones through Pd Catalyzed Sonogashira Coupling with Triazine Esters
Source: Molecules. 2023 May 26;28(11):4364. doi: 10.3390/molecules28114364 (PMC10254696; doi:10.3390/molecules28114364)

SUPPORTING INFORMATION:

A General Synthesis of Cross-conjugated enynones through Pd Catalyzed Sonogashira Coupling with Triazine Esters

Dezhi Lin [1], Yunfang Liu [2]，Hongyu Yang [1], Xiao Zhang [1], Huaming Sun [1], Yajun Jian [1], Weiqiang Zhang [1,*], Jianming Yang [3], Ziwei Gao [1]

[1] Key Laboratory of Applied Surface and Colloid Chemistry (MOE), Xi'an Key Laboratory of Organometallic Material Chemistry, School of Chemistry and Chemical Engineering, Shaanxi Normal University, Xi'an, 710119, P. R. China.
[2] South China Institute of Environmental Science, Ministry of Ecology and Environment, 510655, Guangzhou, China.
[3] Xi'an Modern Chemistry Research Institute, Xi'an 710065, China
* Correspondence: zwq@snnu.edu.cn.

Contents:

Page

Characteristic spectra of product en-eynones..2-32

^{1}H NMR of **3aa**

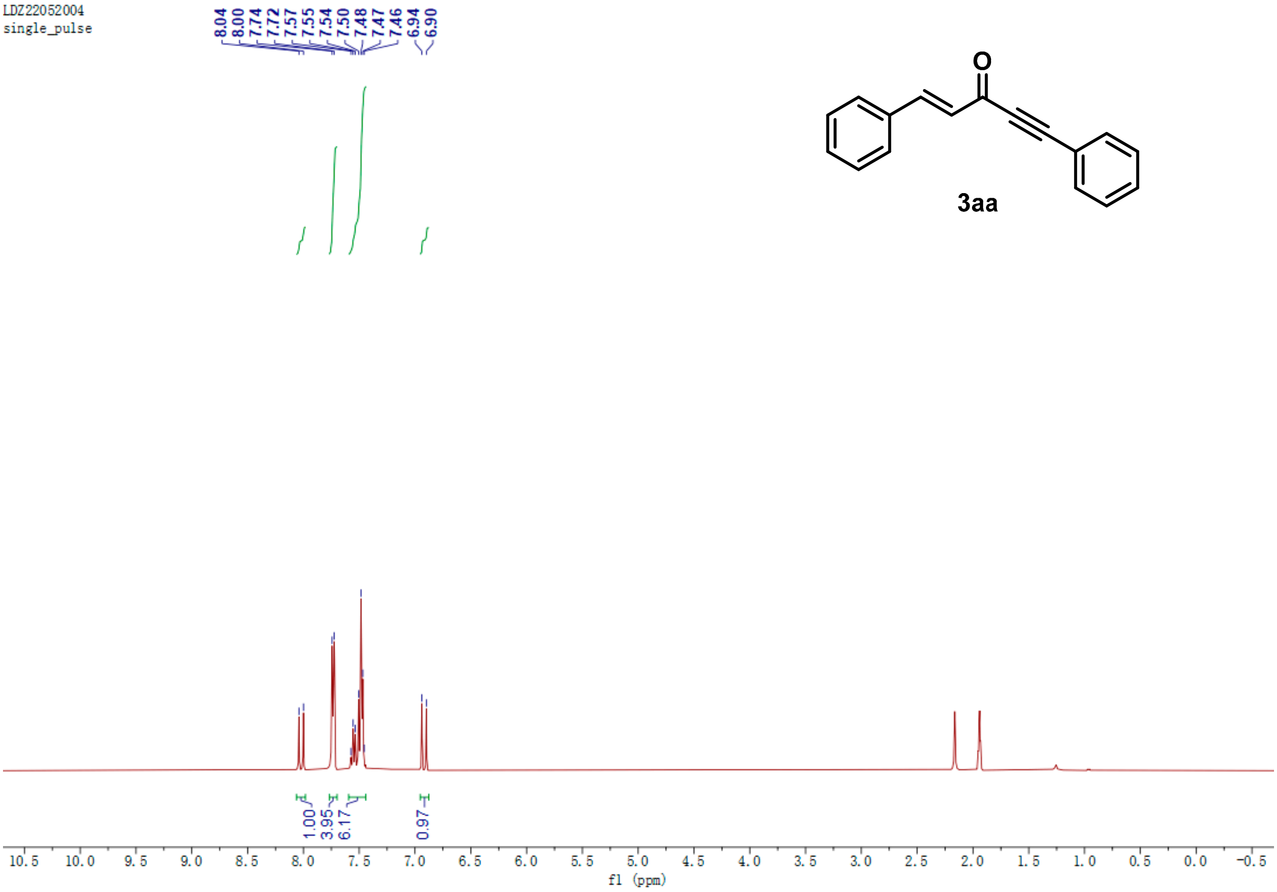

^{13}C NMR of **3aa**

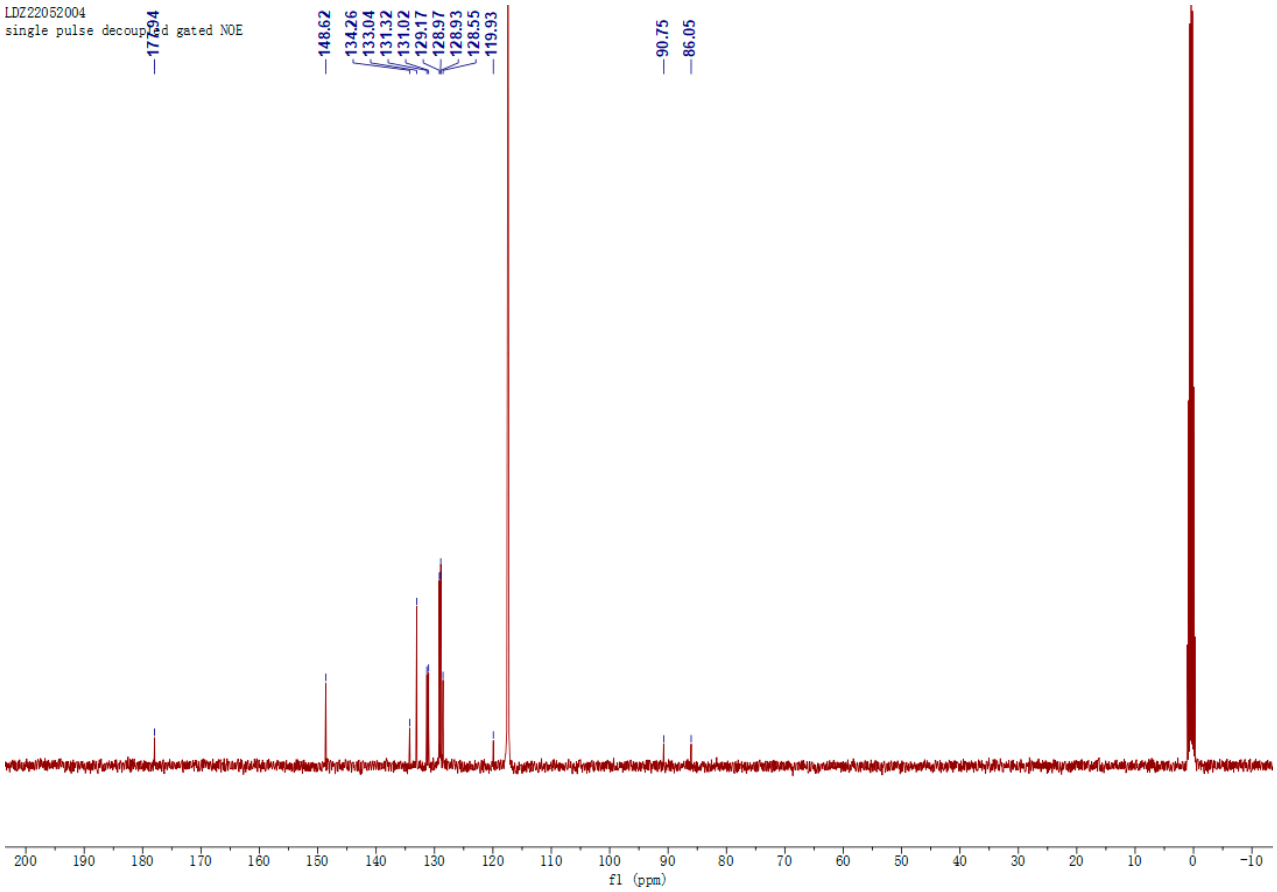

^{1}H NMR of **3ba**

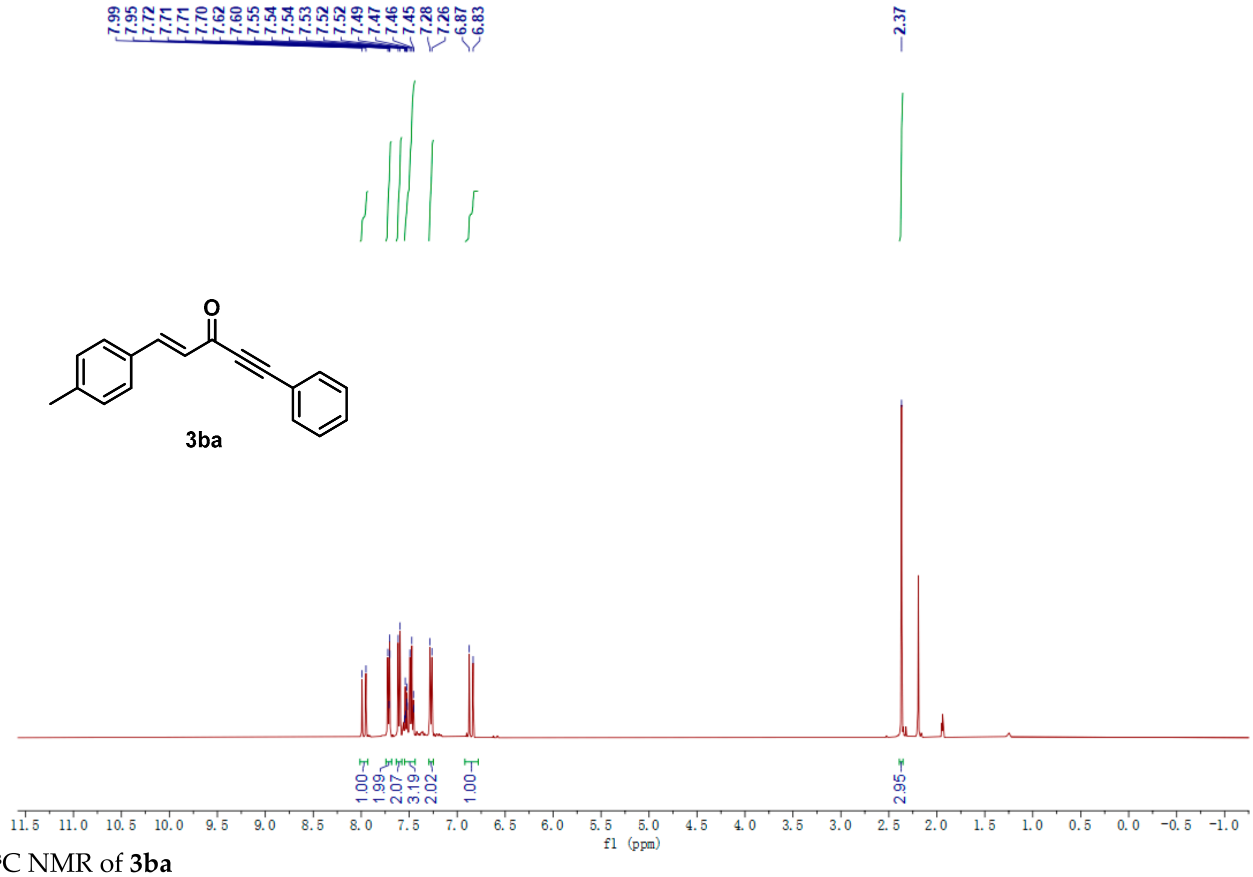

^{13}C NMR of **3ba**

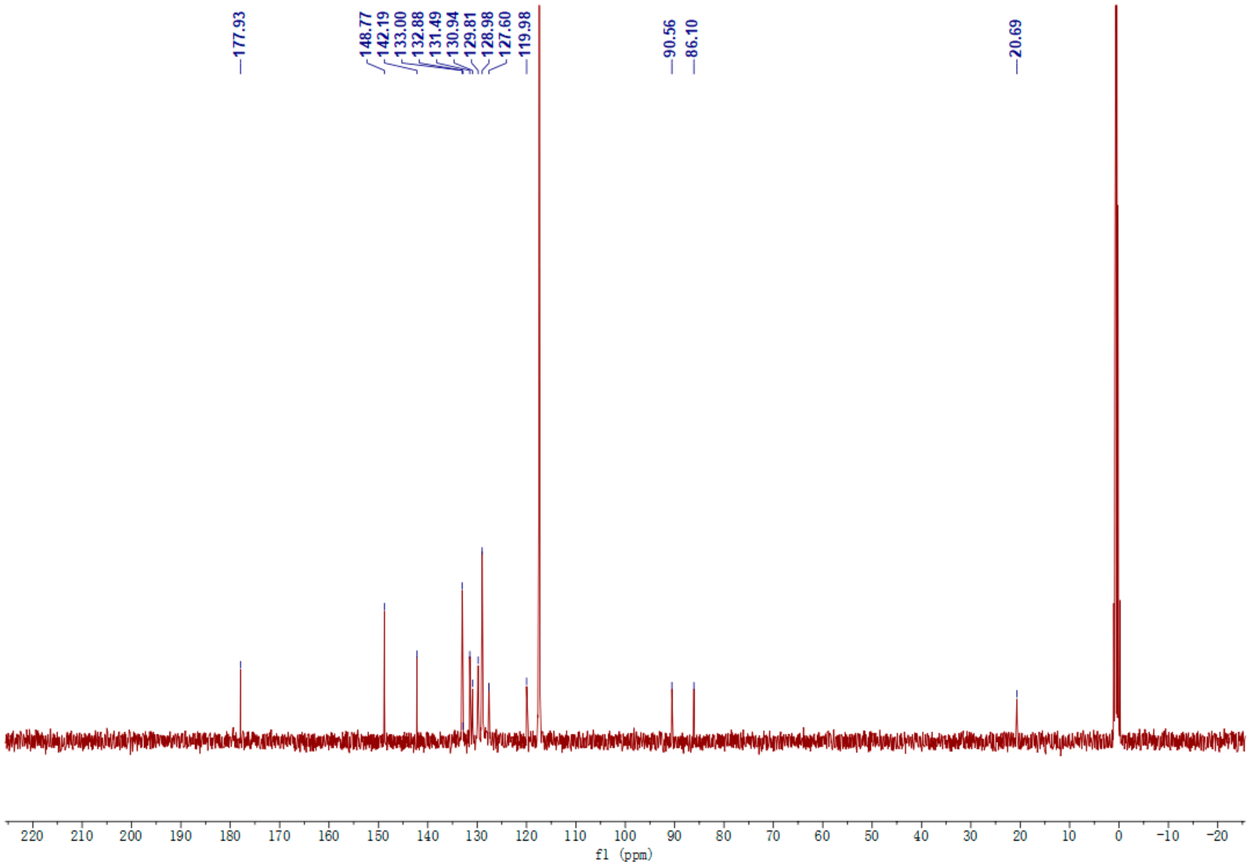

^{1}H NMR of **3ca**

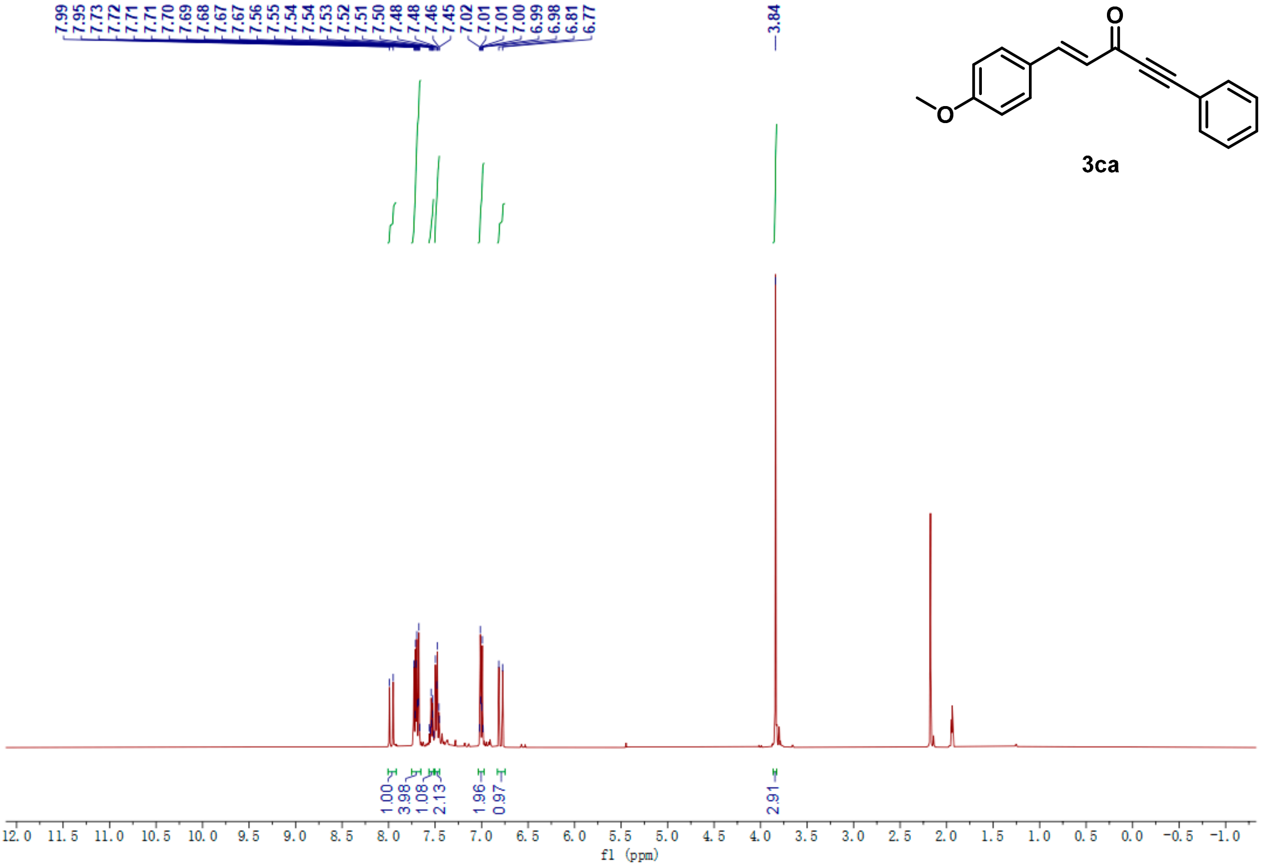

^{13}C NMR of **3ca**

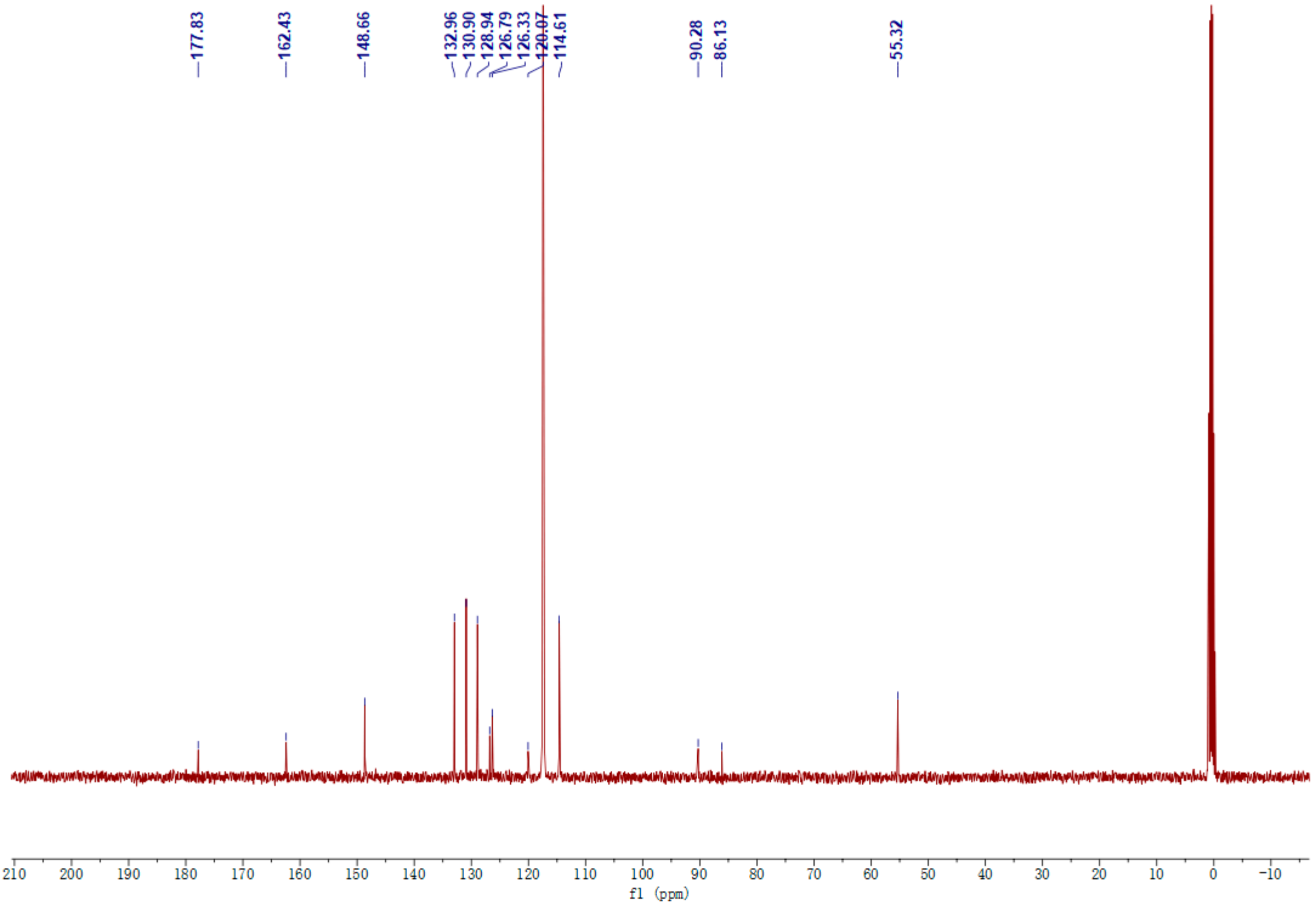

^{1}H NMR of **3da**

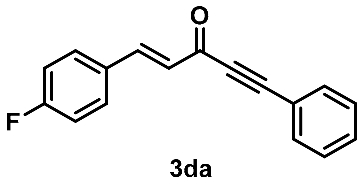

3da

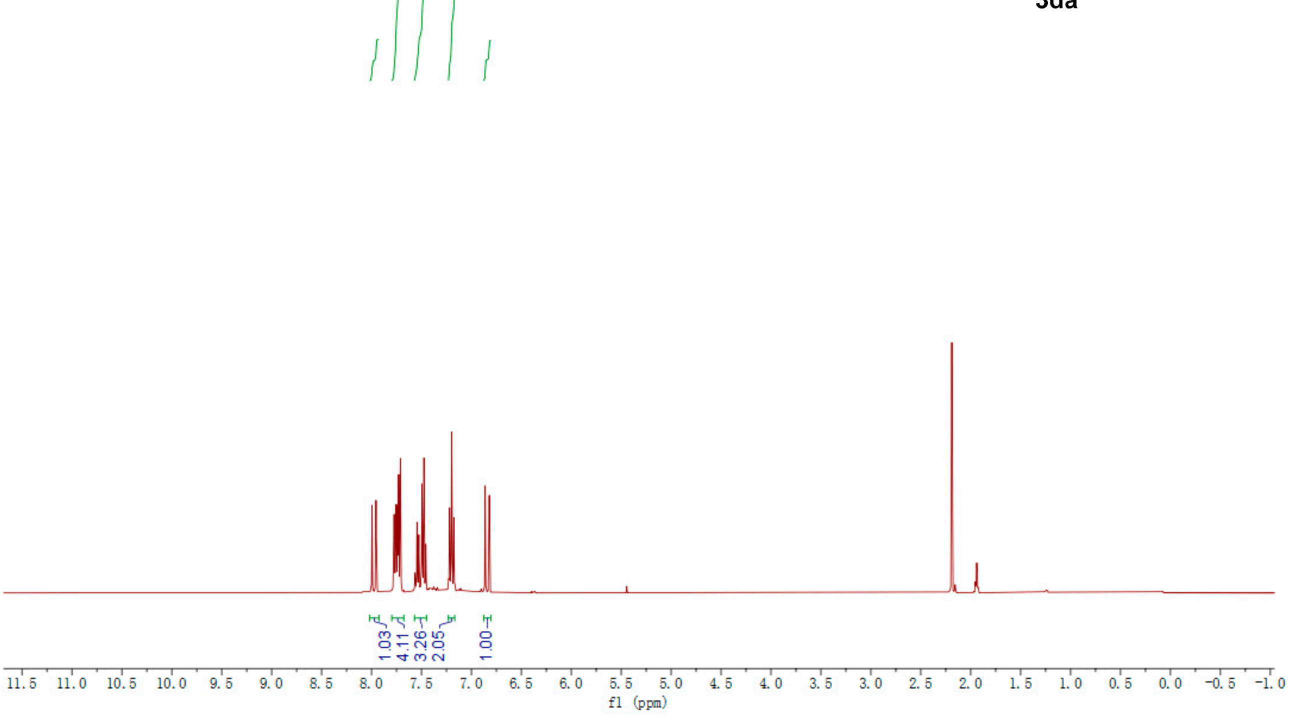

^{13}C NMR of **3da**

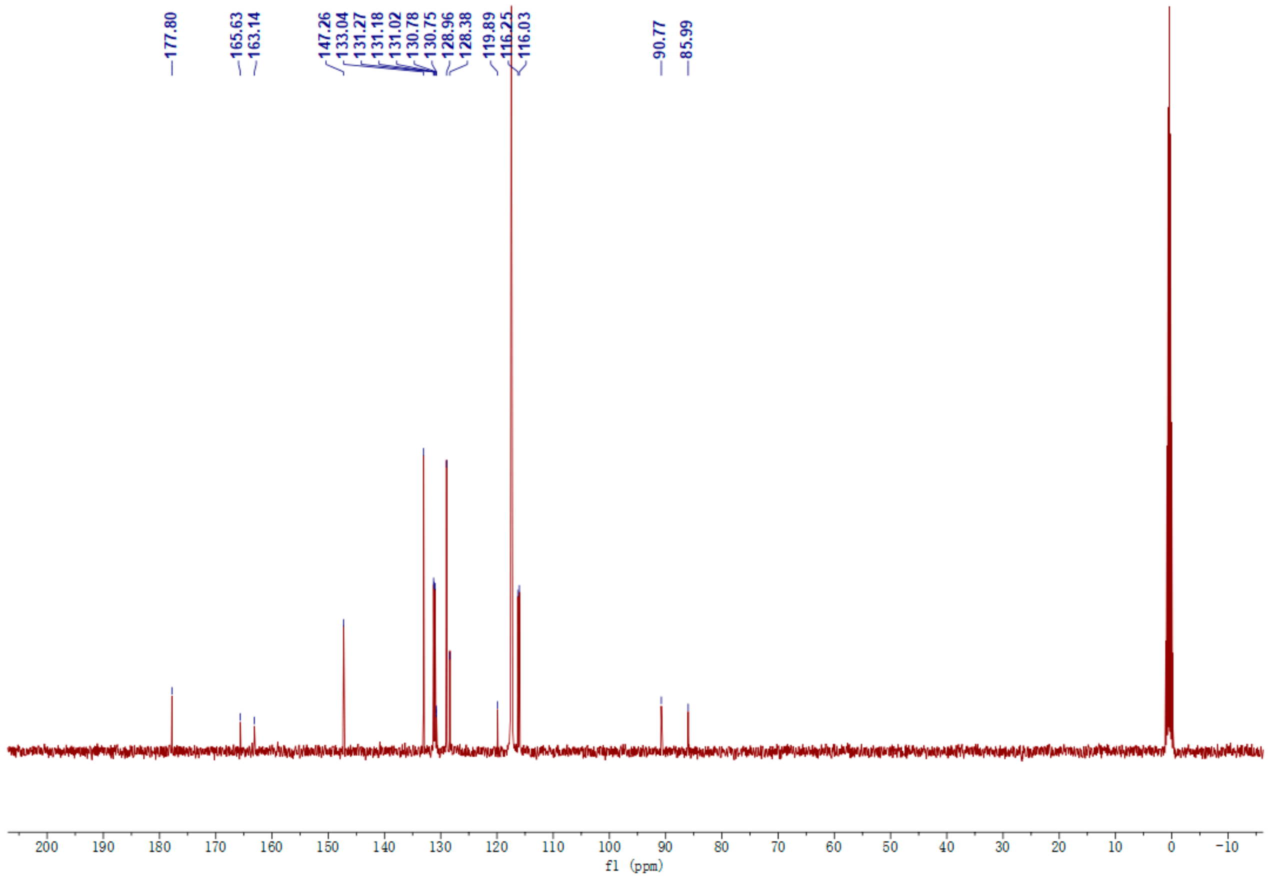

^{1}H NMR of **3ea**

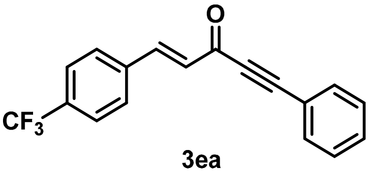

3ea

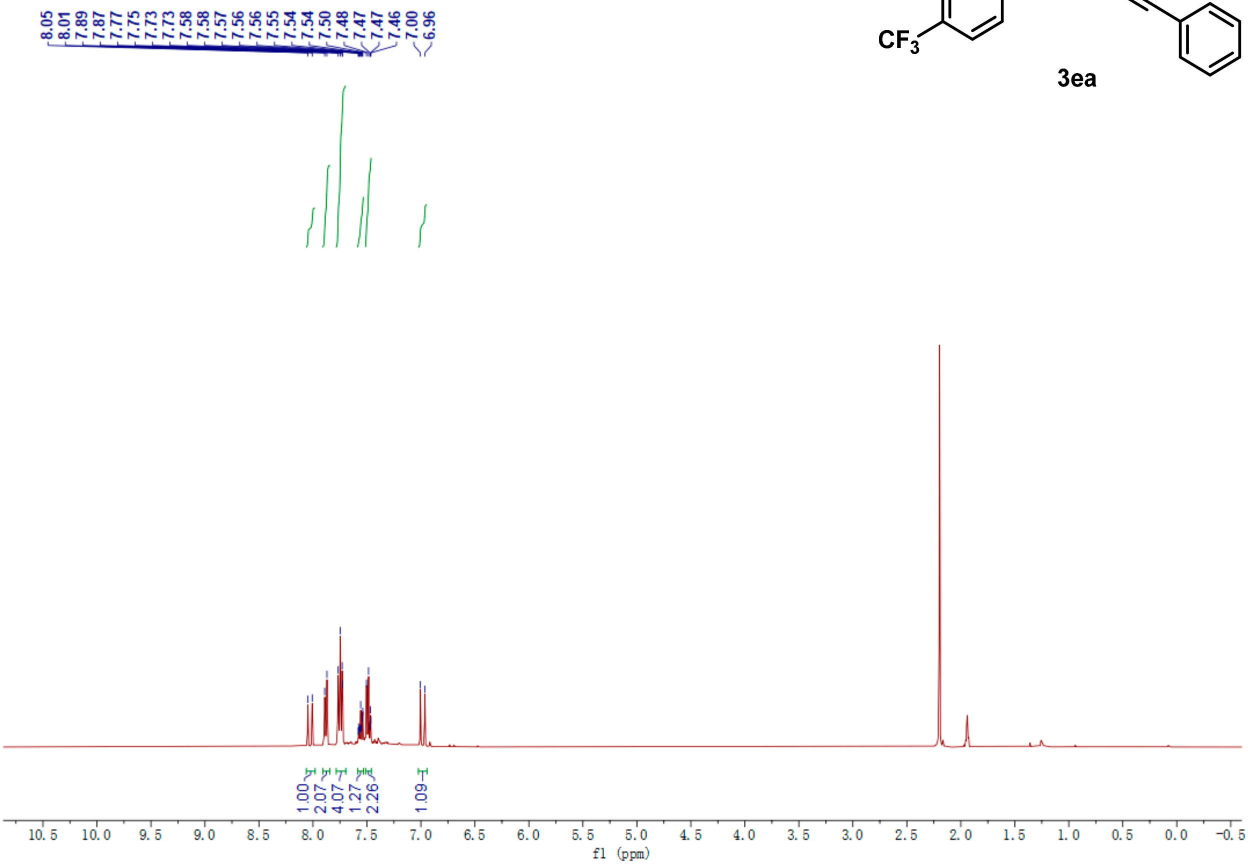

^{13}C NMR of **3ea**

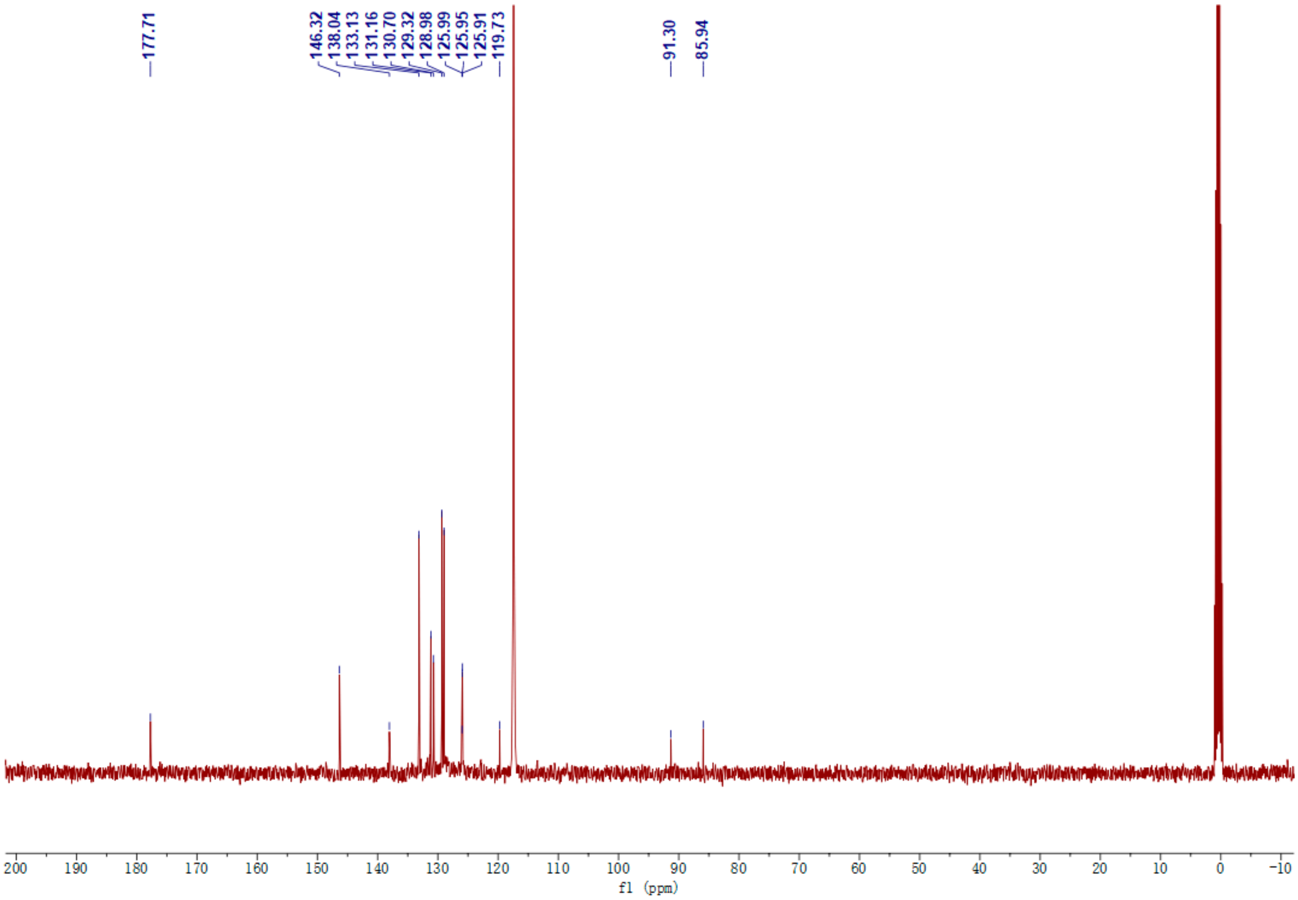

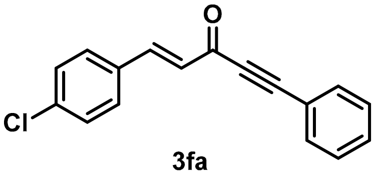

3fa

^{1}H NMR of **3fa**

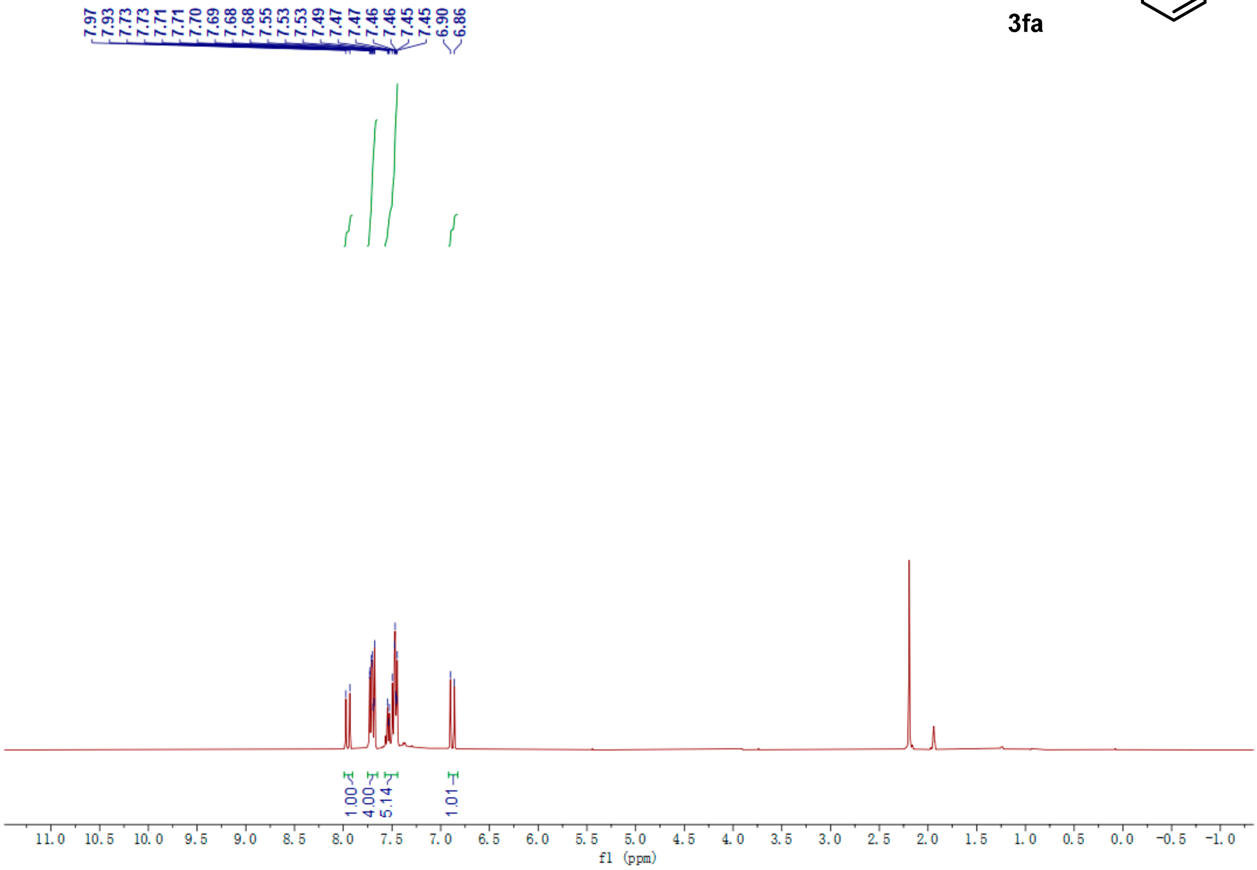

^{13}C NMR of **3fa**

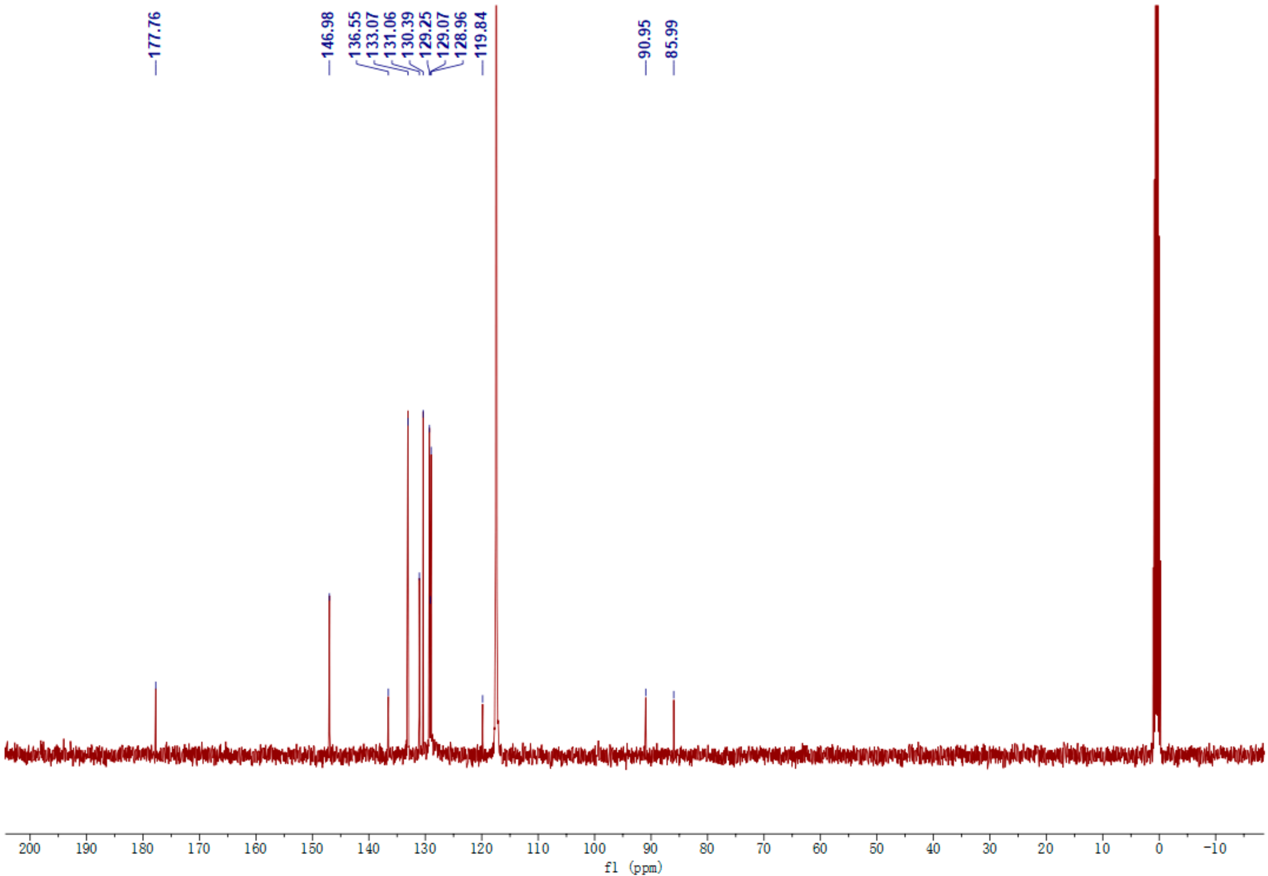

^{1}H NMR of **3ga**

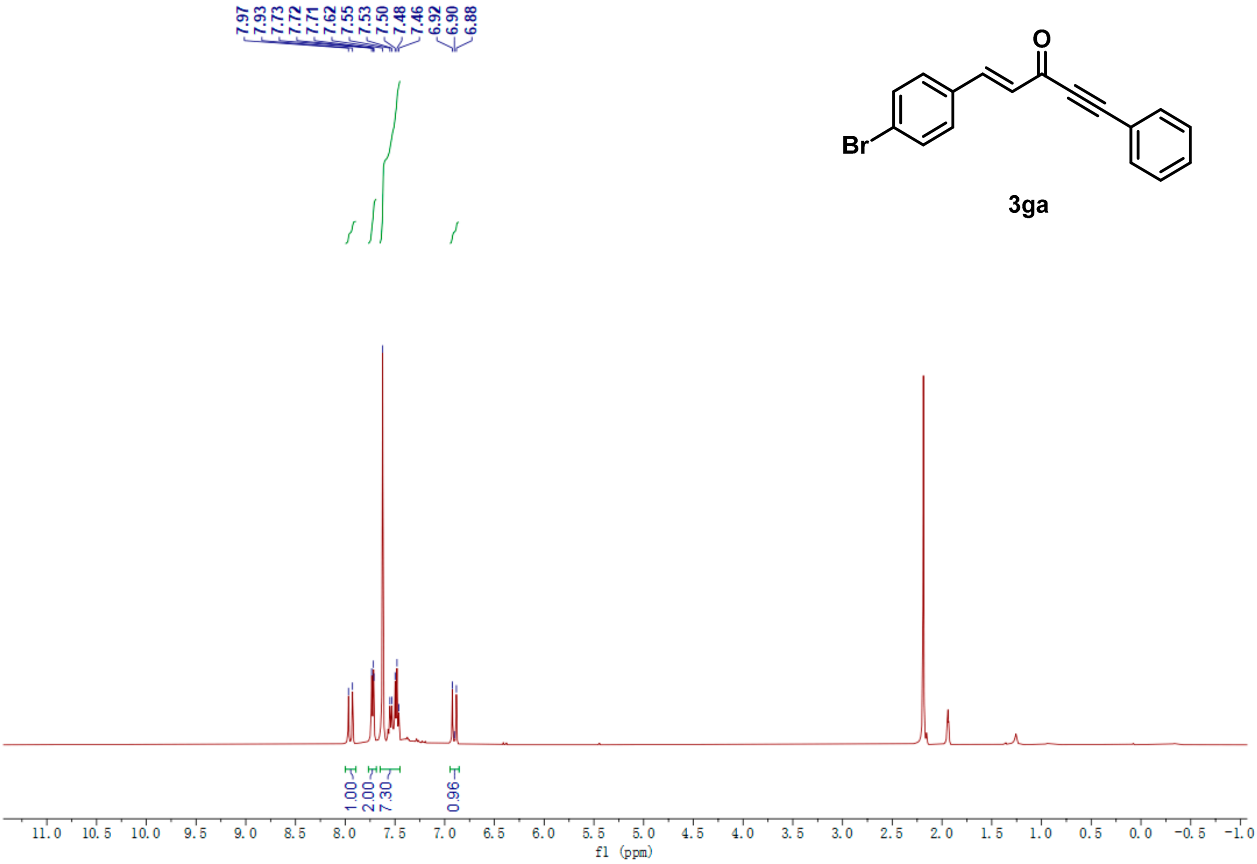

^{13}C NMR of **3ga**

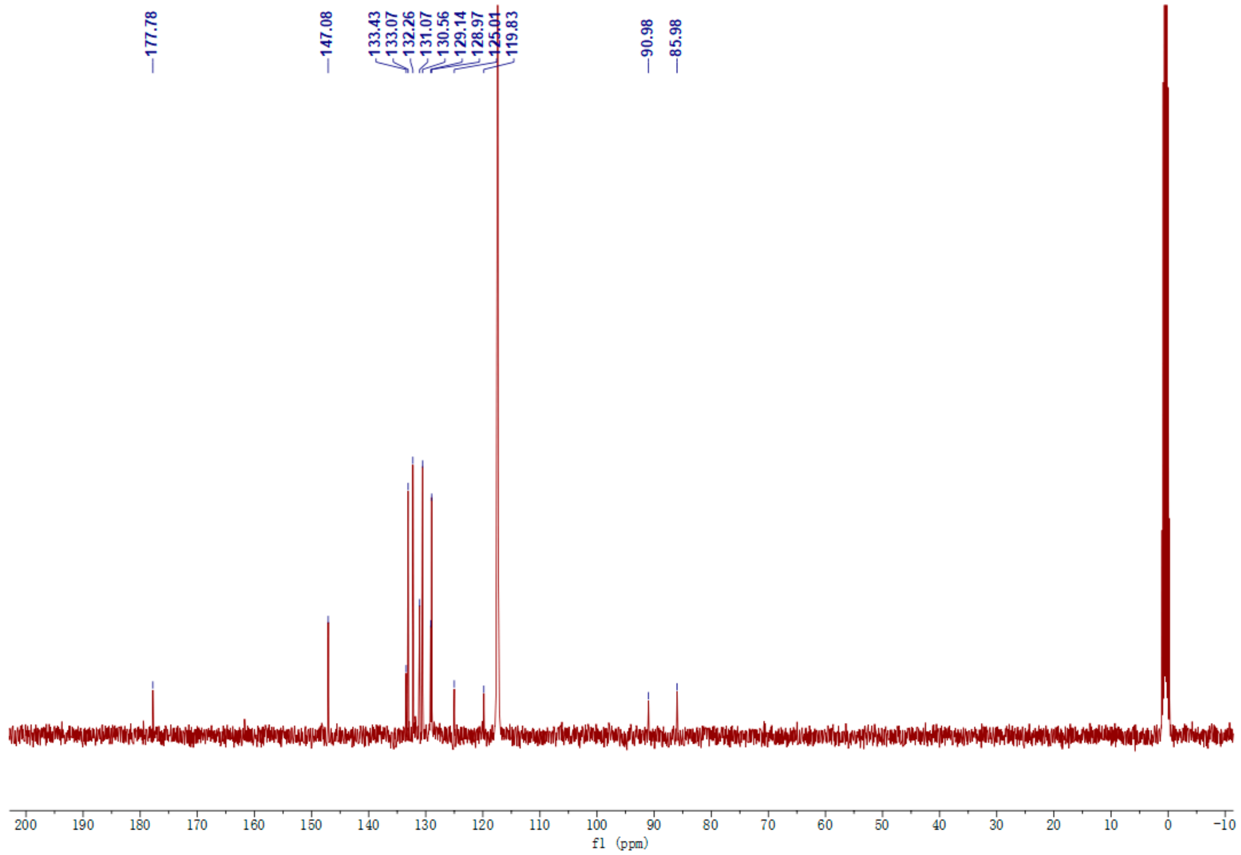

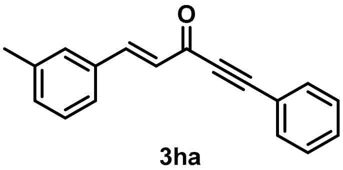

3ha

^{1}H NMR of **3ha**

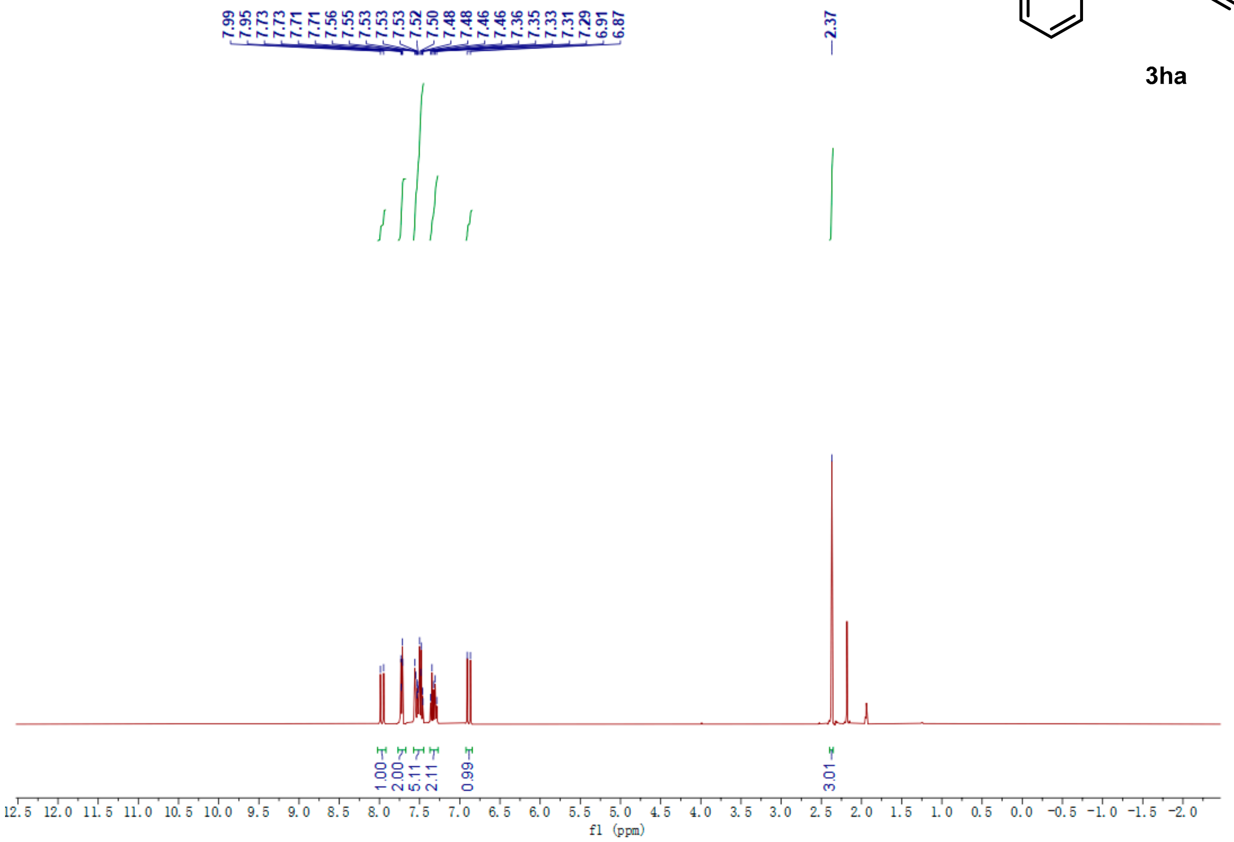

^{13}C NMR of **3ha**

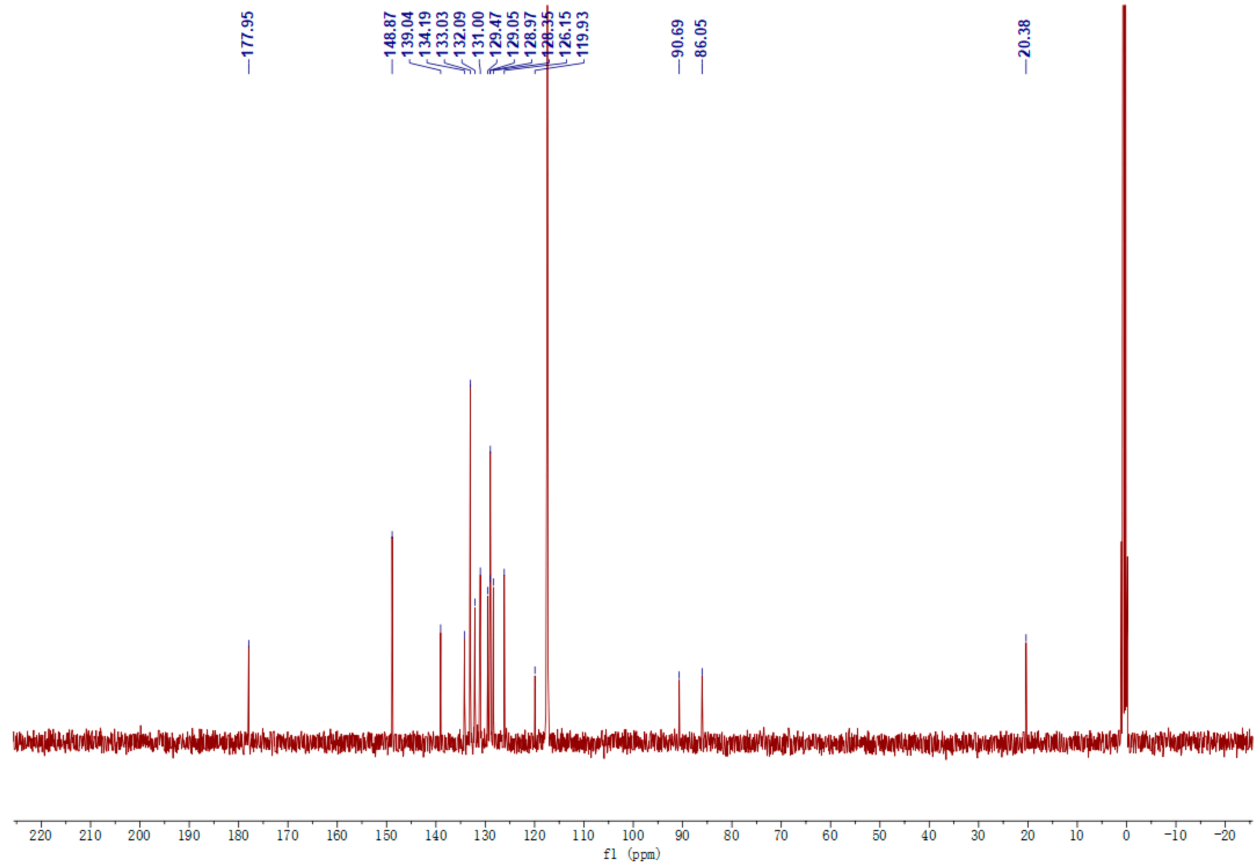

IR of **3ha**

IR spectra data: **IR(KBr)**: ν 3049, 3031, 2923, 2212, 1627, 1487, 1440, 1305, 1279, 1175, 1161, 971, 865, 780, 759, 687, 584, 537, 429 cm^{-1}.

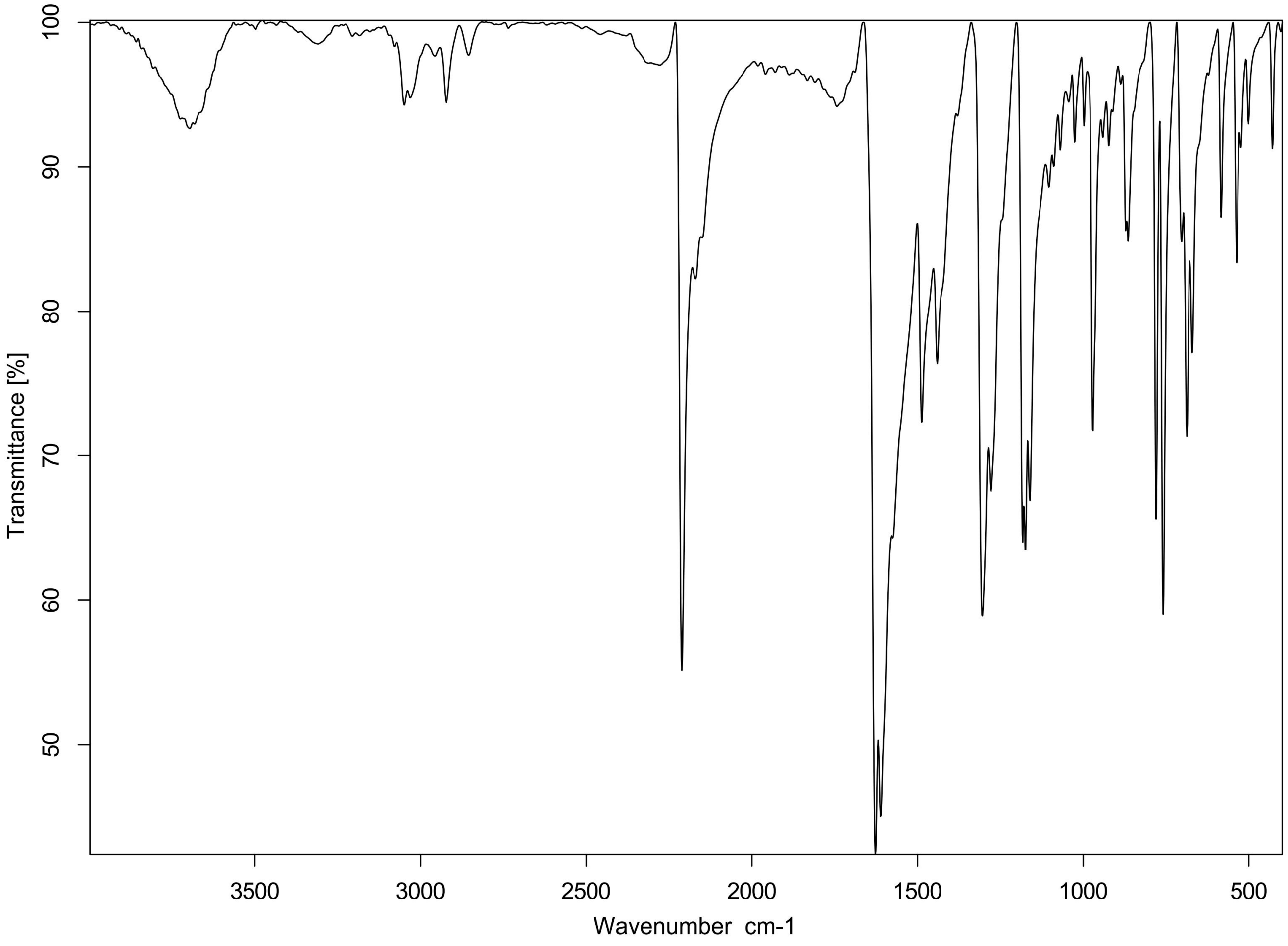

^{1}H NMR of **3ia**

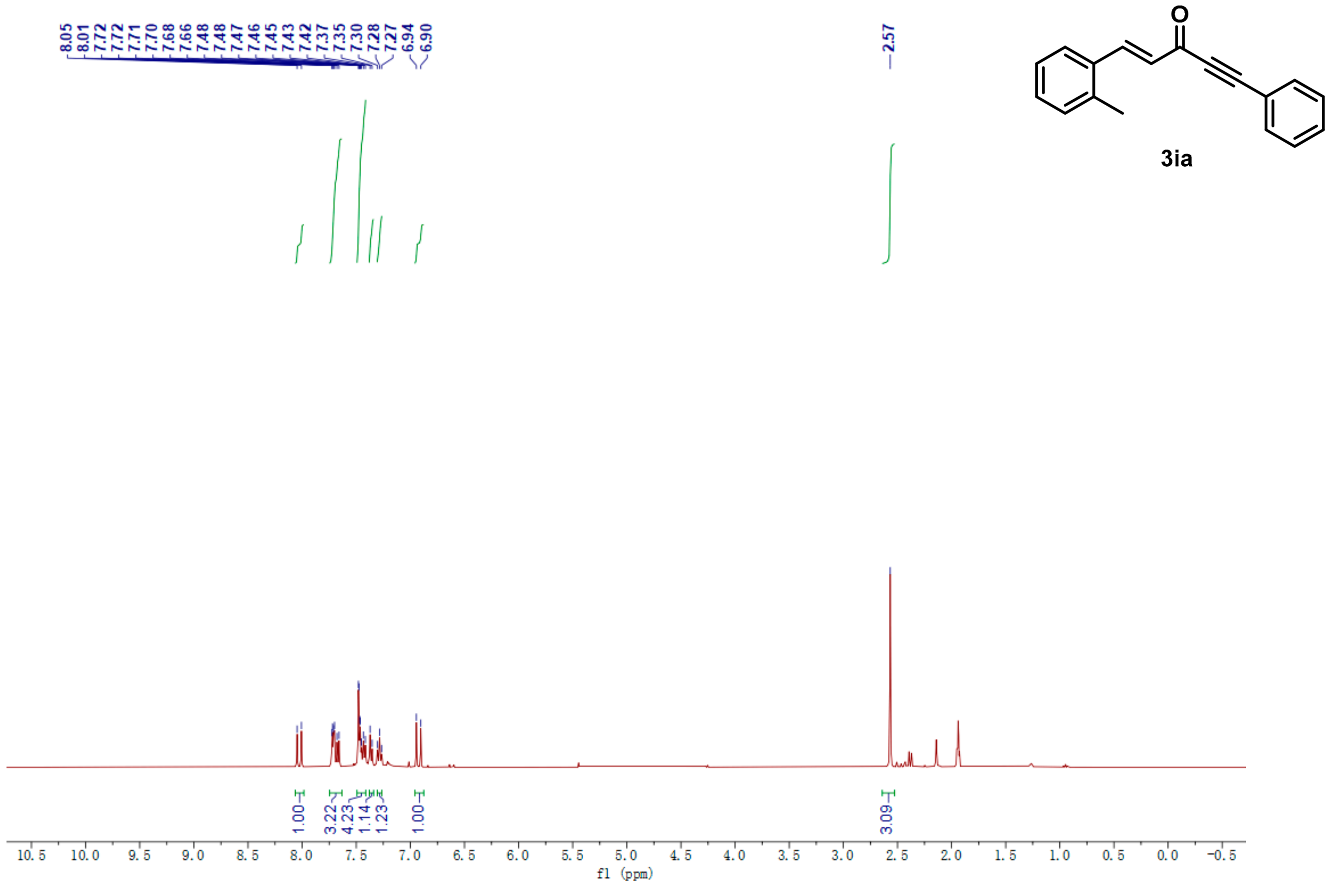

3ia

^{13}C NMR of **3ia**

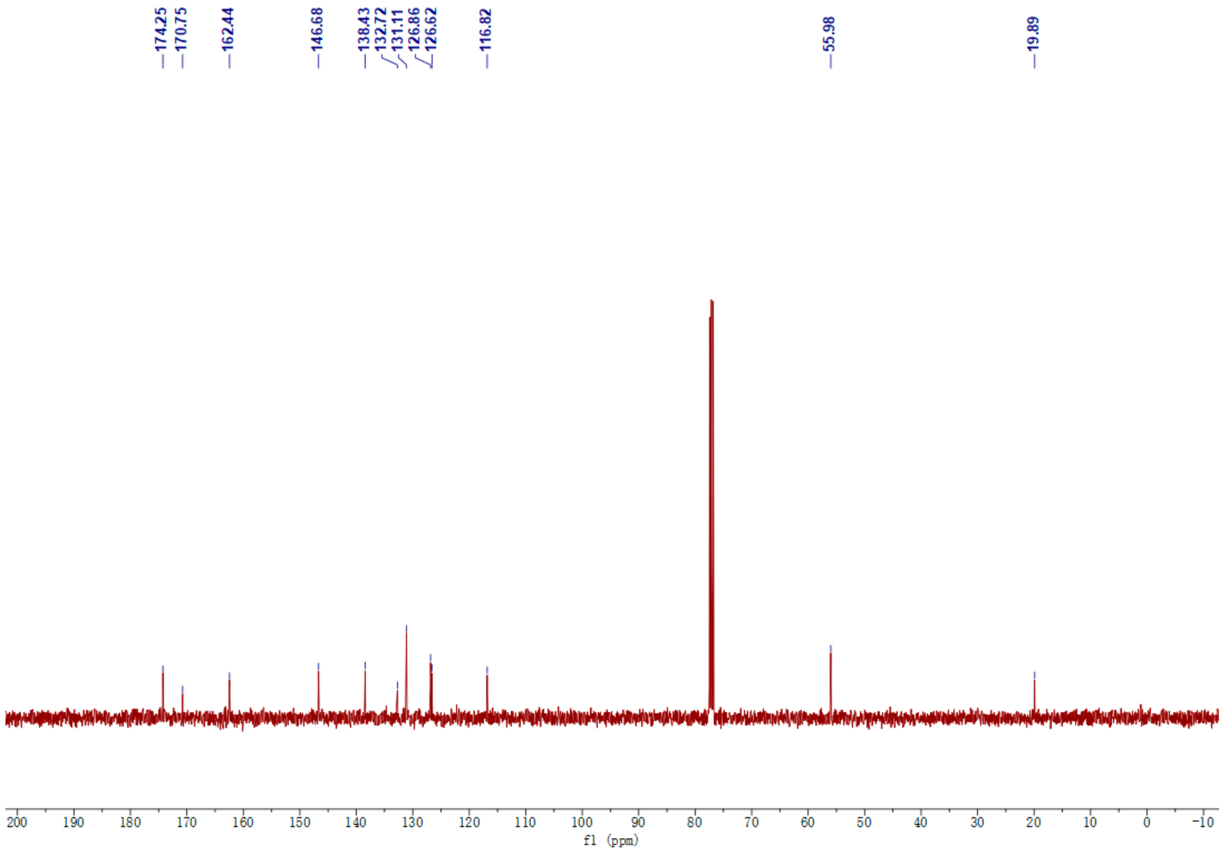

IR of **3ia**

IR spectra data: **IR(KBr)**: ν 3059, 3027, 2962, 2930, 2870, 2212, 2161, 1626, 1601, 1502, 1447, 1312, 1289, 1189, 1169, 1060, 986, 869, 833, 761, 694, 671, 558, 519 cm^{-1}.

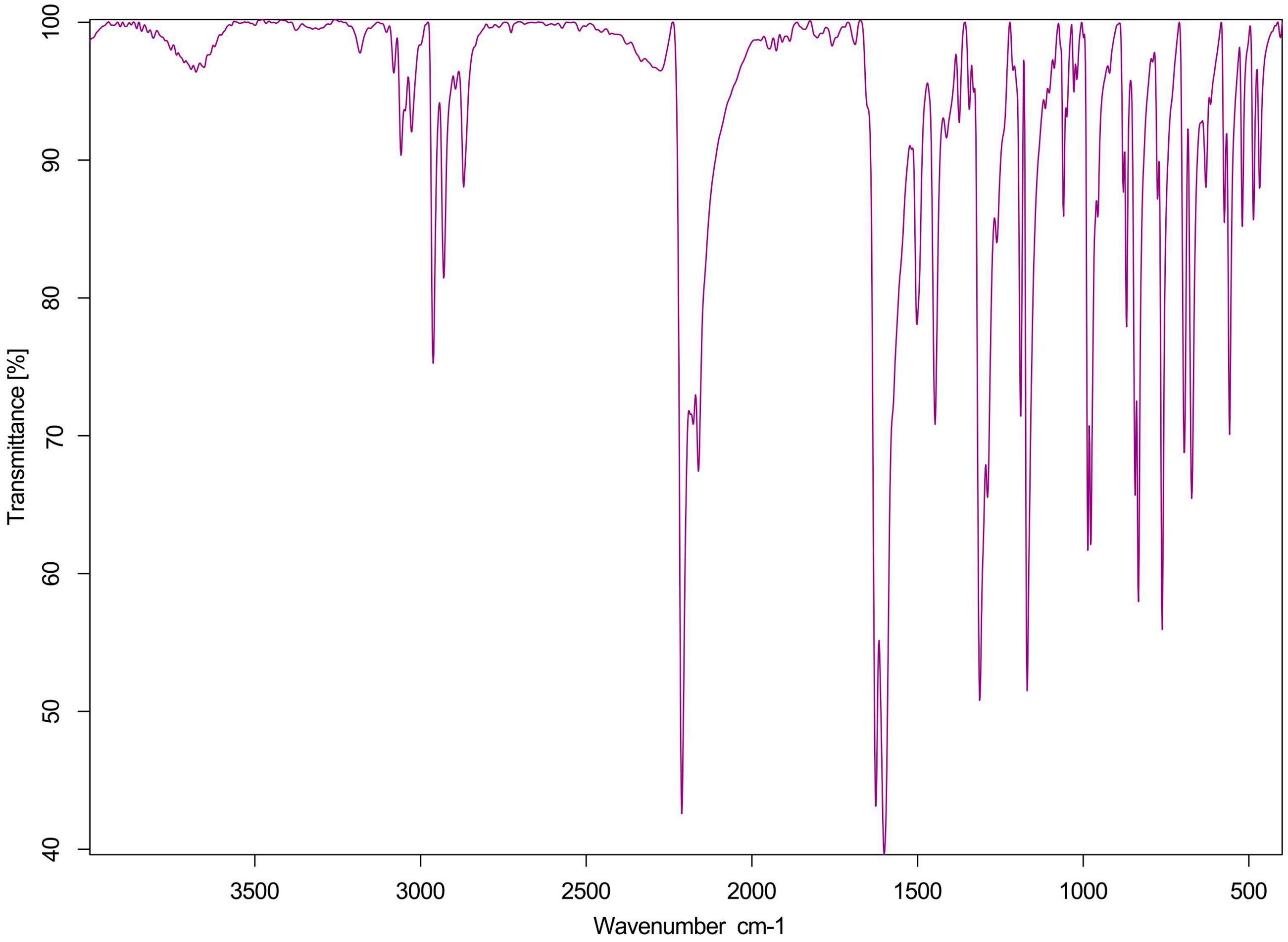

^{1}H NMR of **3ja**

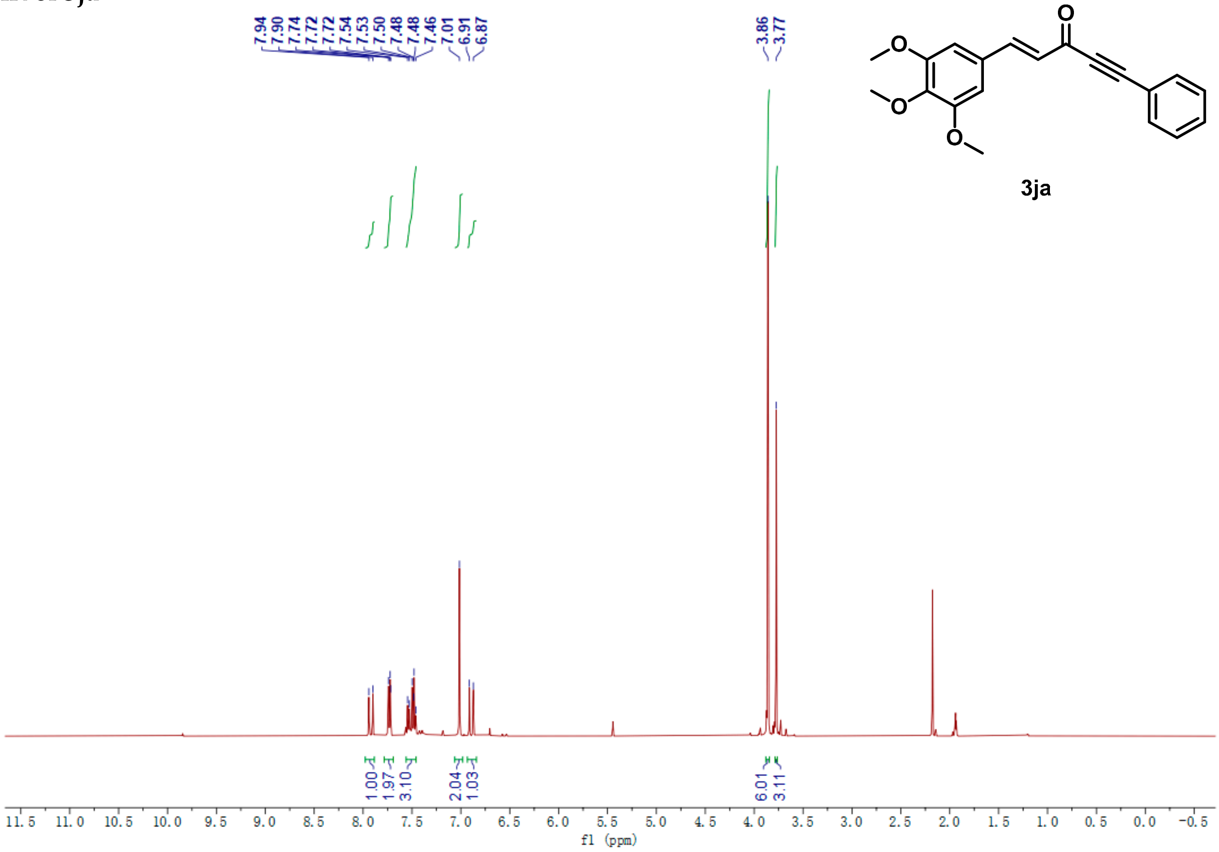

^{13}C NMR of **3ja**

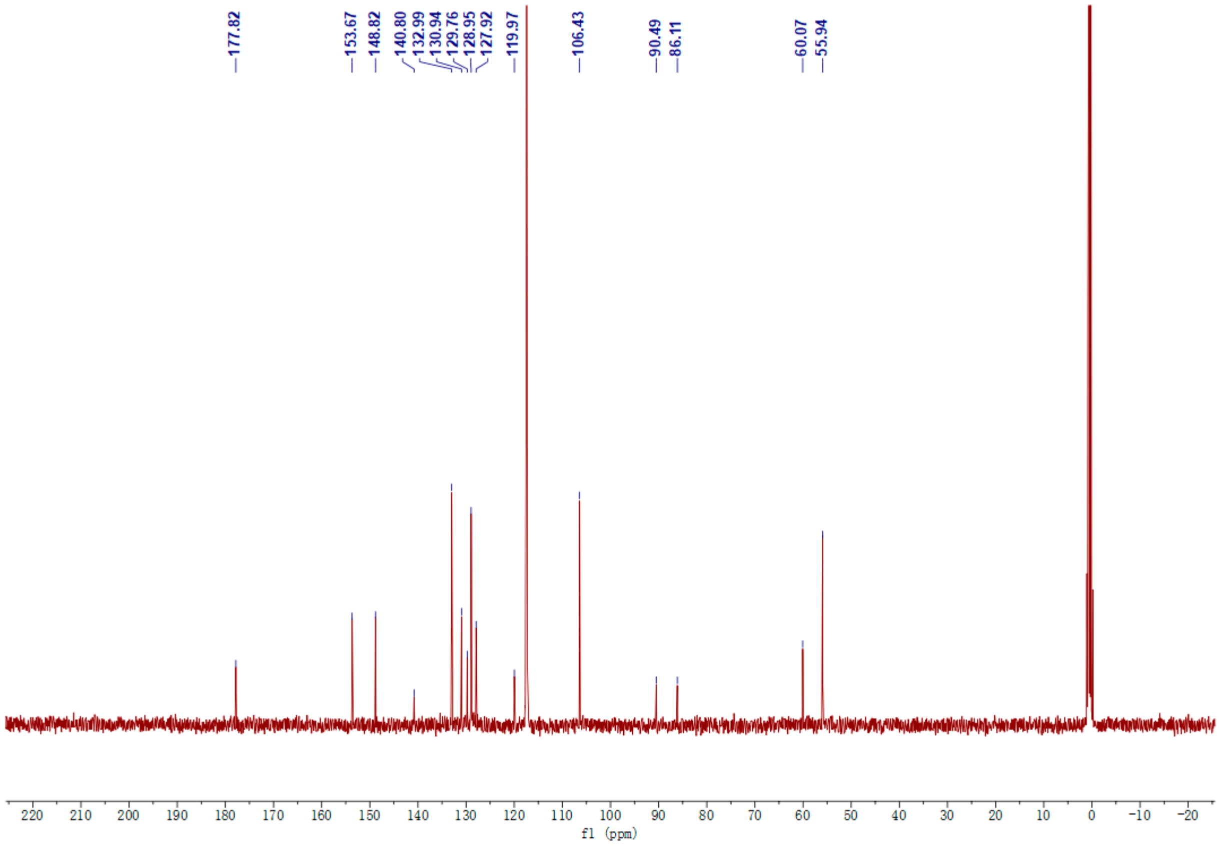

IR of **3ja**

IR spectra data: **IR(KBr)**: ν 2939, 2210, 1626, 1580, 1503, 1456, 1418, 1287, 1245, 1172, 1155, 1127, 1001, 824, 758, 689, 602, 534, 2838, 2999, 1103, 972 cm^{-1}.

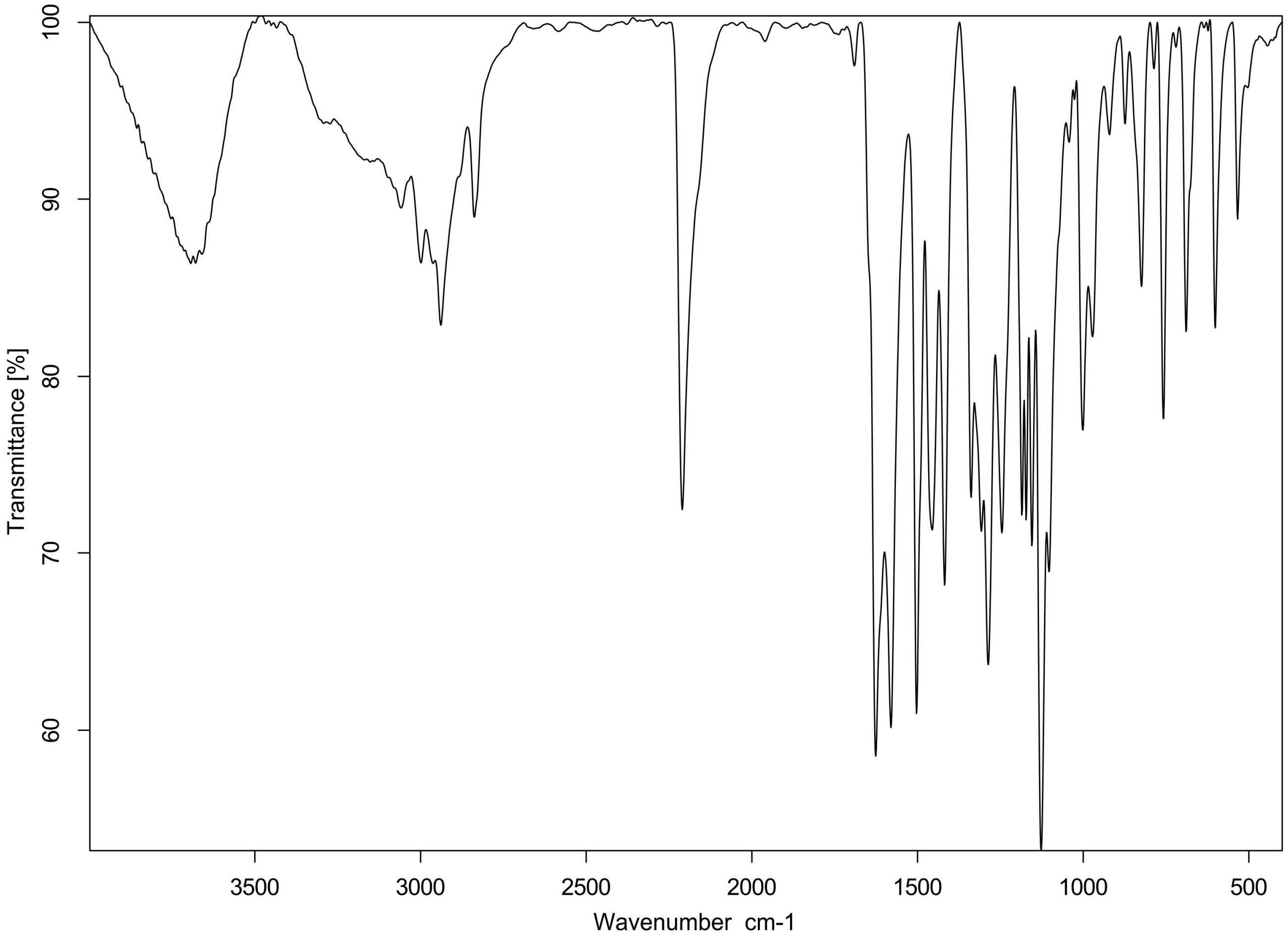

H NMR of **3ka**

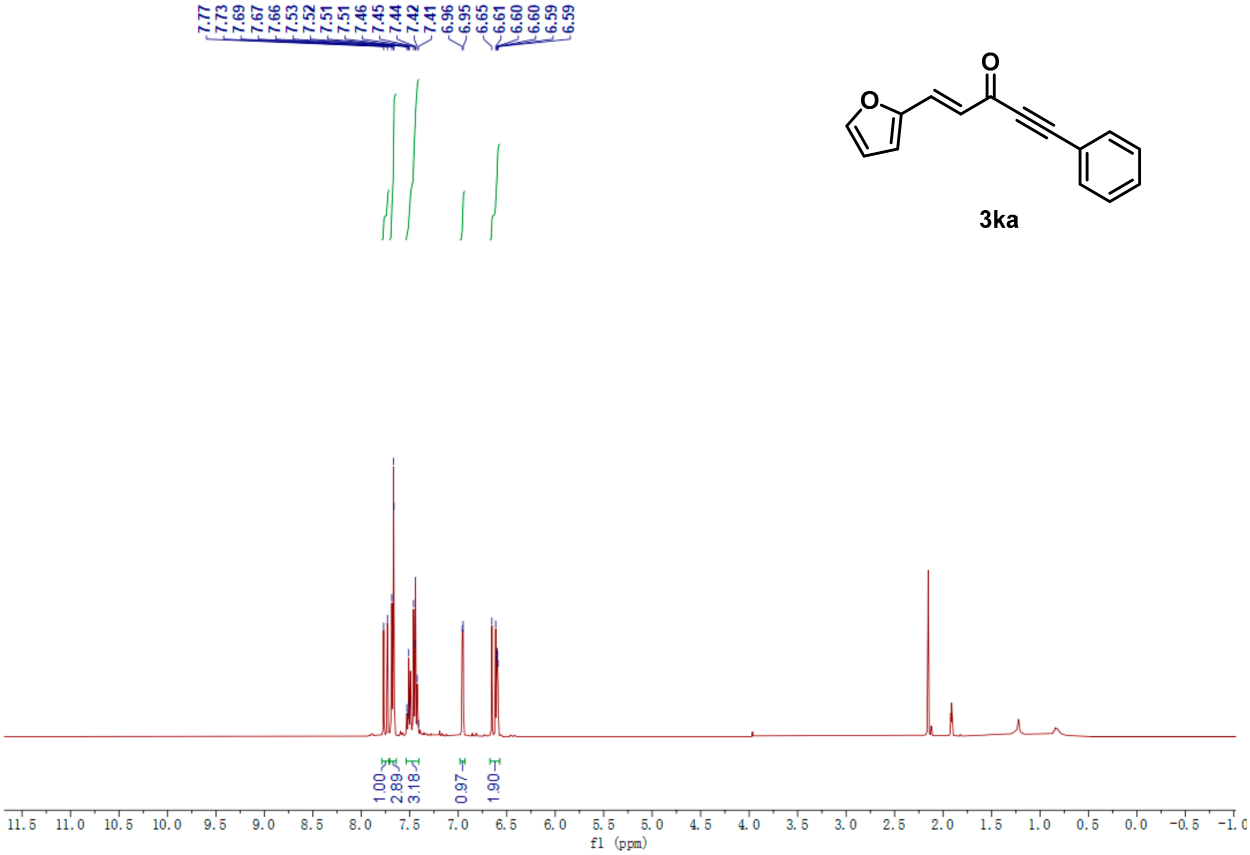

^{13}C NMR of **3ka**

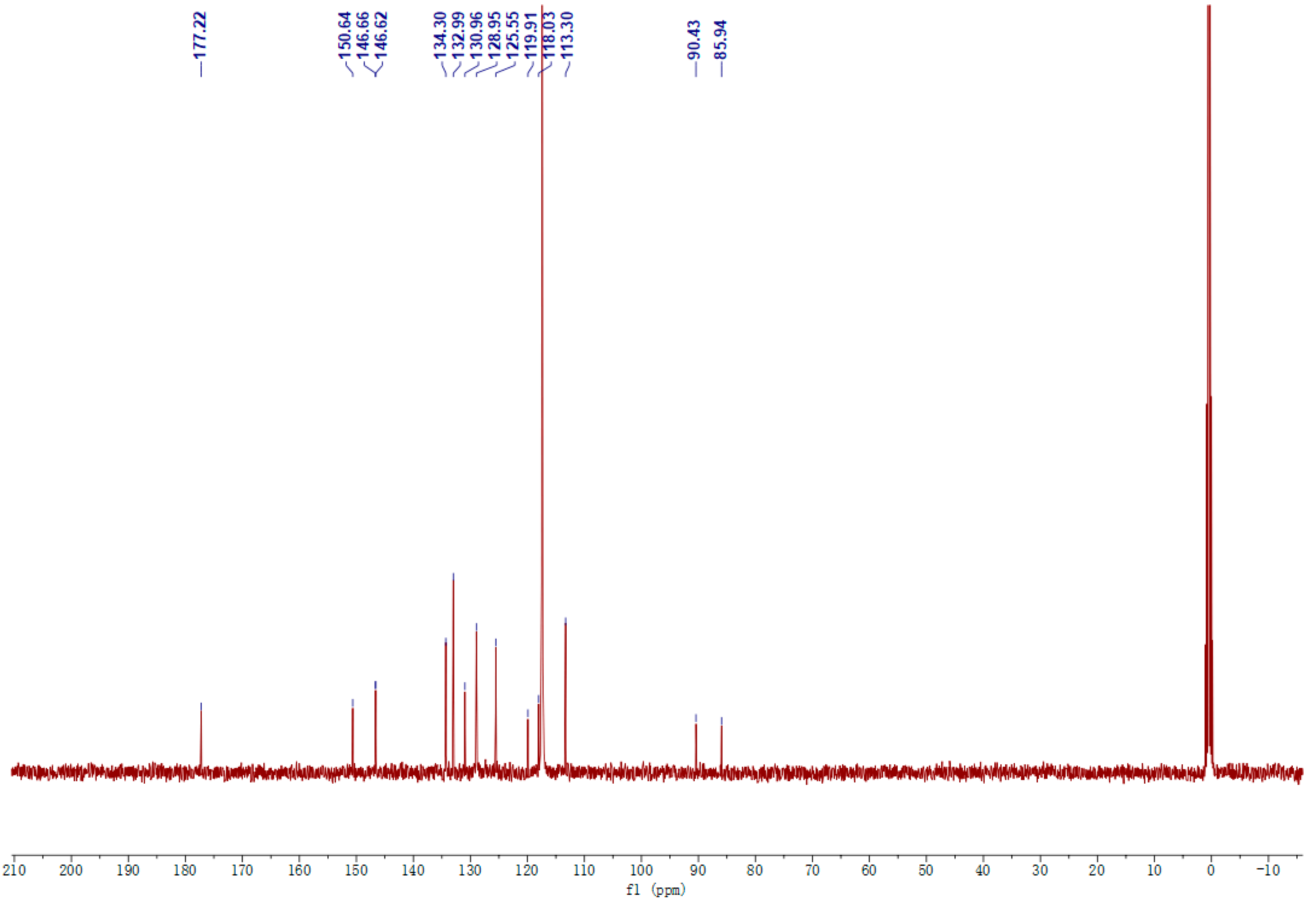

IR of **3ka**

IR spectra data: **IR(KBr)**: ν 2924, 2854, 2211, 1622, 1551, 1489, 1389, 1304, 1279, 1170, 1104, 1073, 1019, 966, 930, 883, 757, 690, 615, 592, 535 cm^{-1}.

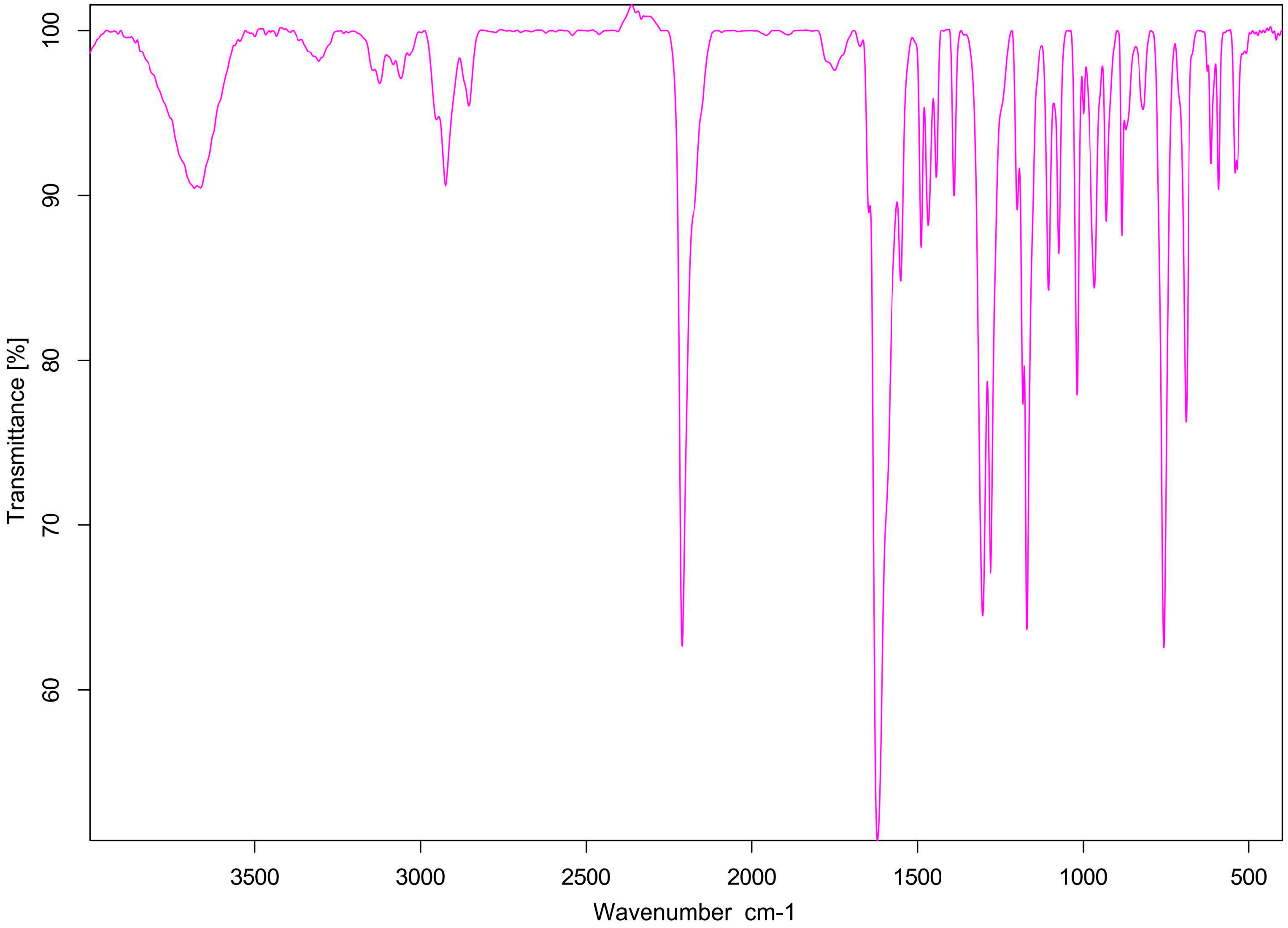

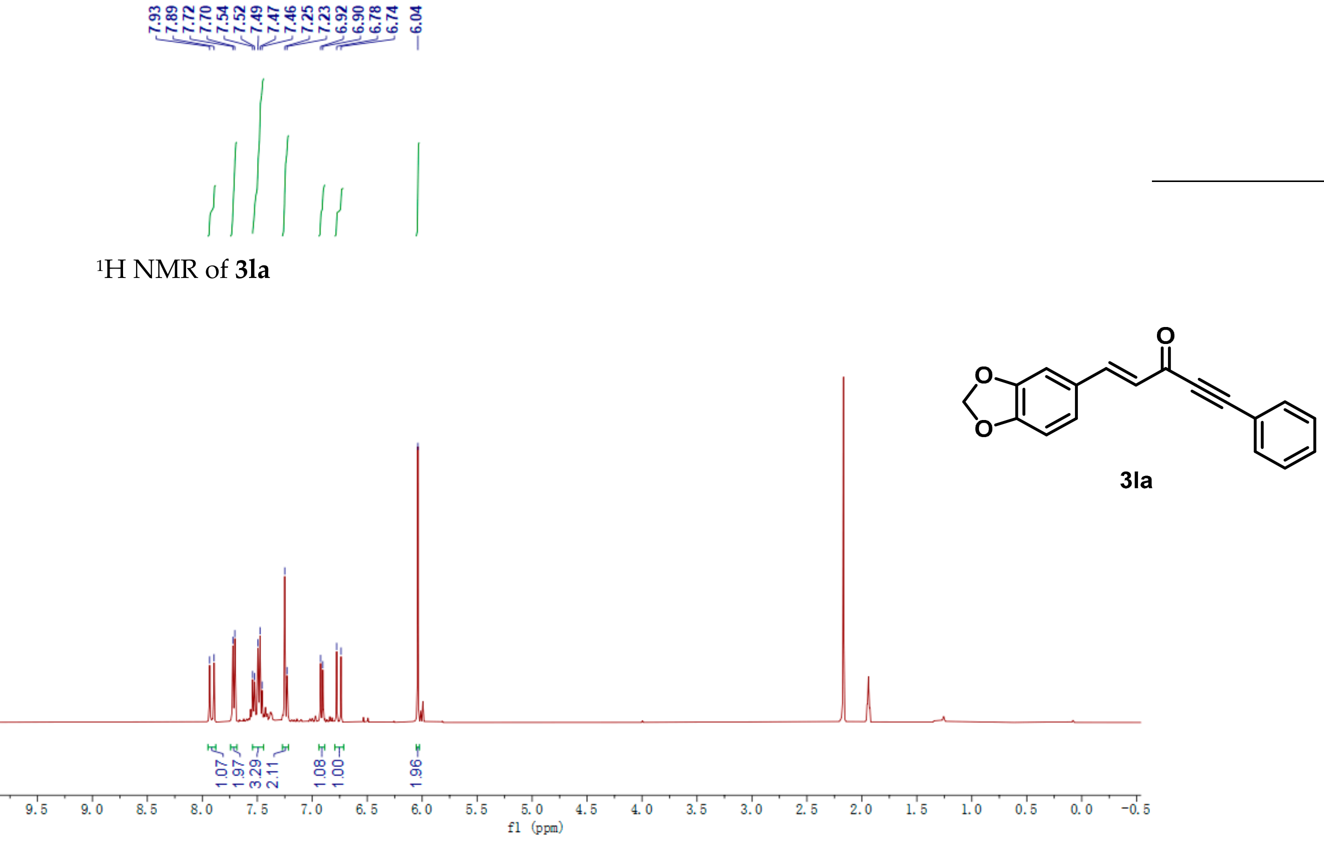

^{1}H NMR of **3la**

3la

^{13}C NMR of **3la**

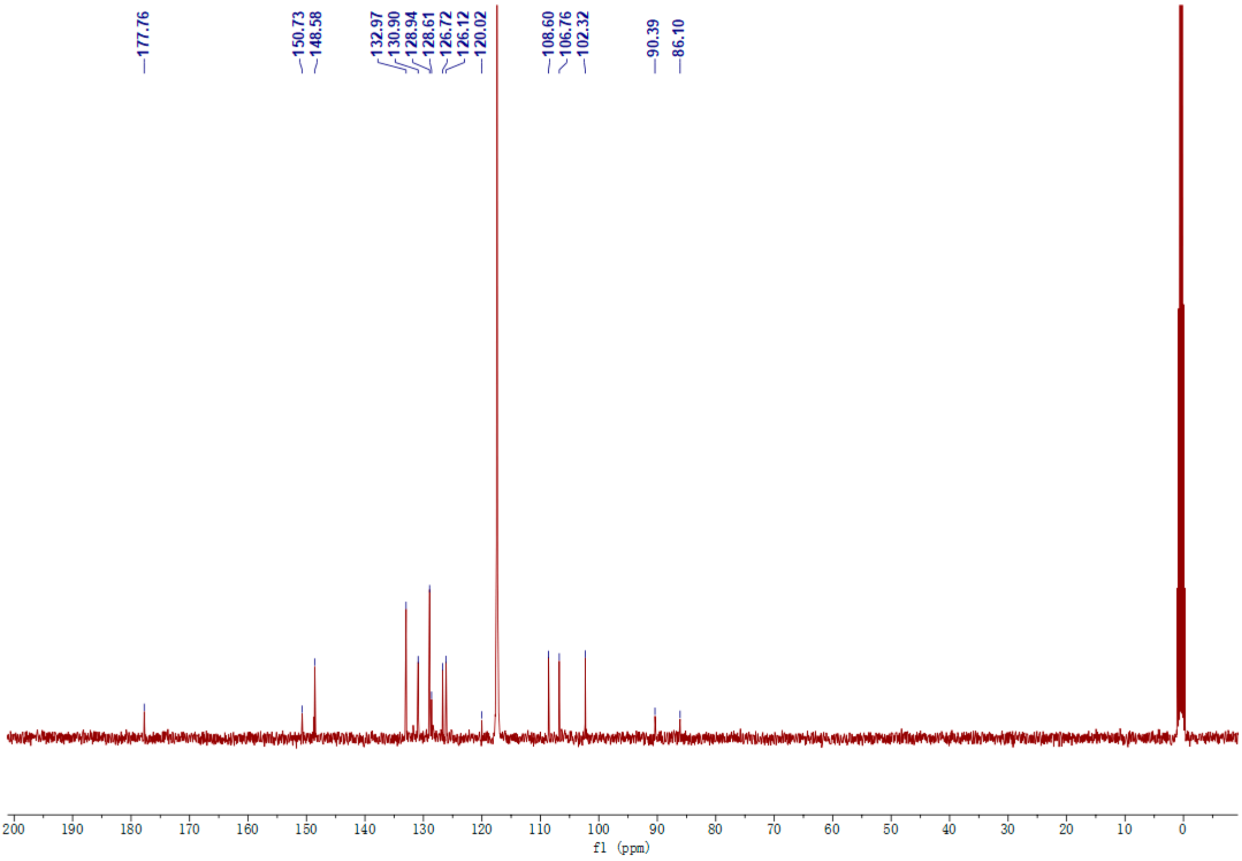

IR of **3la**

IR spectra data: **IR(KBr)**: ν 3070, 3008, 2913, 2788, 2210, 2164, 1624, 1599, 1500, 1447, 1302, 1260, 1171, 1105, 1037, 969, 923, 859, 816, 761, 688, 593, 543 cm^{-1}.

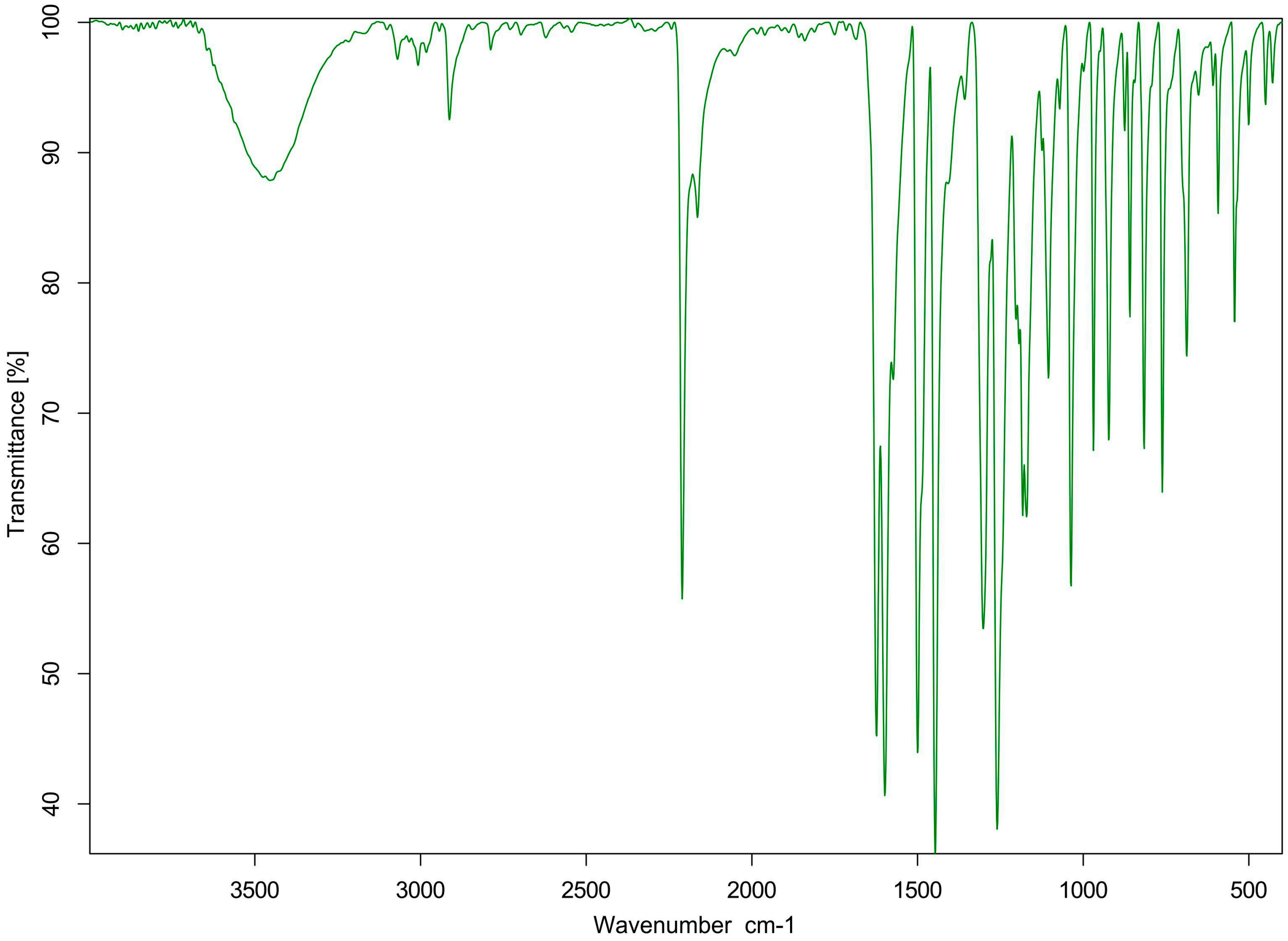

^{1}H NMR of **3ma**

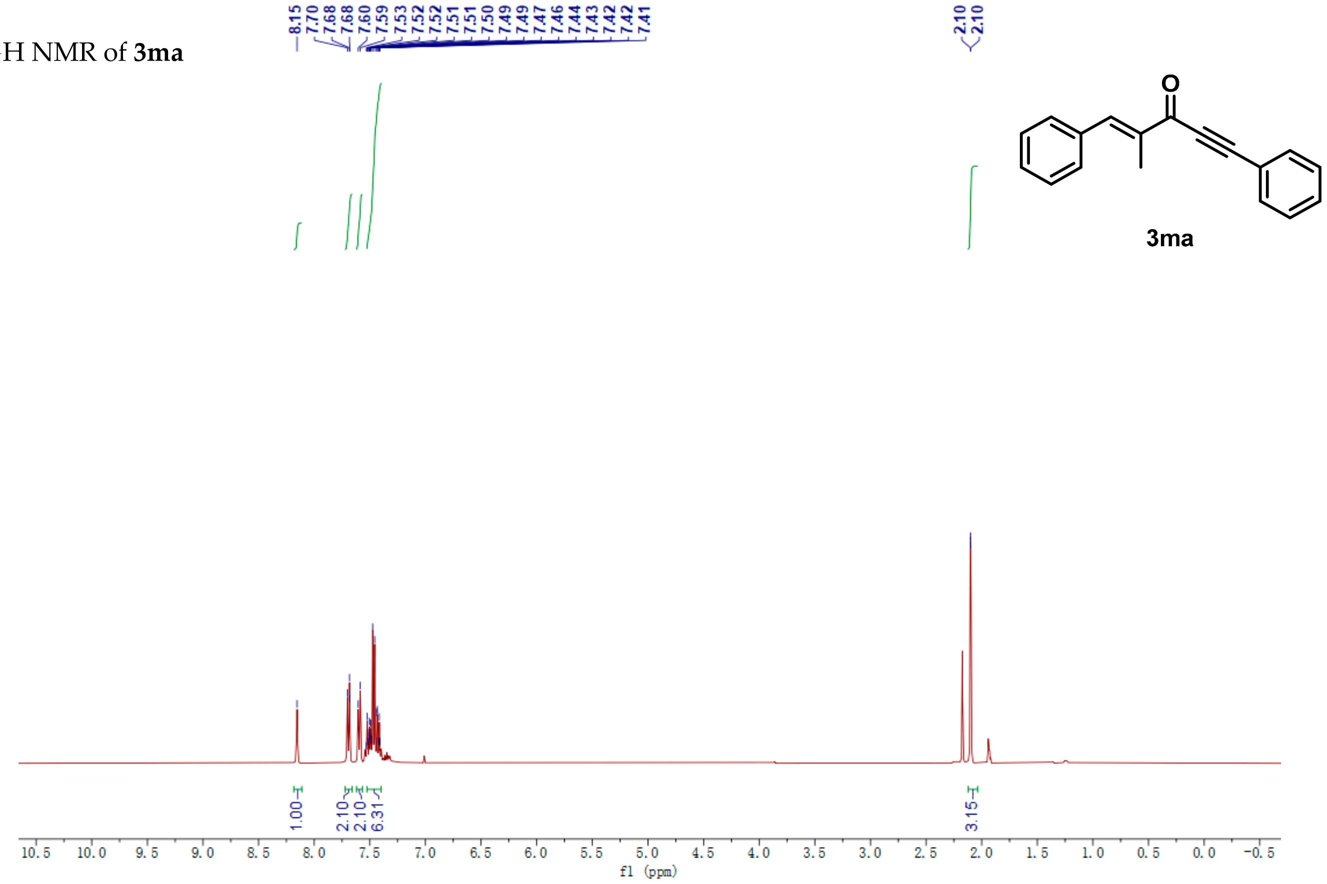

^{13}C NMR of **3ma**

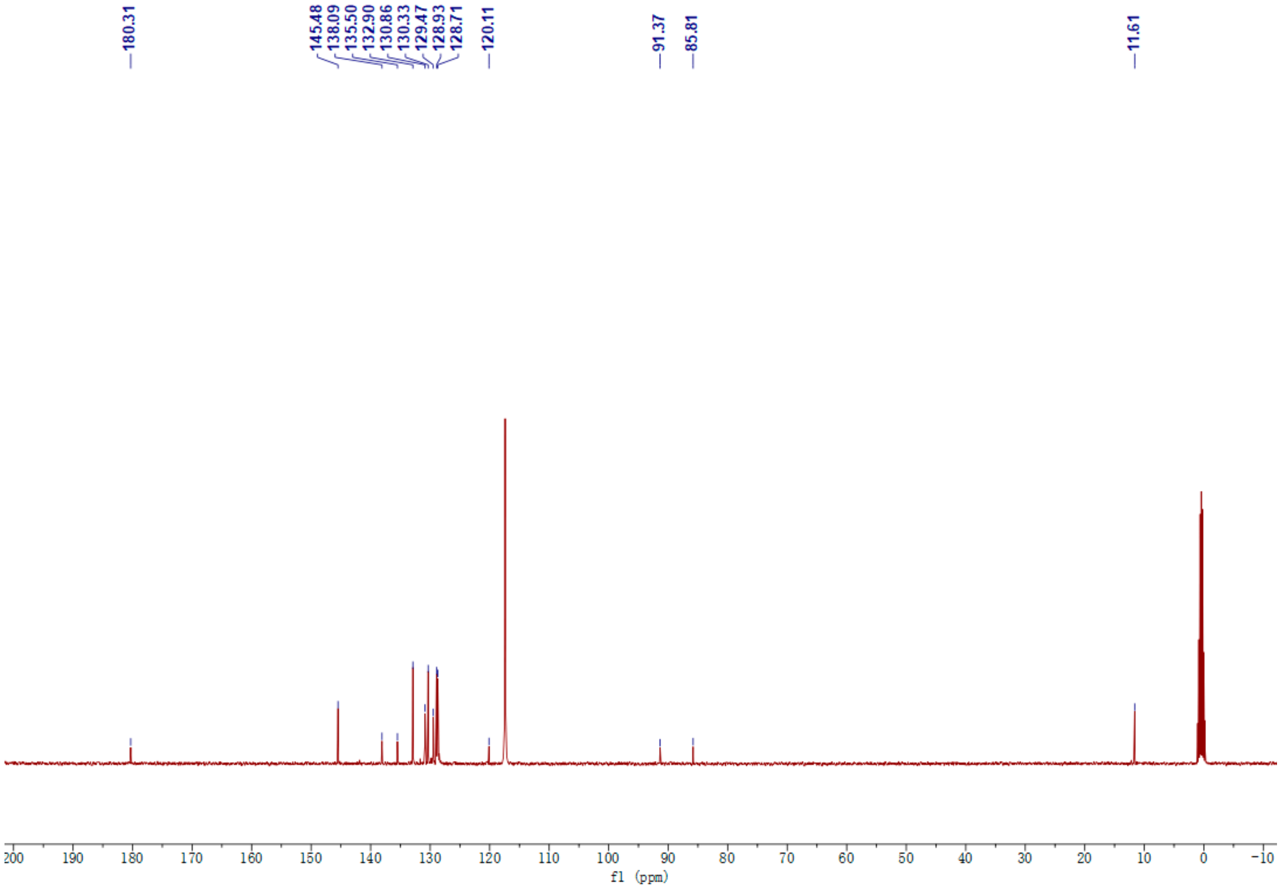

^{1}H NMR of **3na**

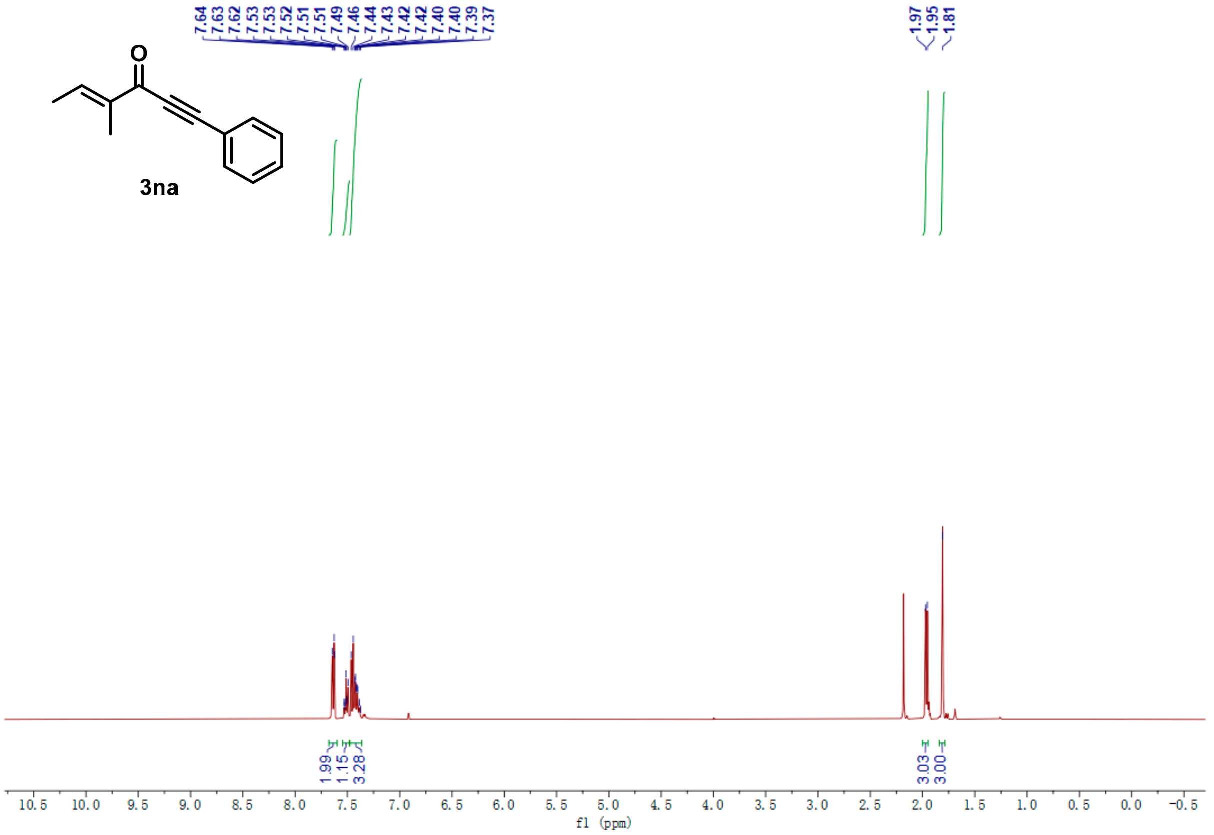

^{13}C NMR of **3na**

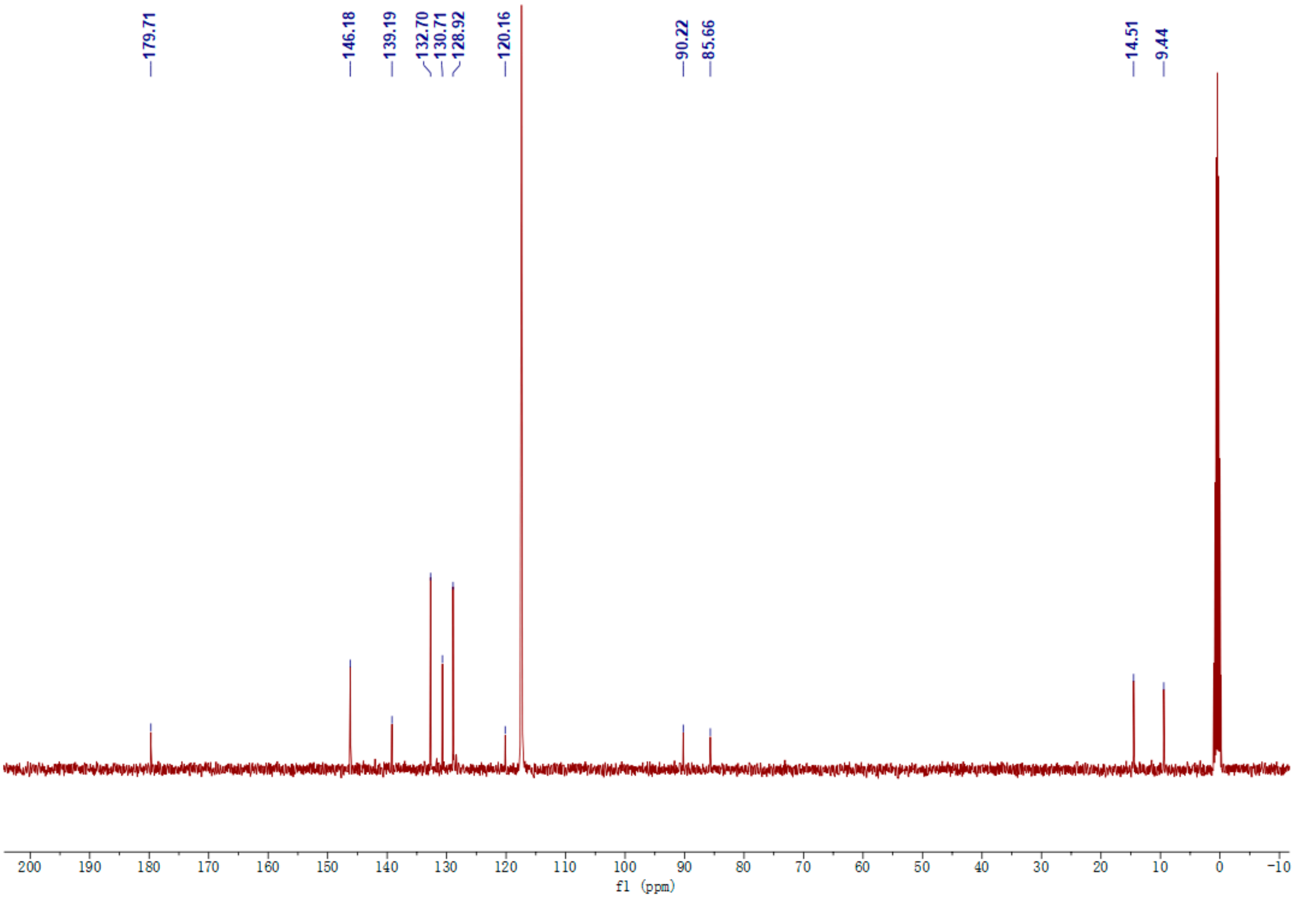

^{1}H NMR of **3oa**

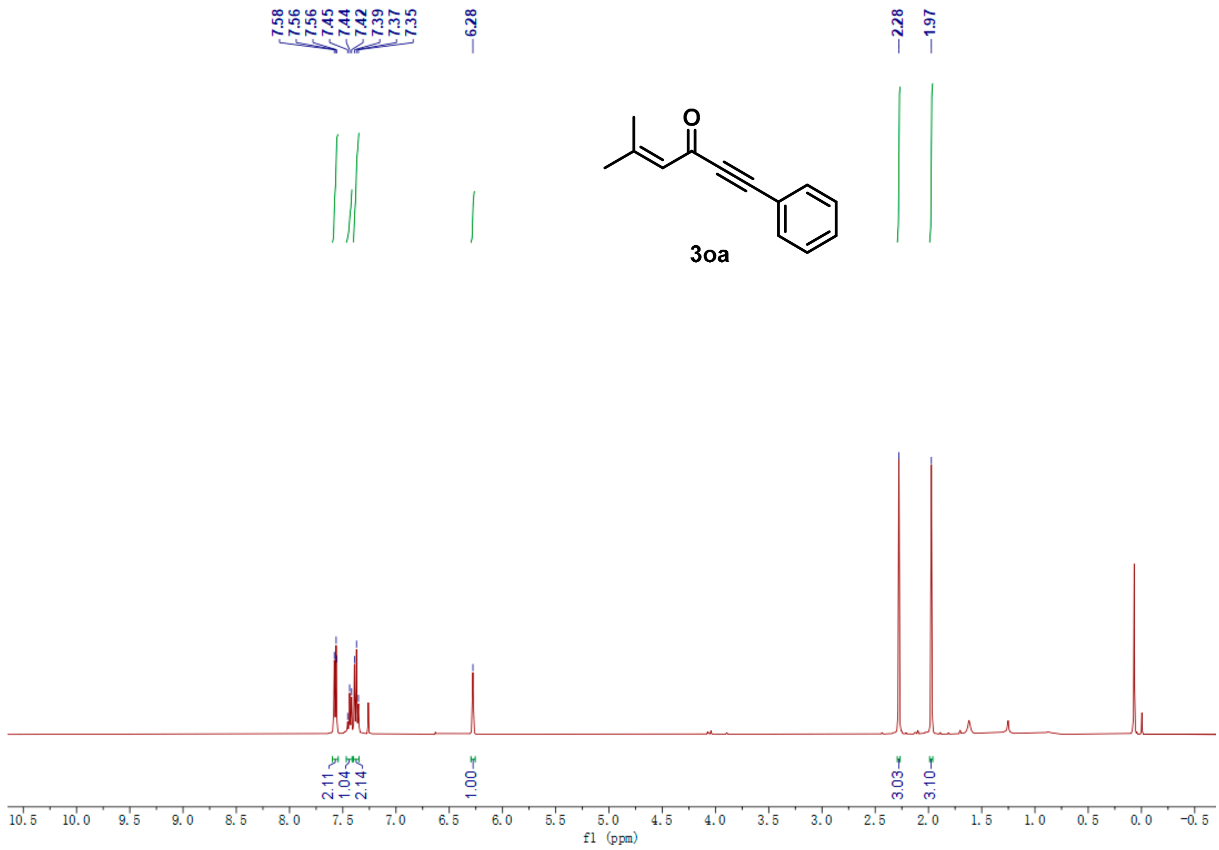

^{13}C NMR of **3oa**

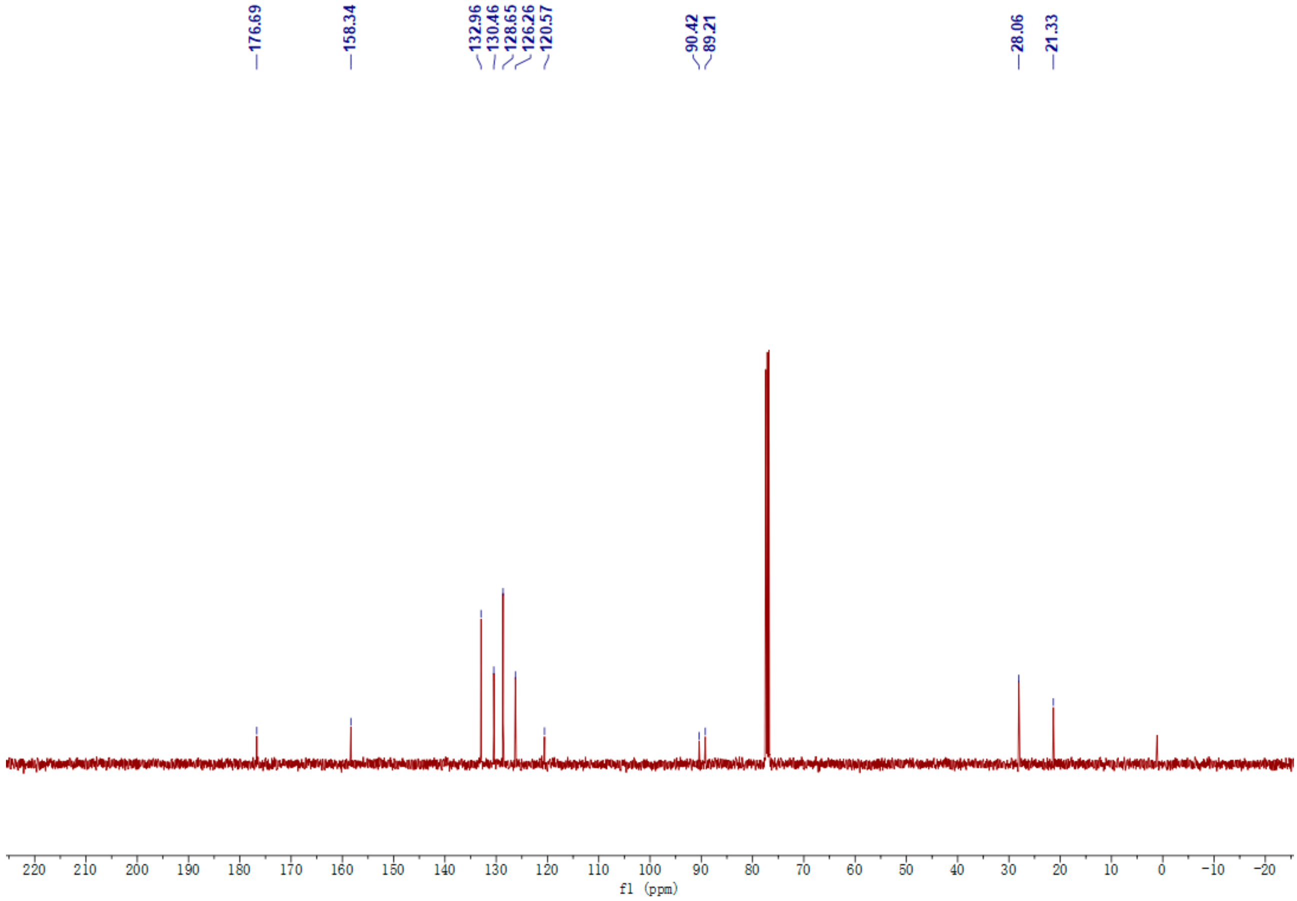

^{1}H NMR of **3pa**

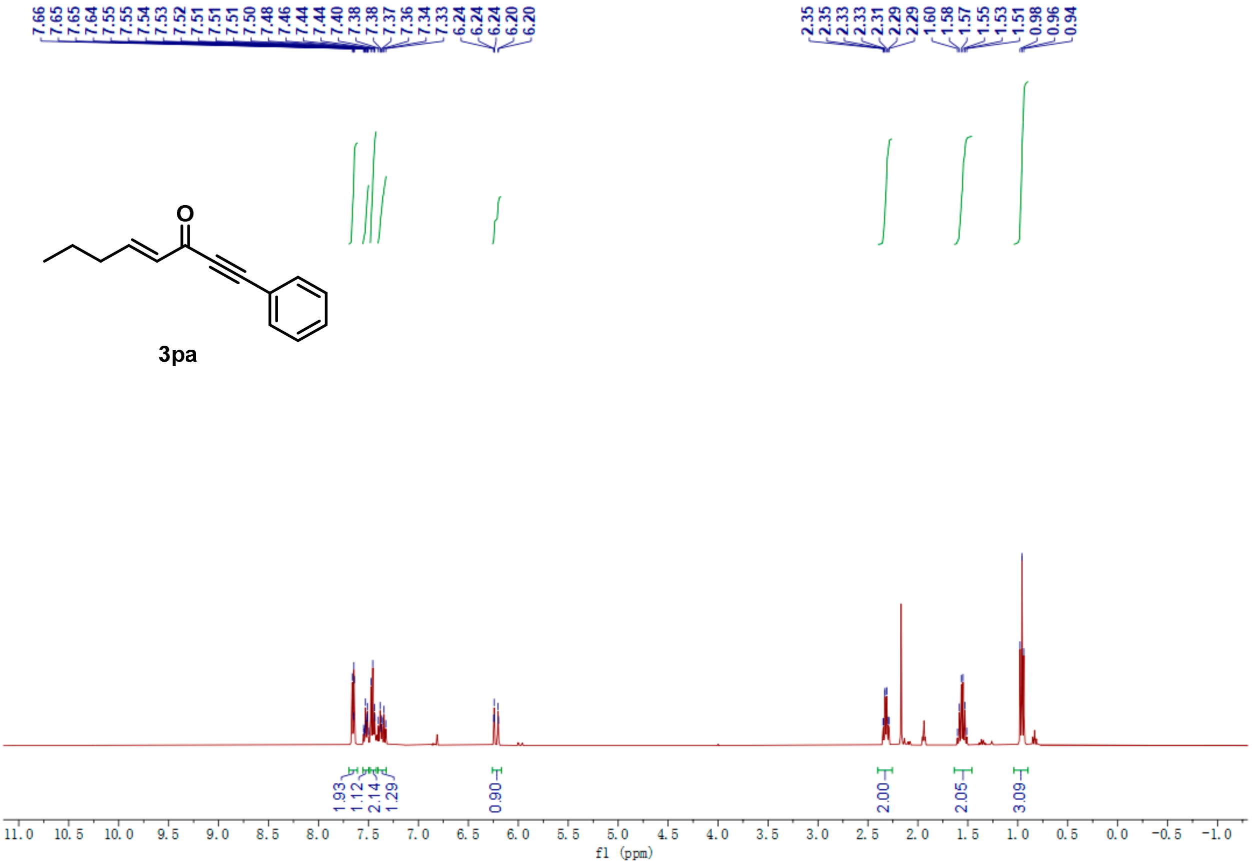

^{13}C NMR of **3pa**

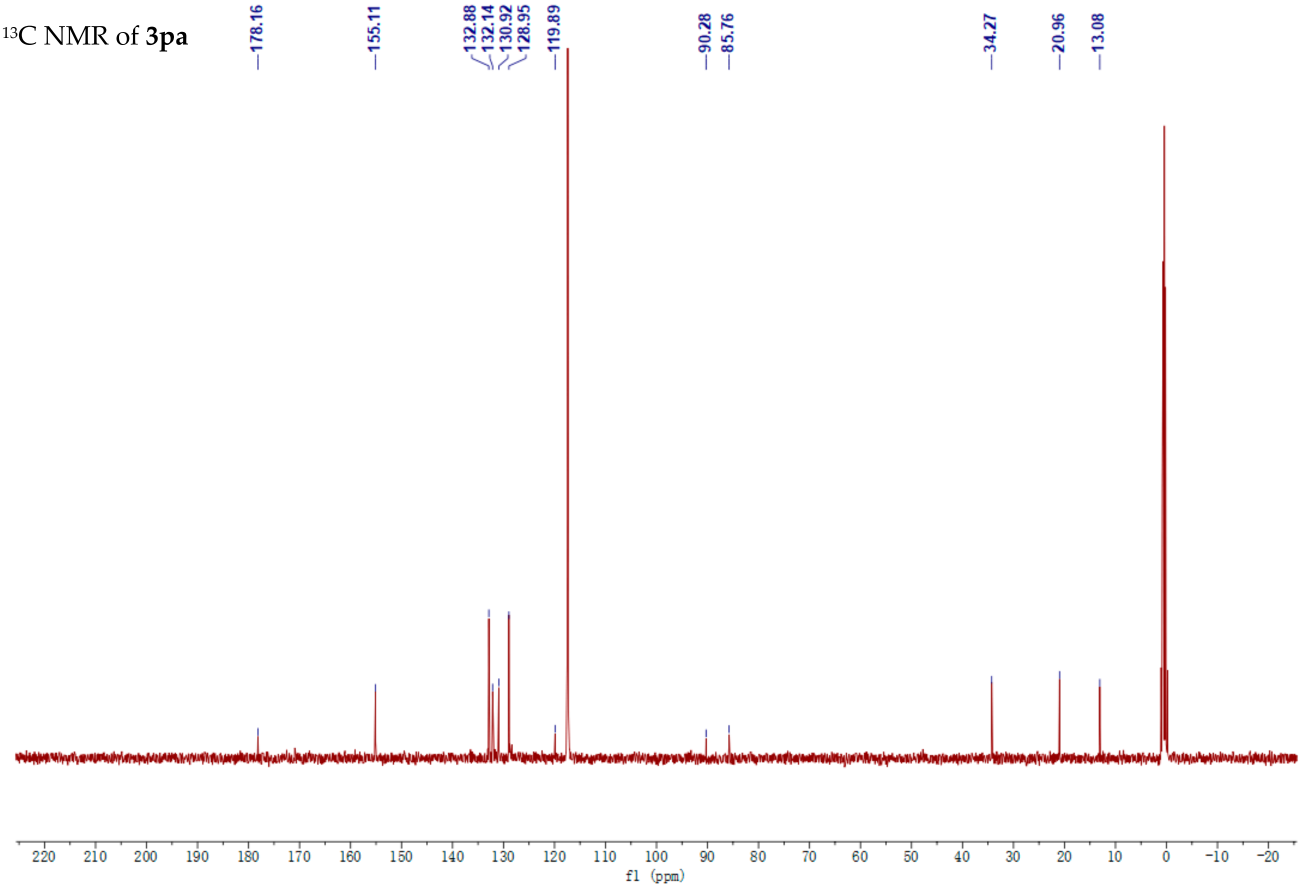

^{1}H NMR of **3qa**

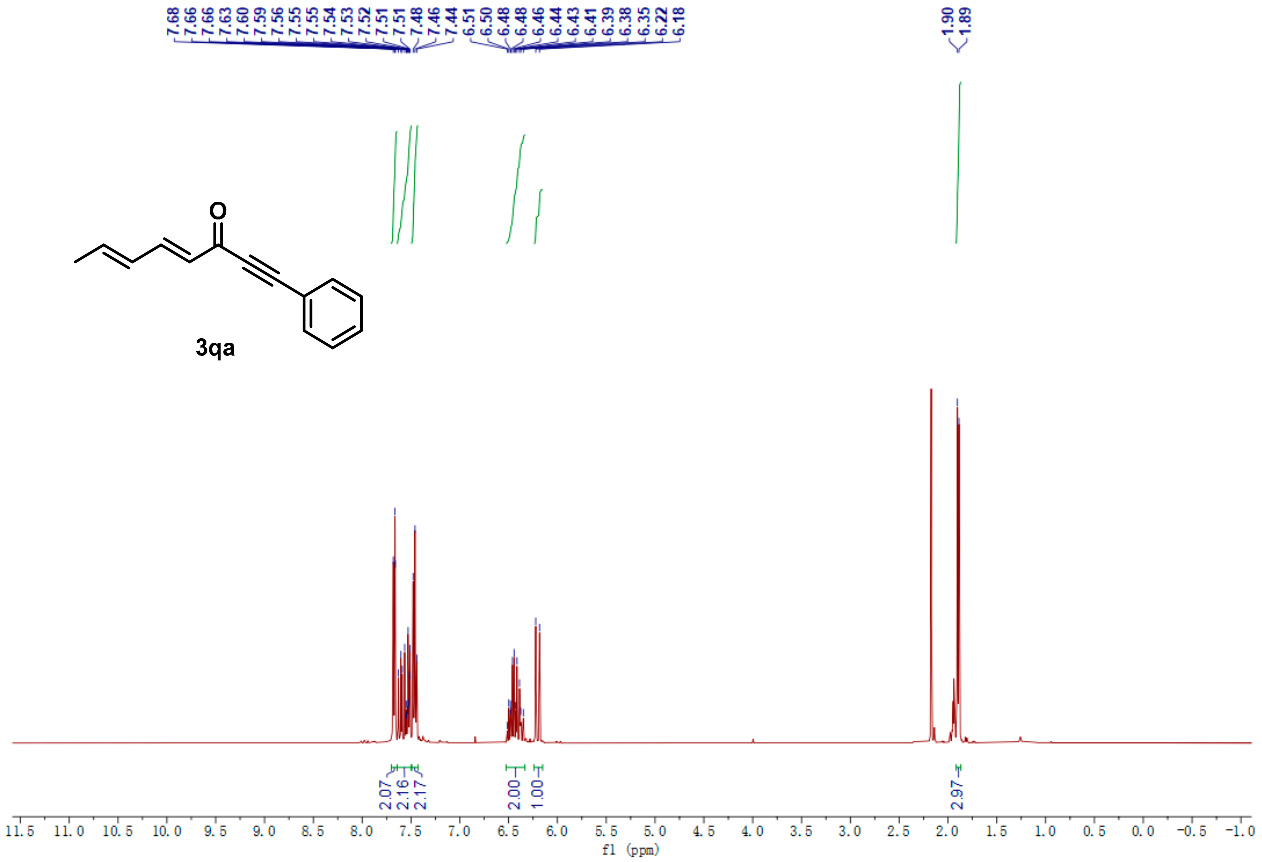

^{13}C NMR of **3qa**

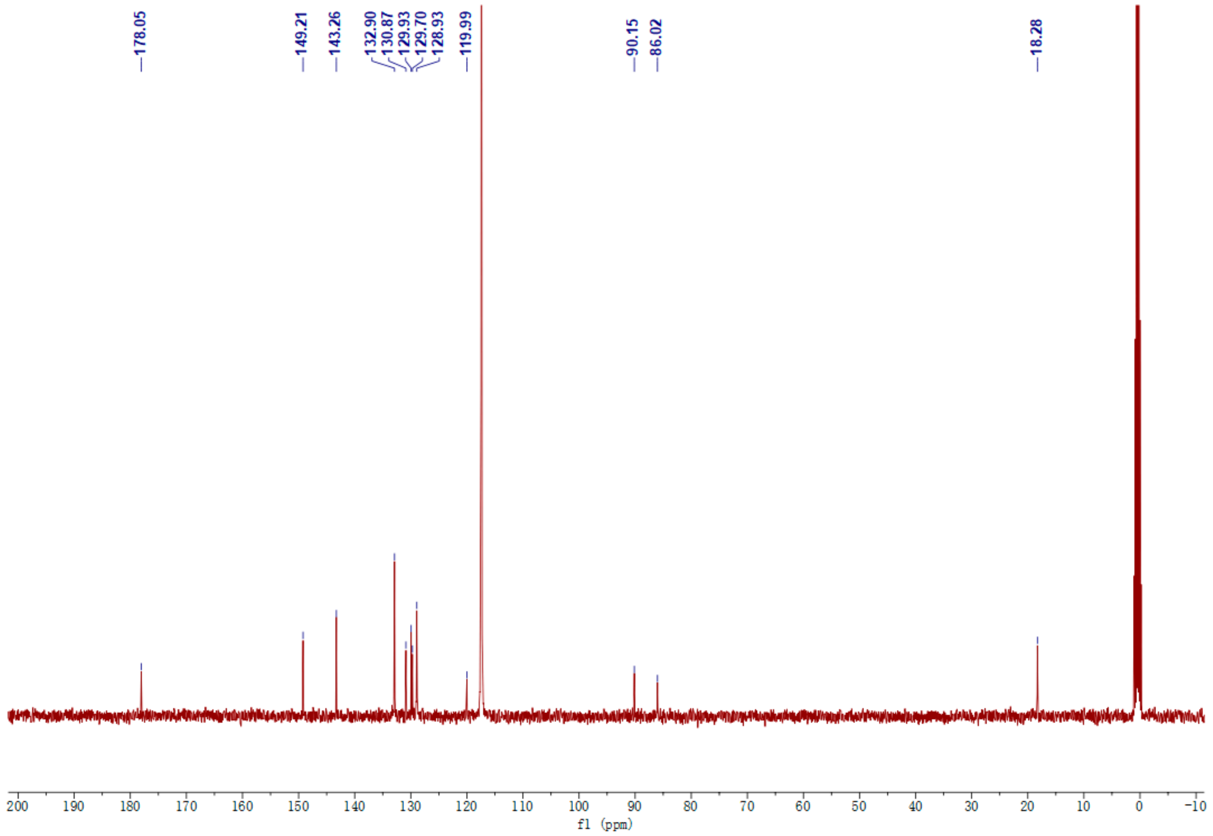

IR of **3qa**

IR spectra data: **IR(KBr)**: ν 3027, 2934, 2911, 2210, 1619, 1590, 1489, 1443, 1305, 1279, 1187, 1174, 1174, 1103, 1077, 996, 928, 928, 872, 872, 758, 690, 574, 534 cm^{-1}.

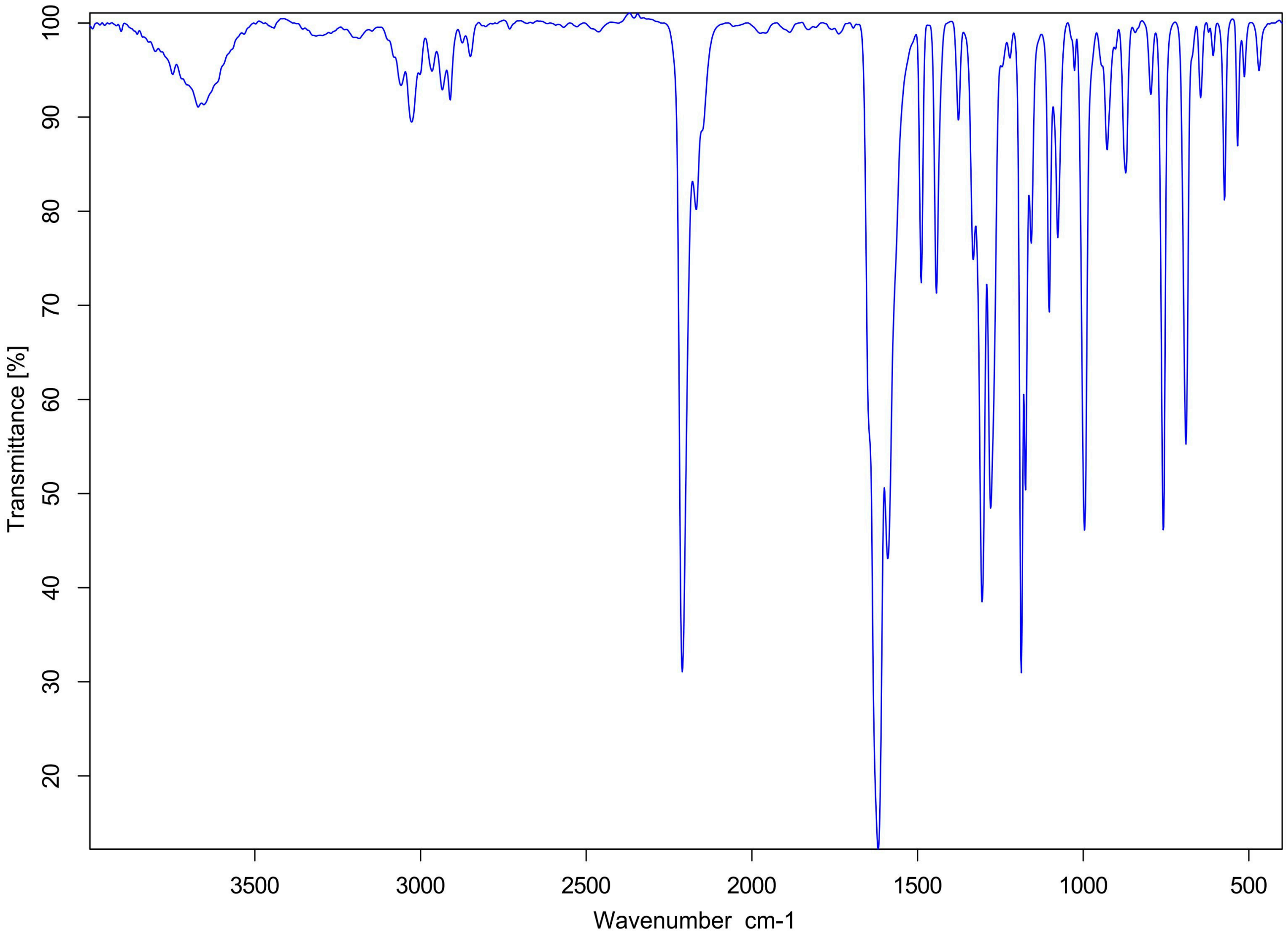

^{1}H NMR of **3ra**

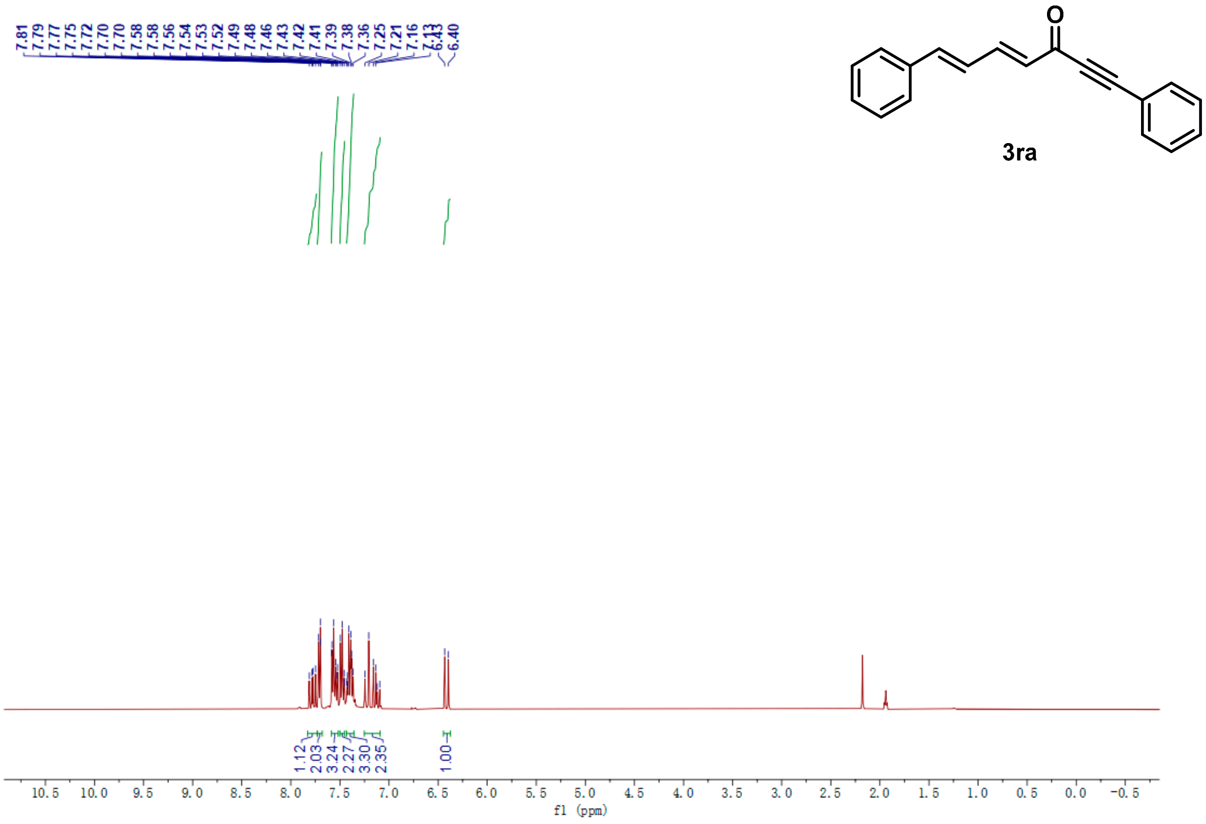
3ra

^{13}C NMR of **3ra**

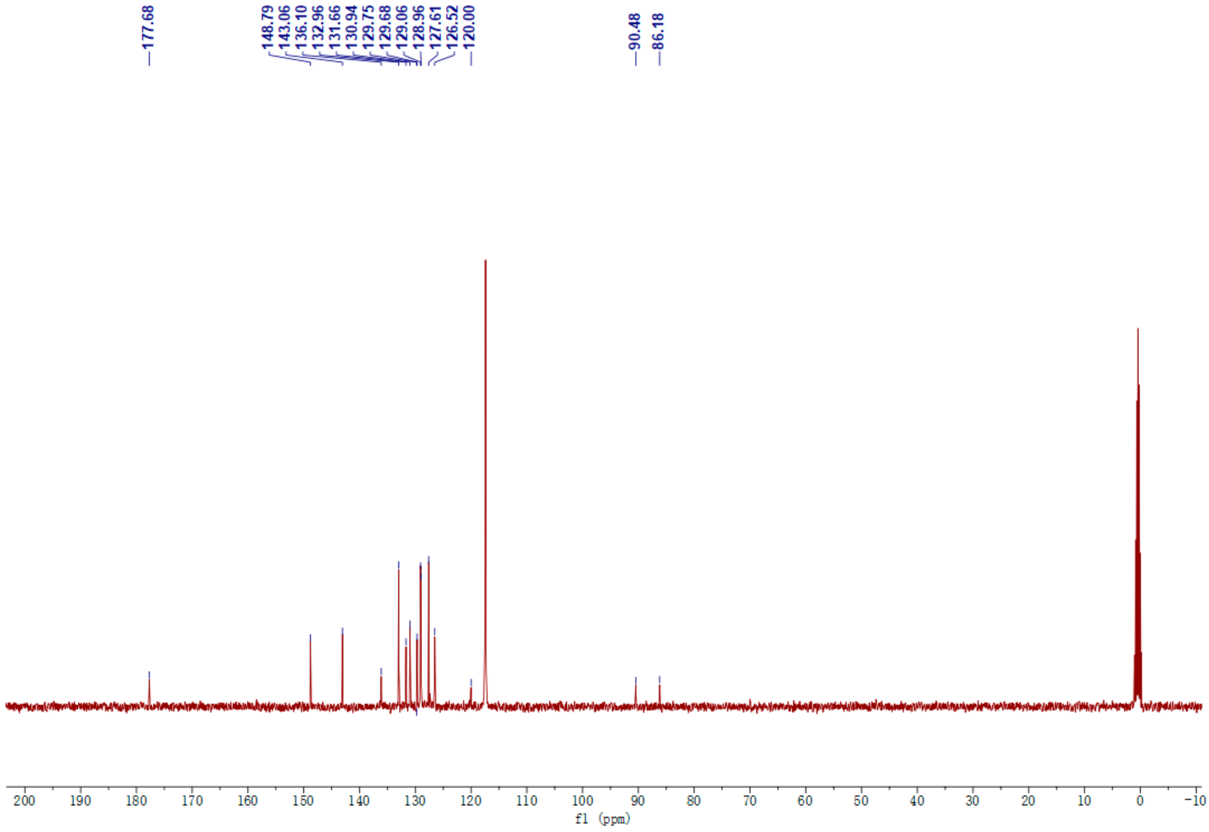

^{1}H NMR of **3ab**

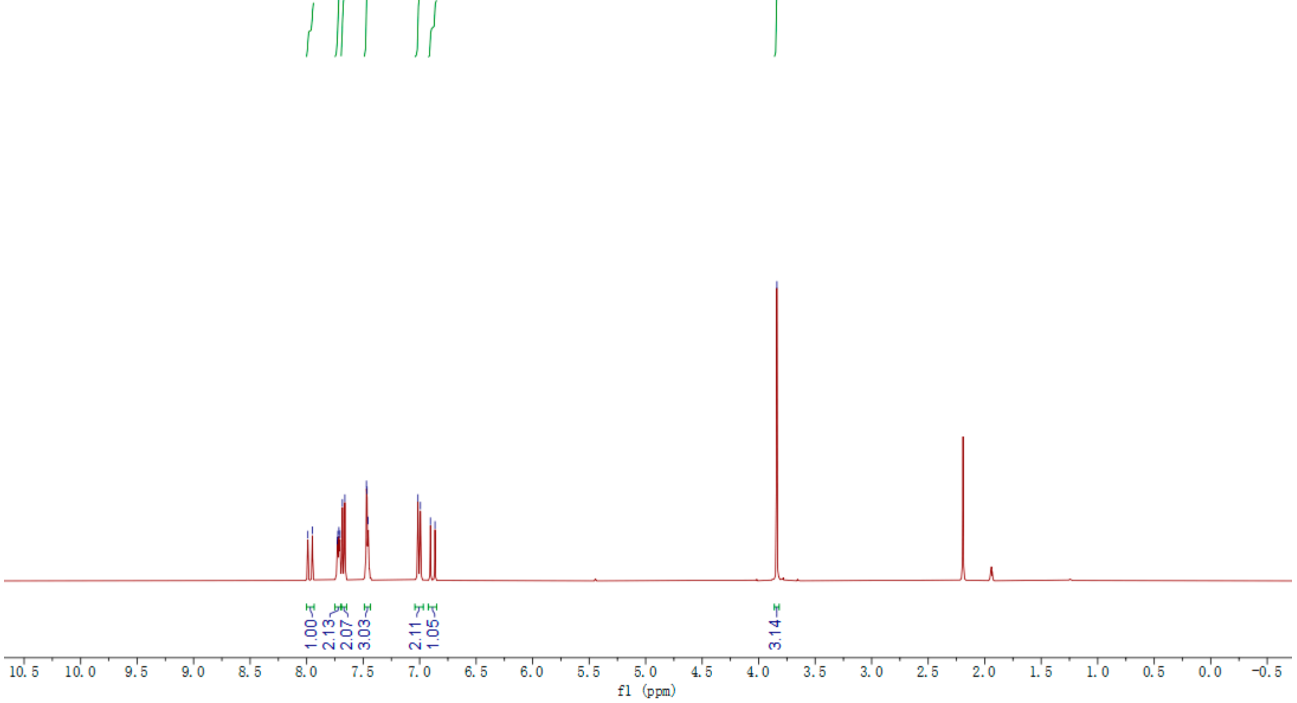

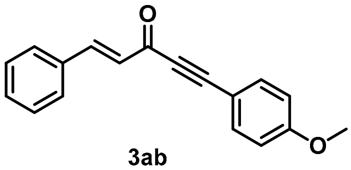

3ab

^{13}C NMR of **3ab**

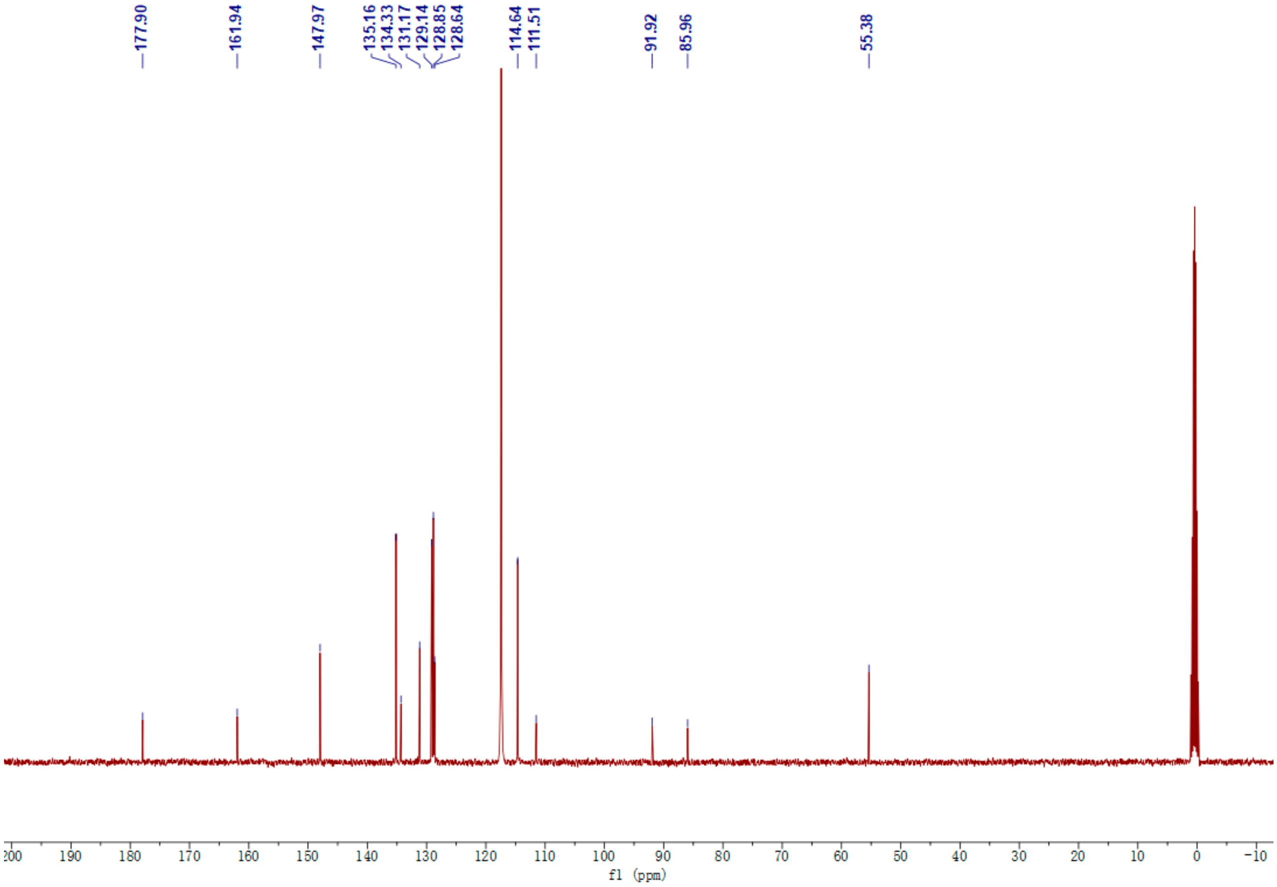

^{1}H NMR of **3ac**

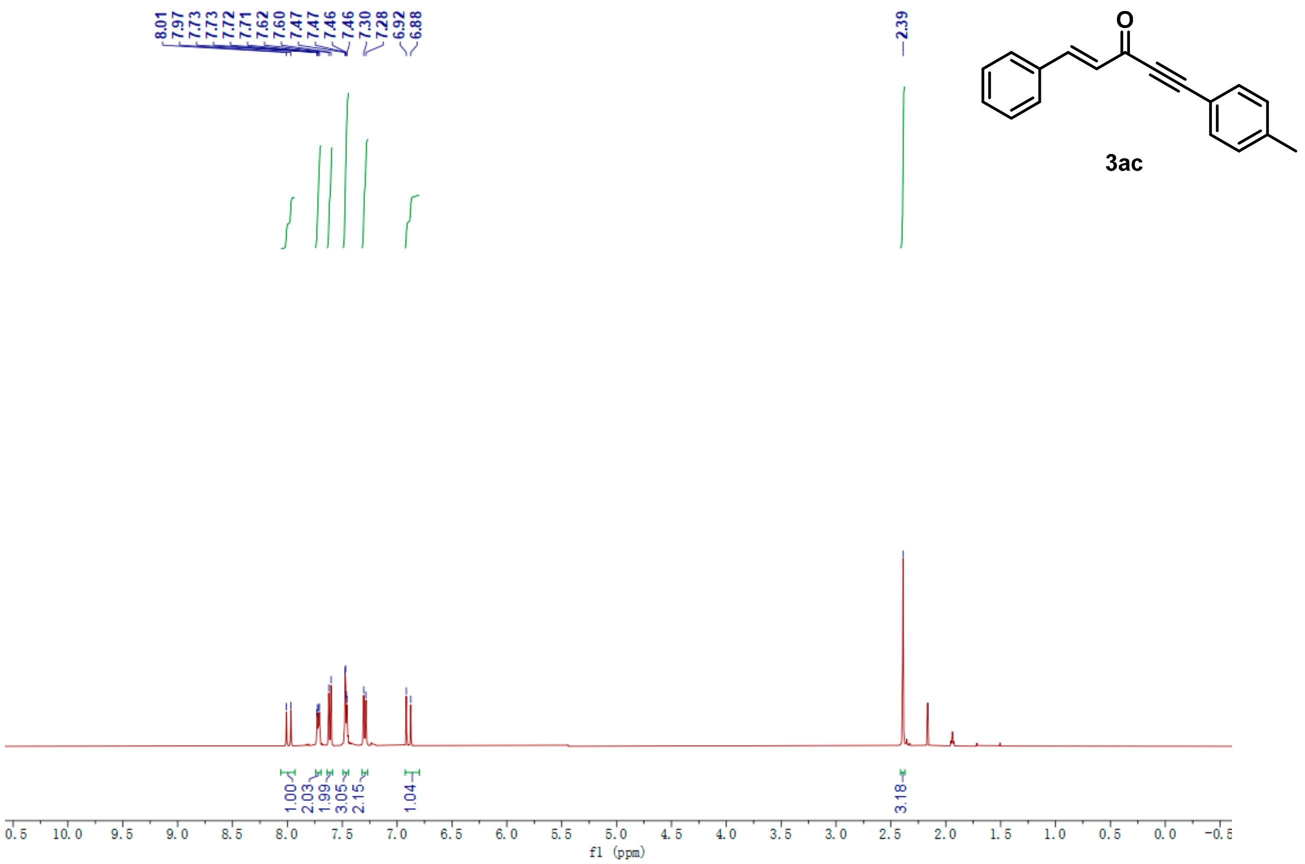

^{13}C NMR of **3ac**

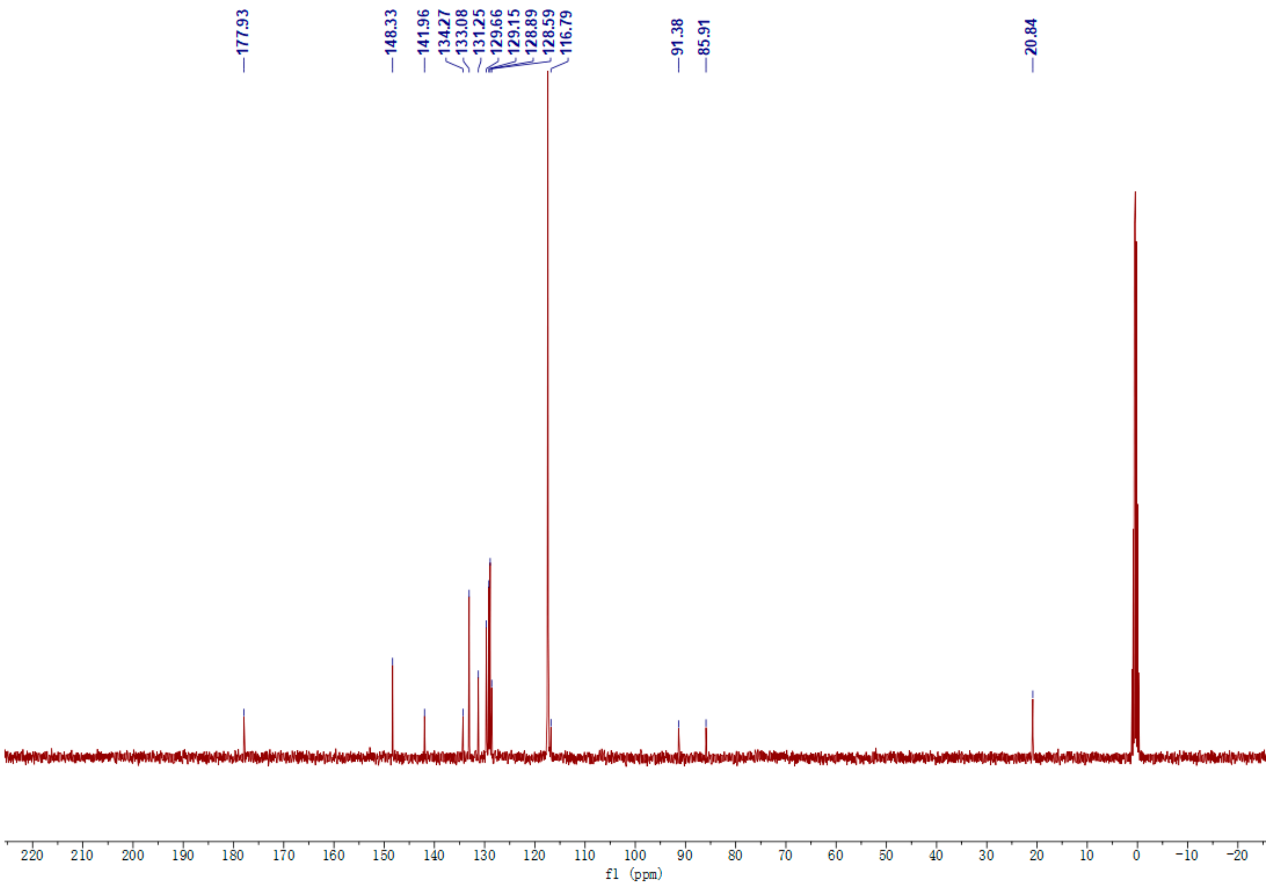

^{1}H NMR of **3ad**

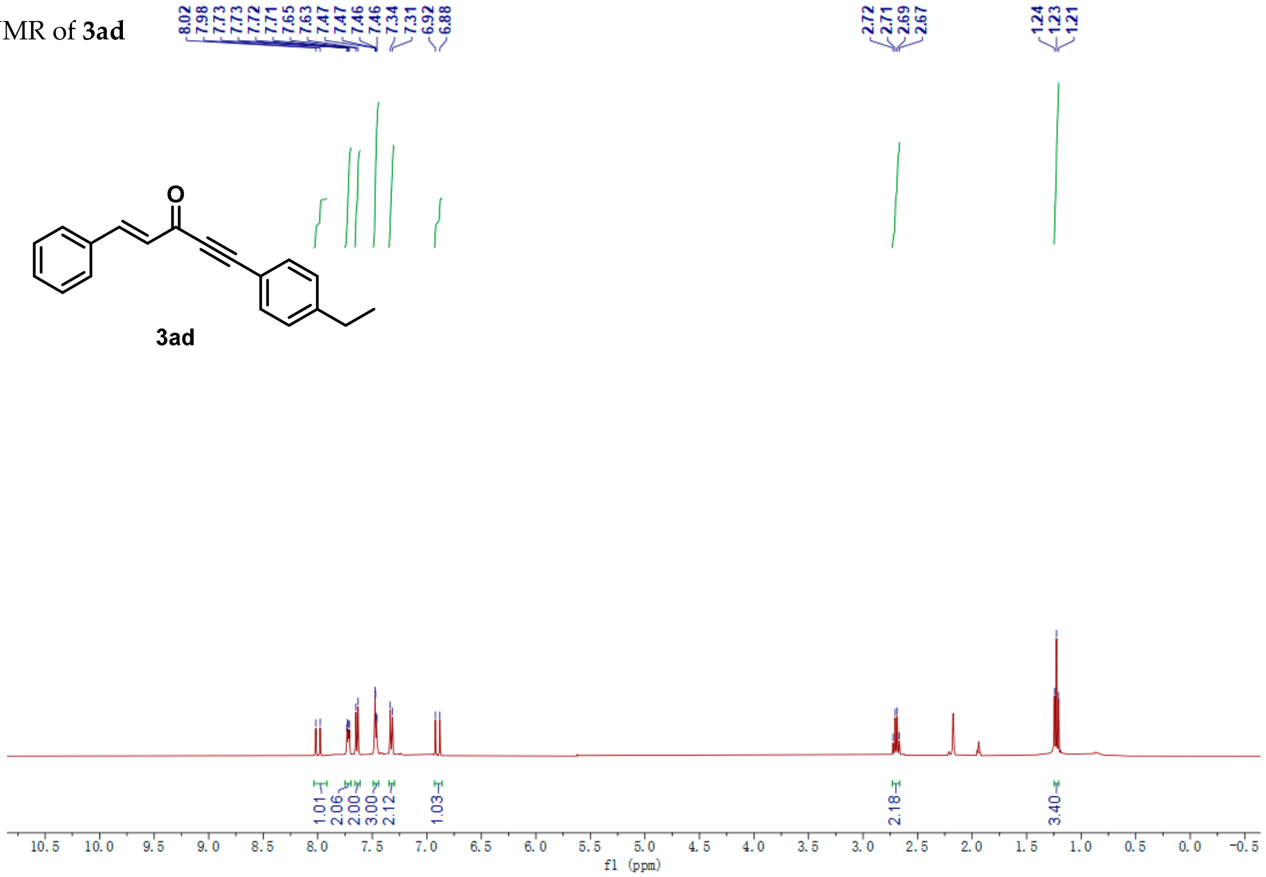

^{13}C NMR of **3ad**

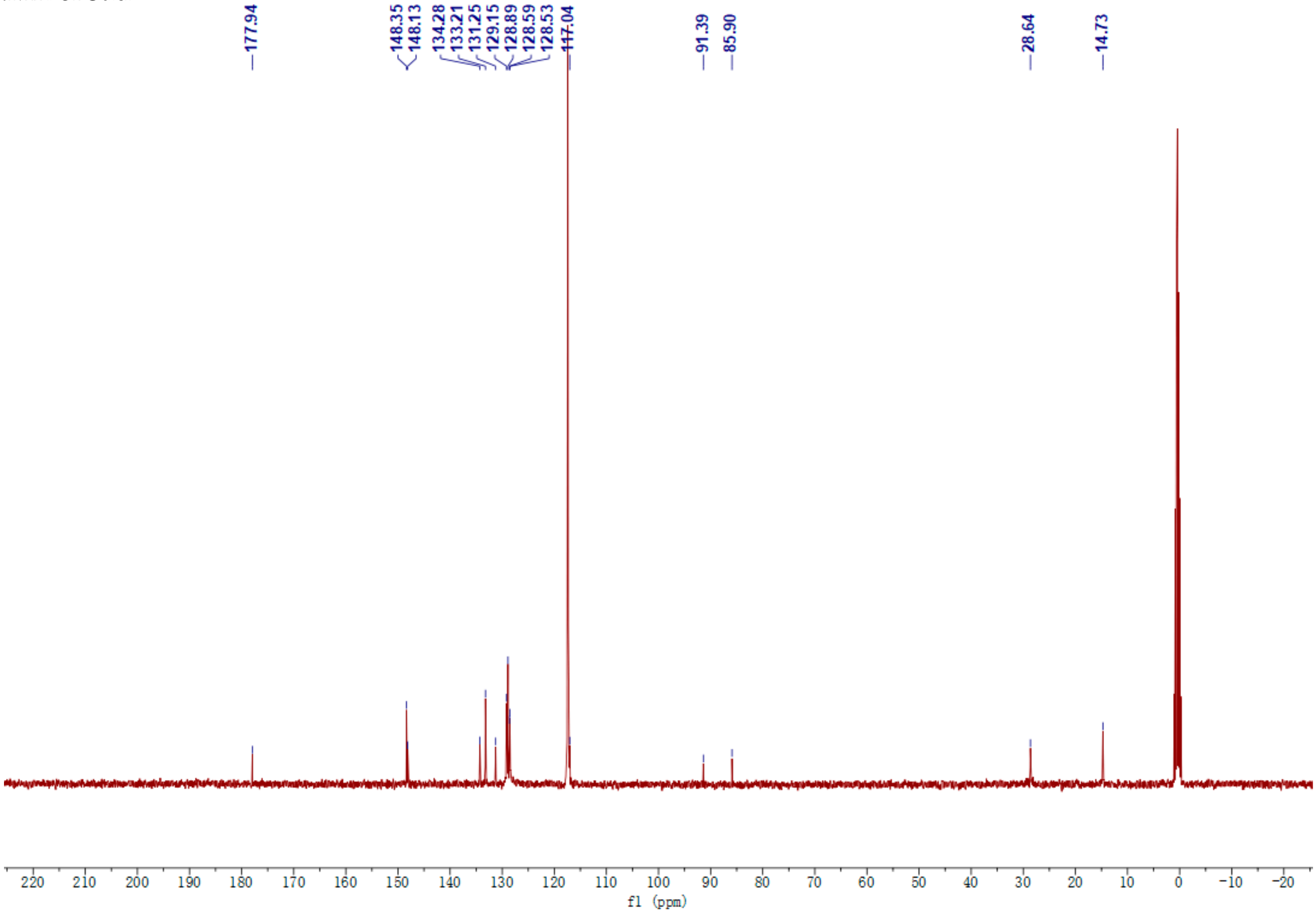

IR of **3ad**

IR spectra data: **IR(KBr)**: ν 2958, 2927, 2857, 2208, 1625, 1596, 1501, 1450, 1308, 1262, 1166, 1097, 989, 878, 832, 807, 762, 674, 575 cm^{-1}.

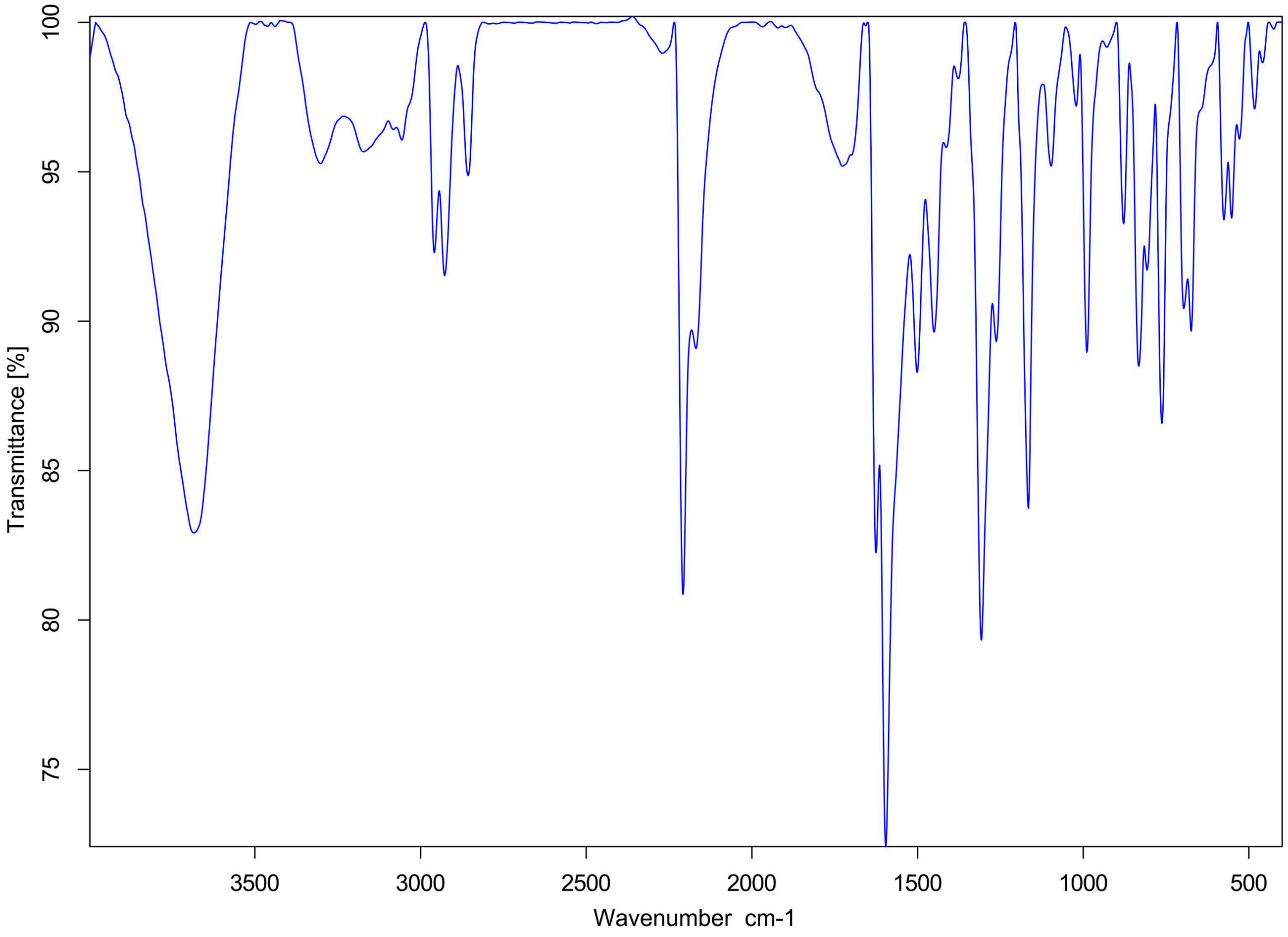

^{1}H NMR of **3ae**

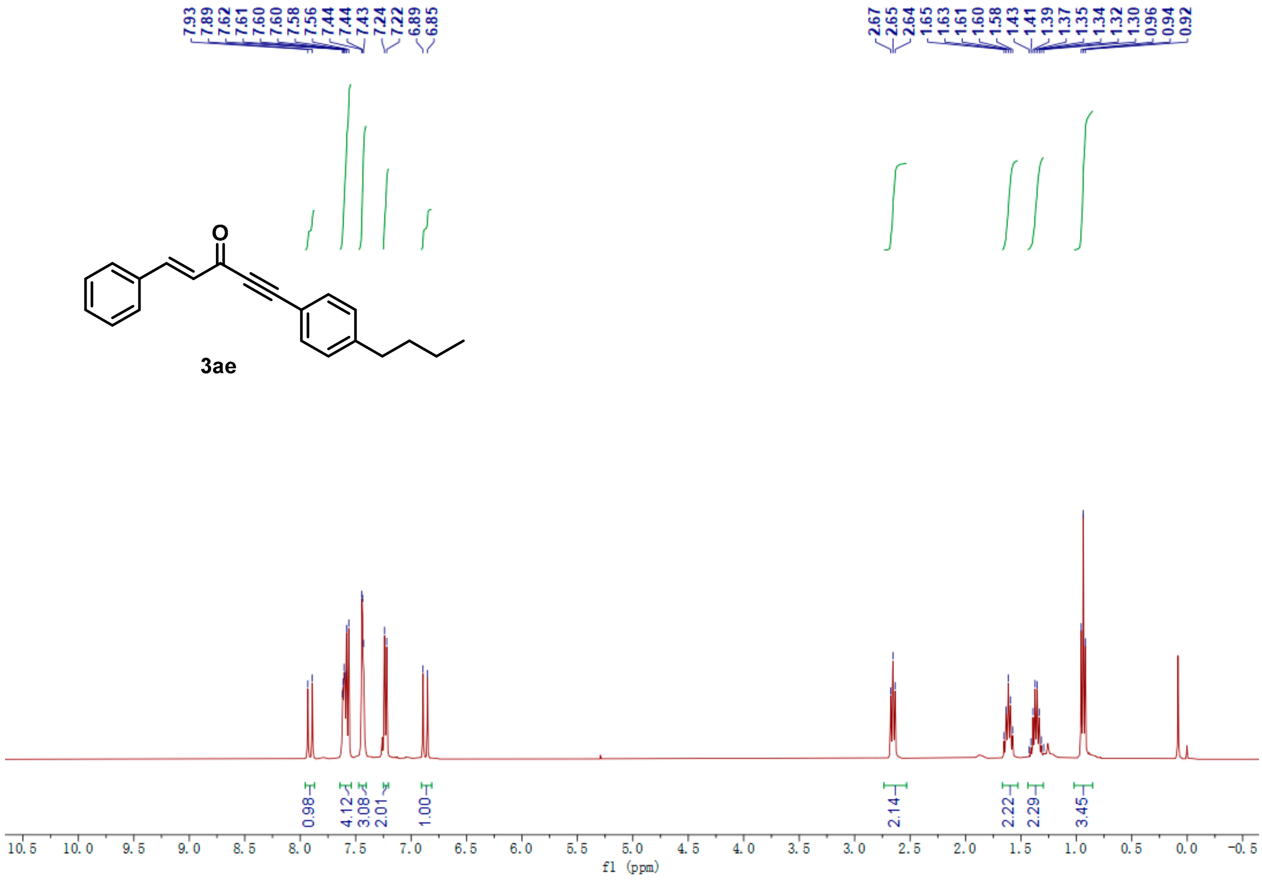

^{13}C NMR of **3ae**

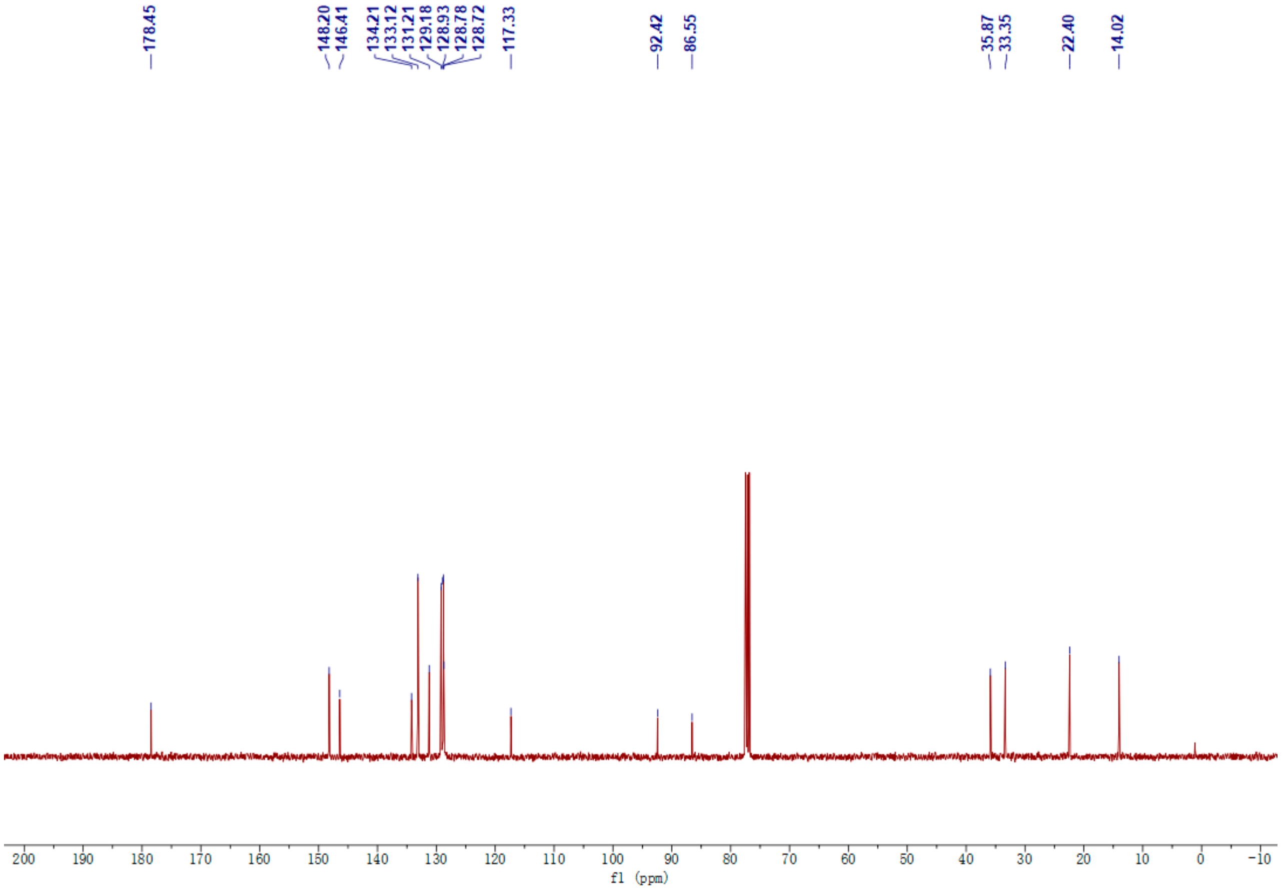

IR of **3ae**

IR spectra data: **IR(KBr)**: ν 3052, 2920, 2194, 1627, 1608, 1594, 1483, 1448, 1314, 1230, 1201, 1152, 983, 870, 781, 763, 688, 579, 543 cm^{-1}.

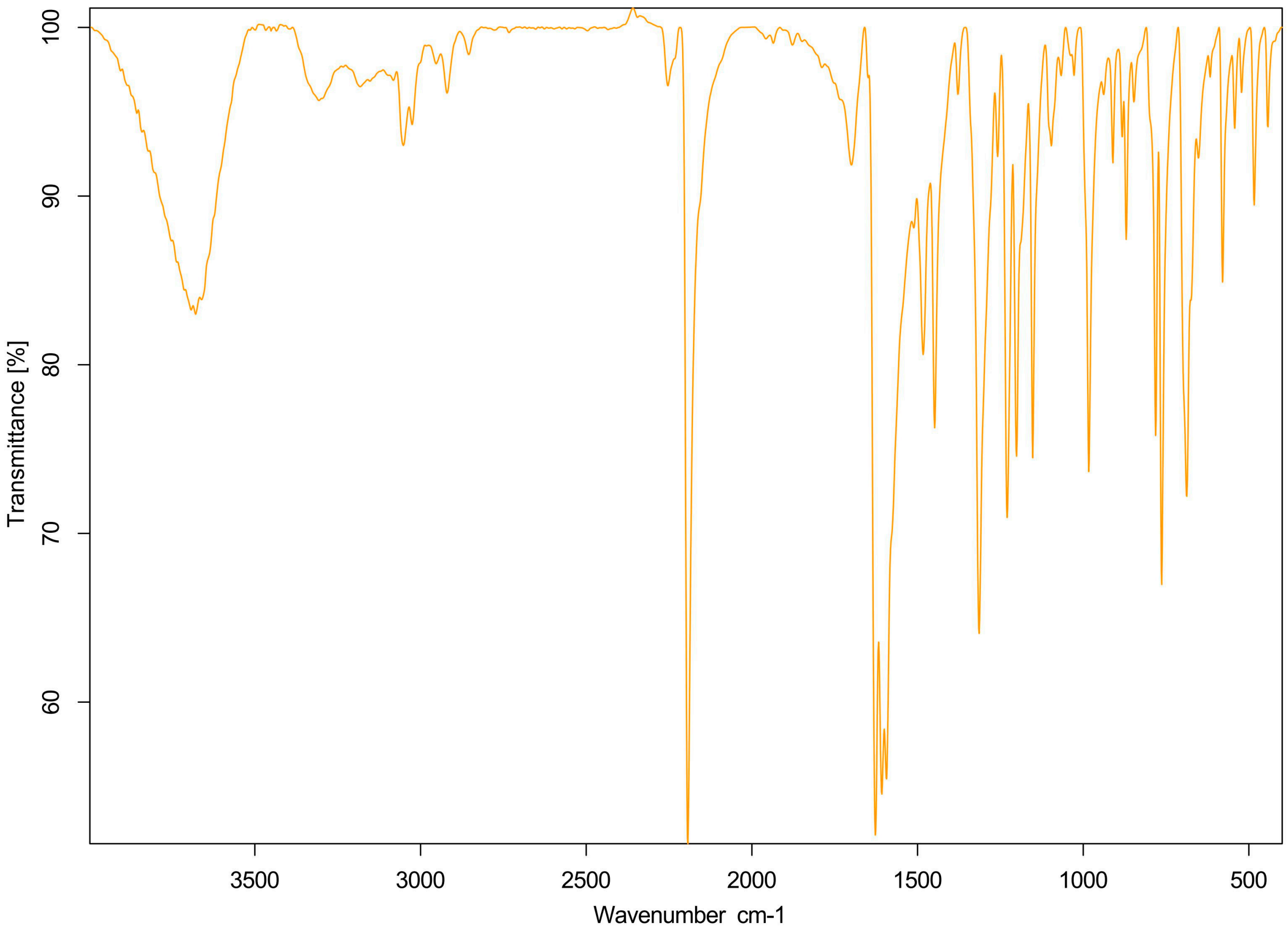

^{1}H NMR of **3af**

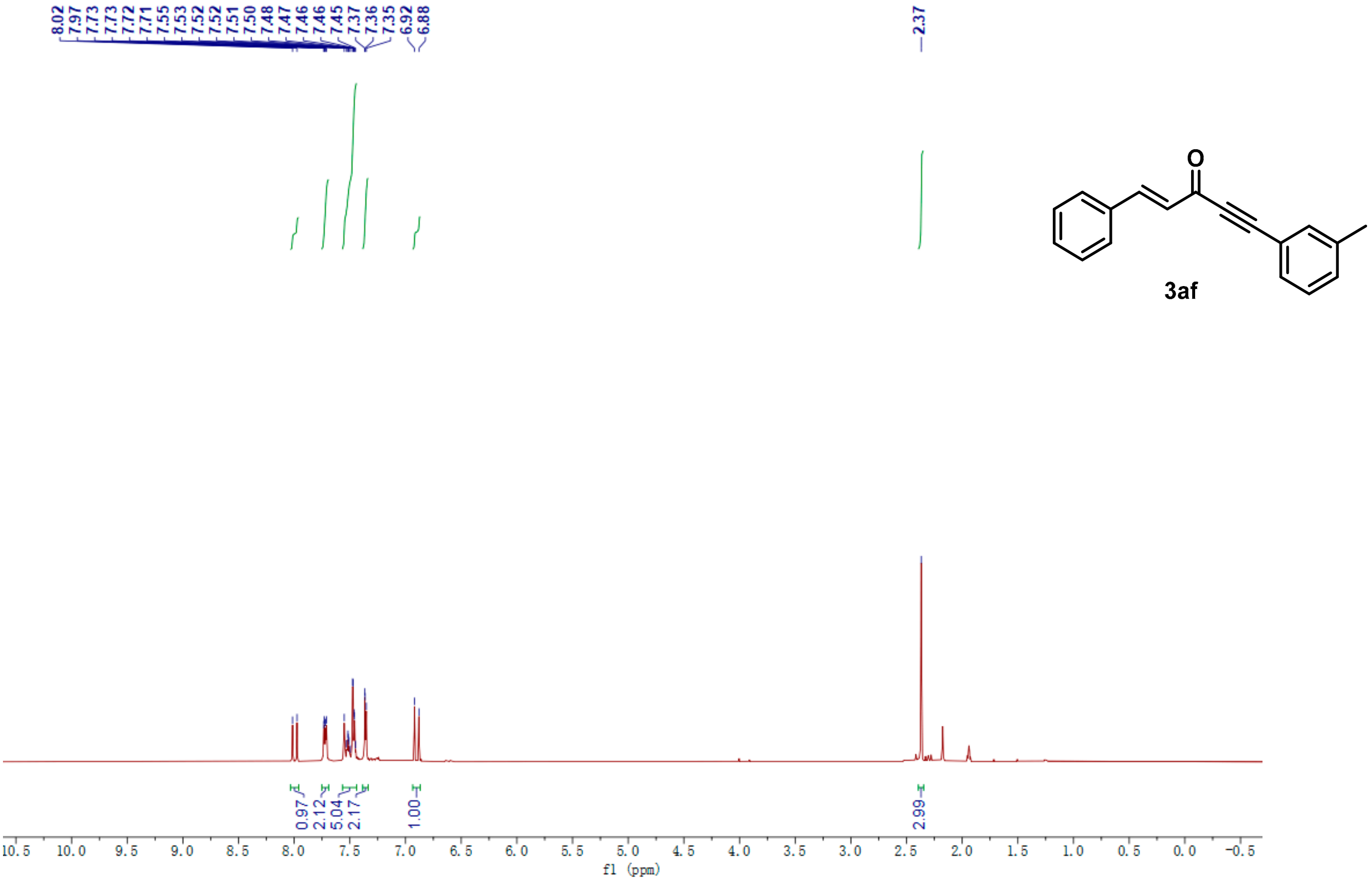

^{13}C NMR of **3af**

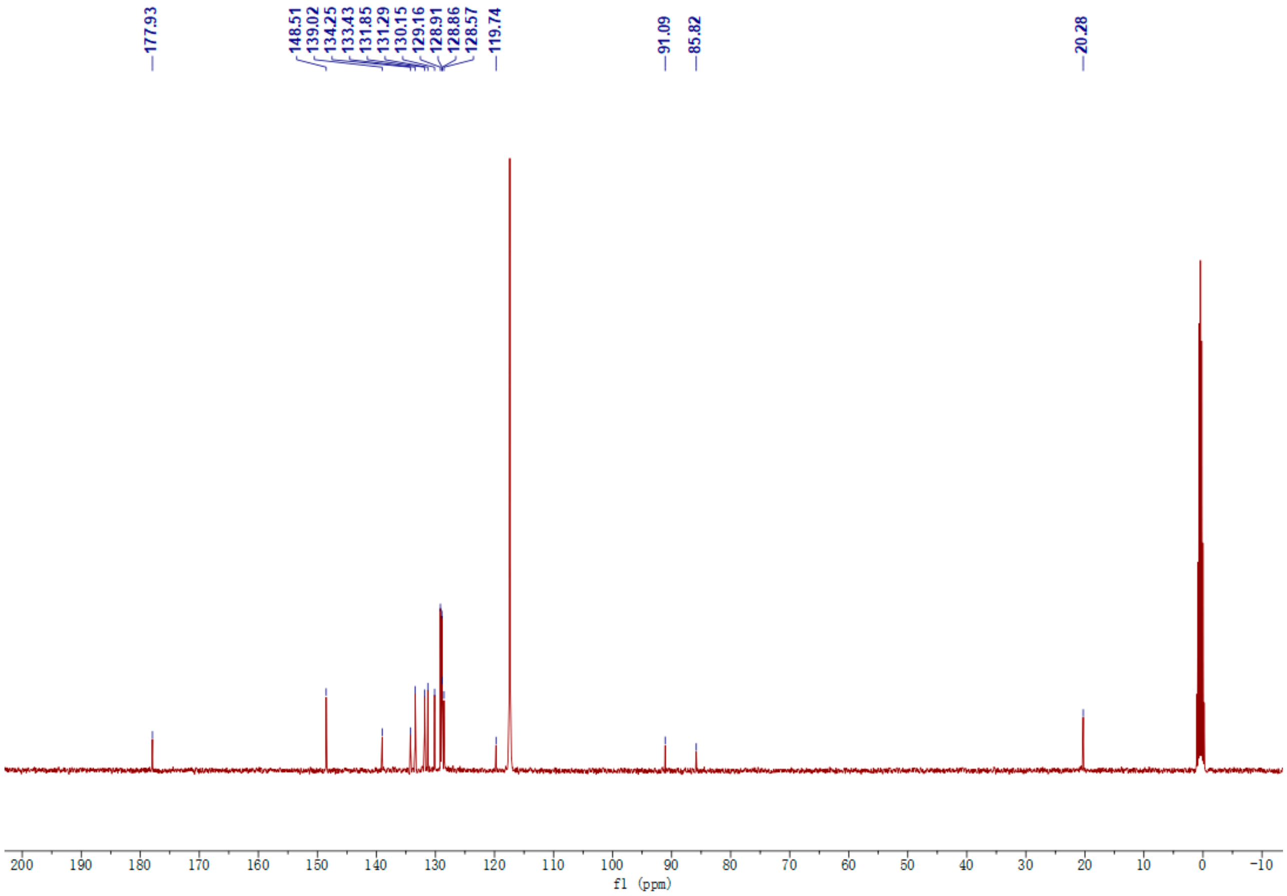

^{1}H NMR of **3ag**

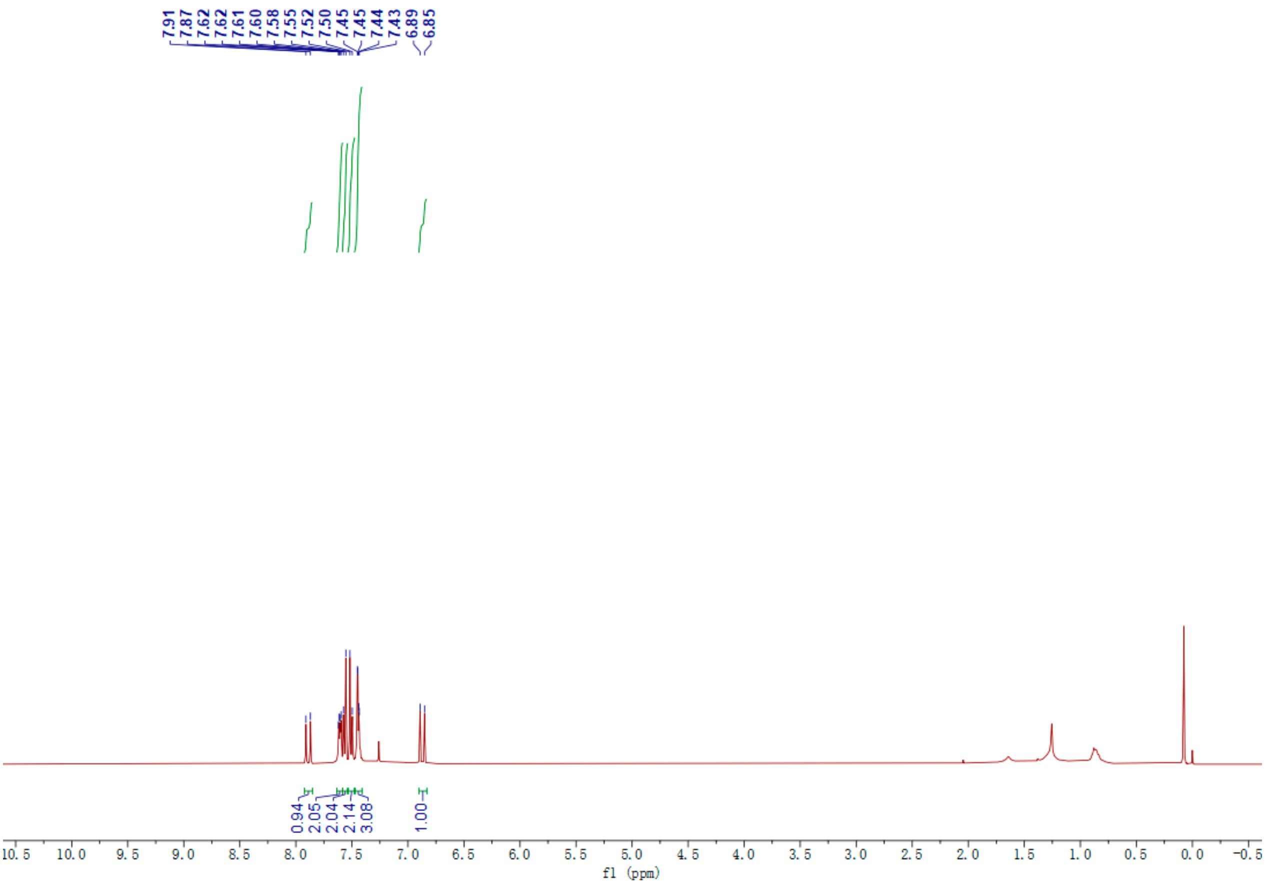

^{13}C NMR of **3ag**

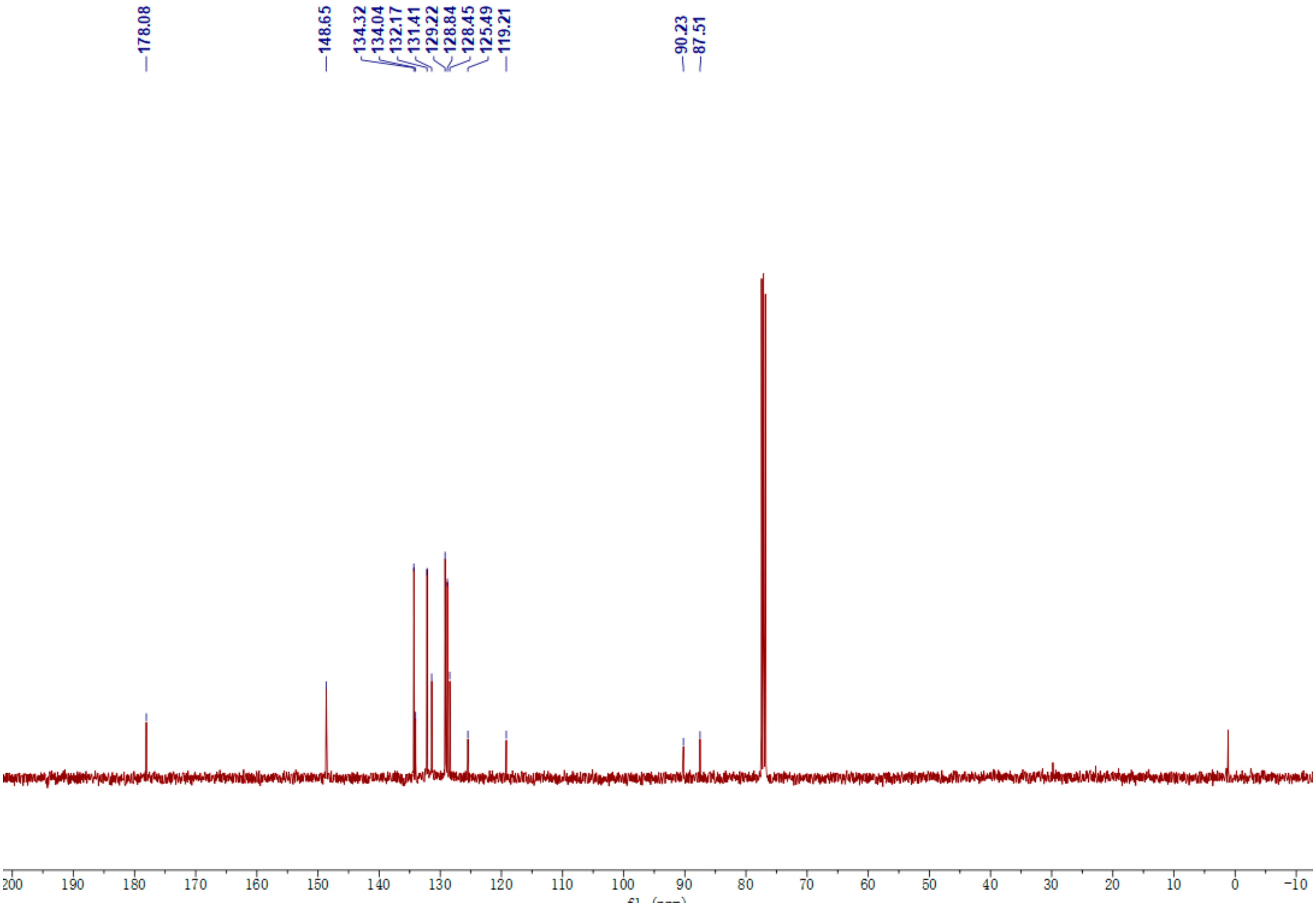

^{1}H NMR of **3ah**

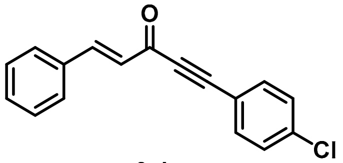

3ah

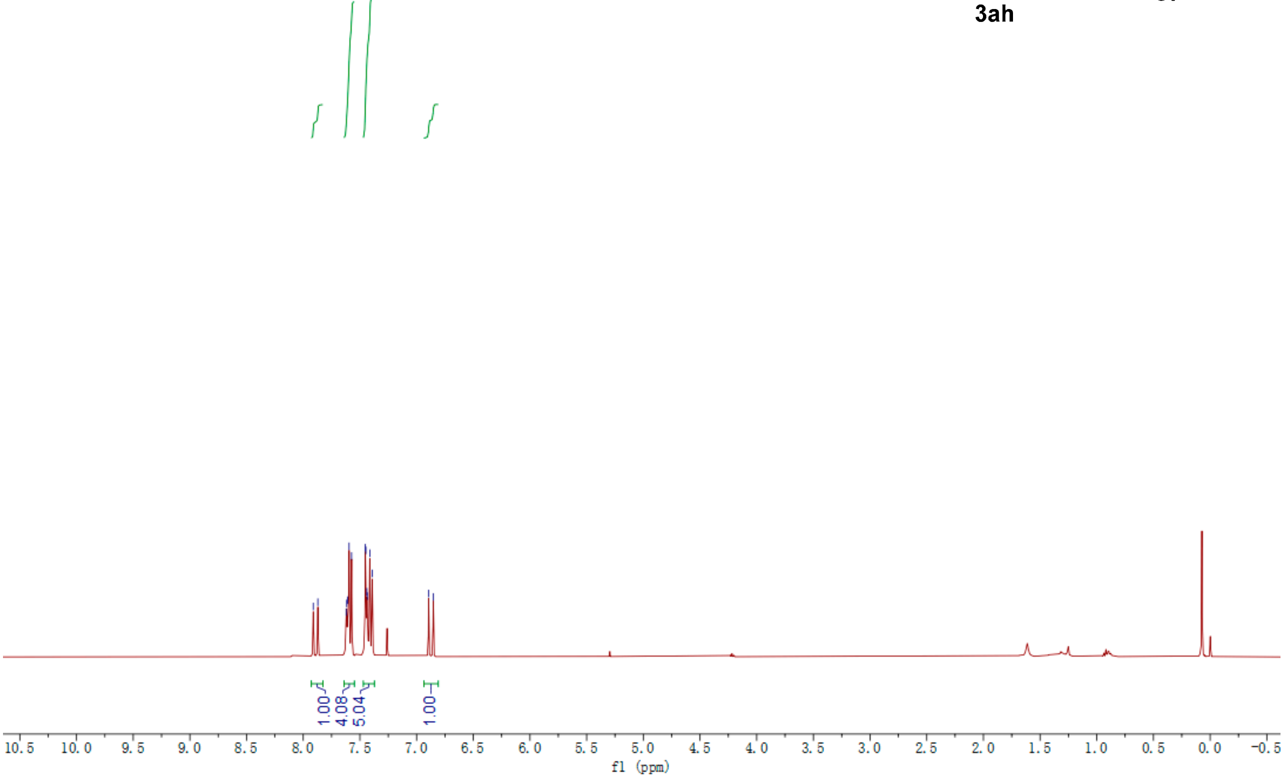

^{13}C NMR of **3ah**

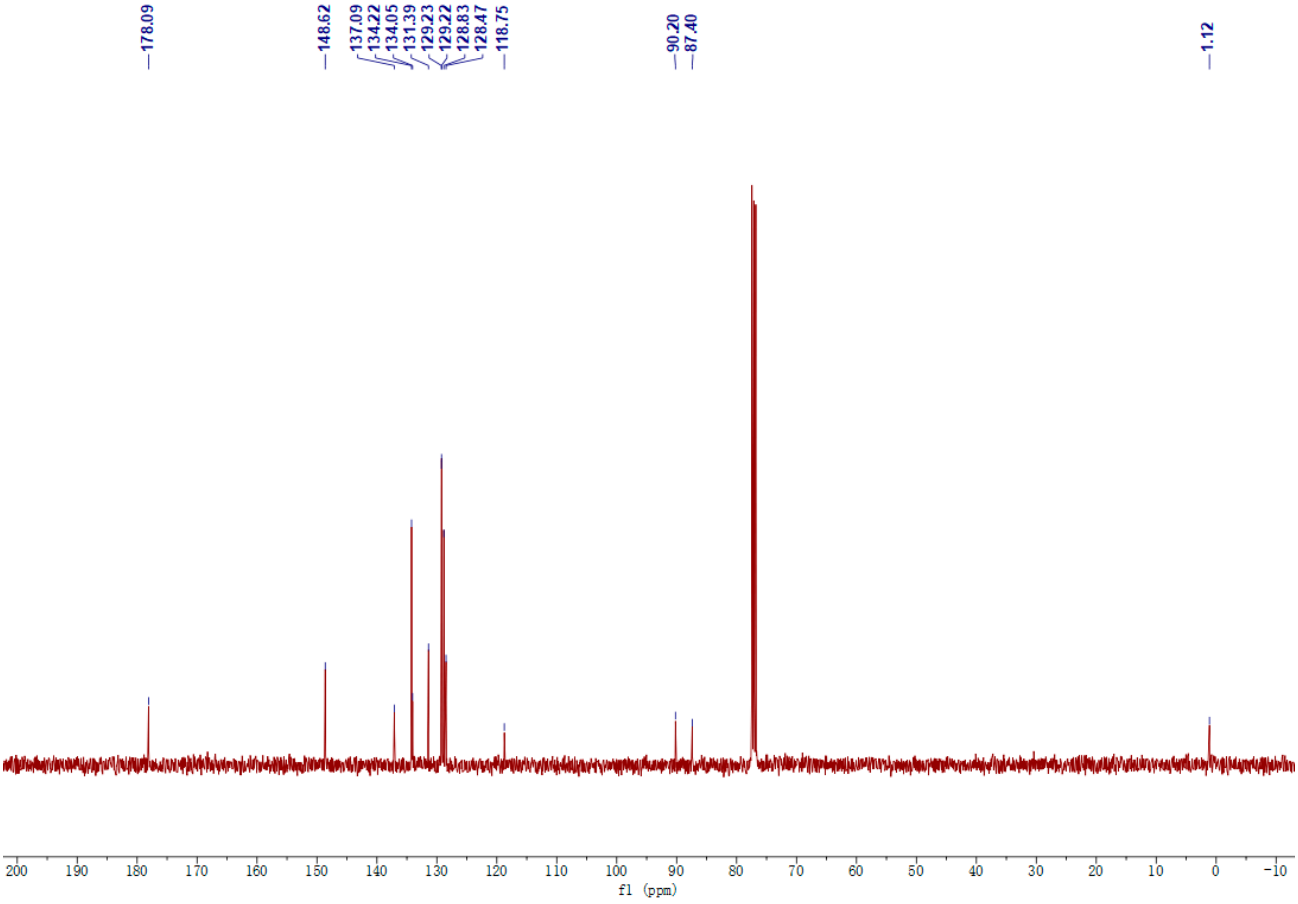

^{1}H NMR of **3ai**

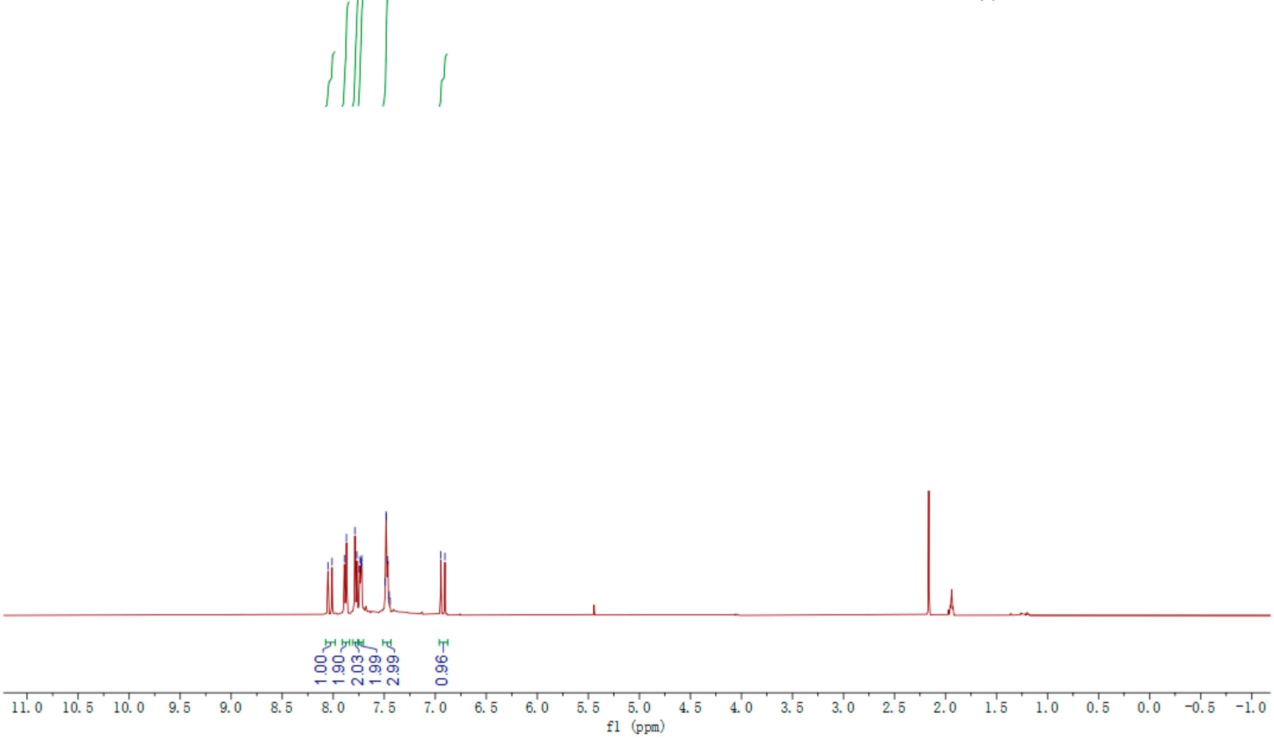

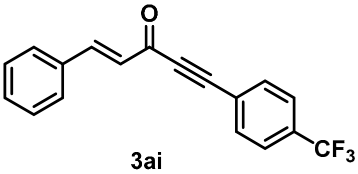

3ai

^{13}C NMR of **3ai**

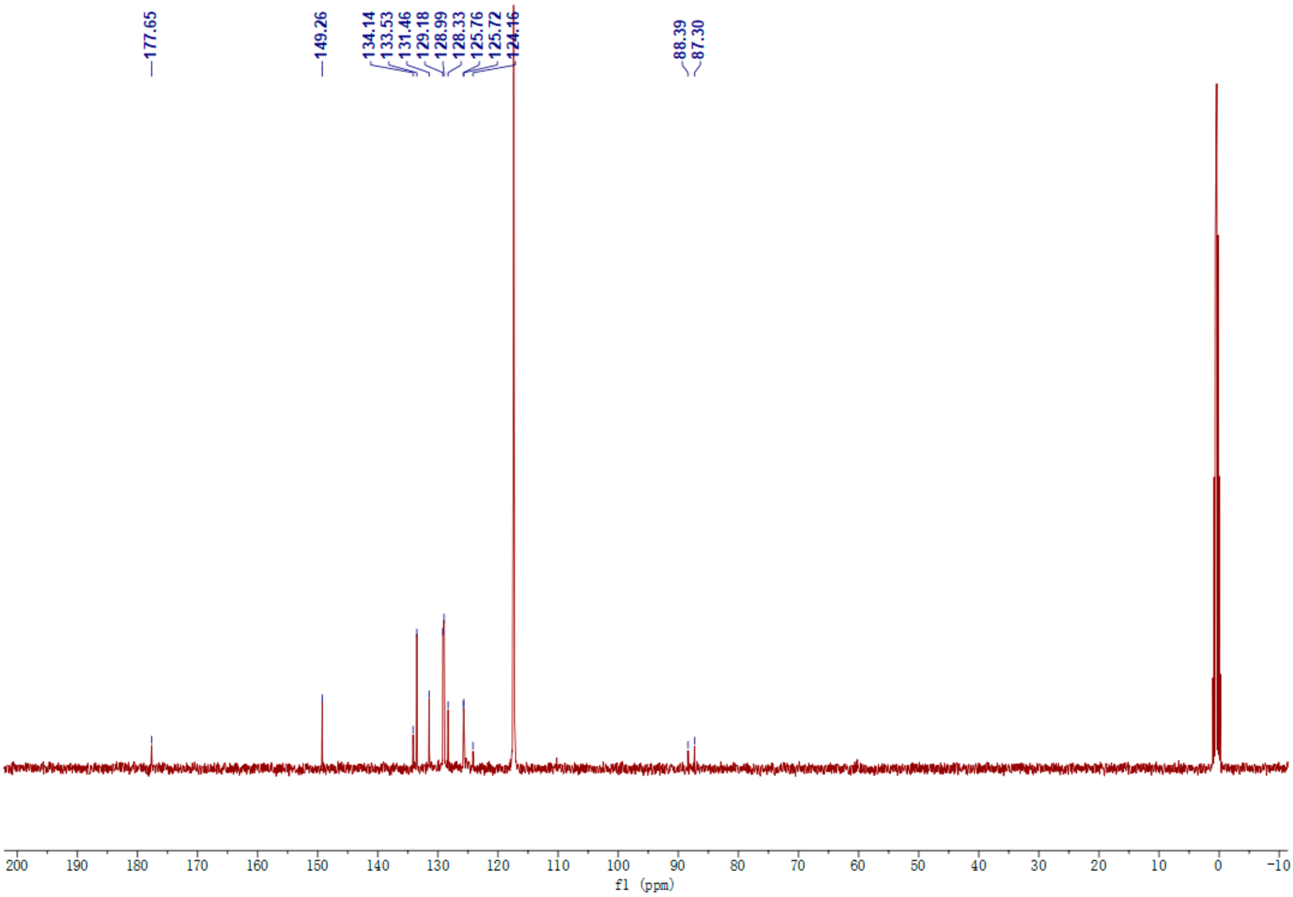

^{1}H NMR of **3aj**

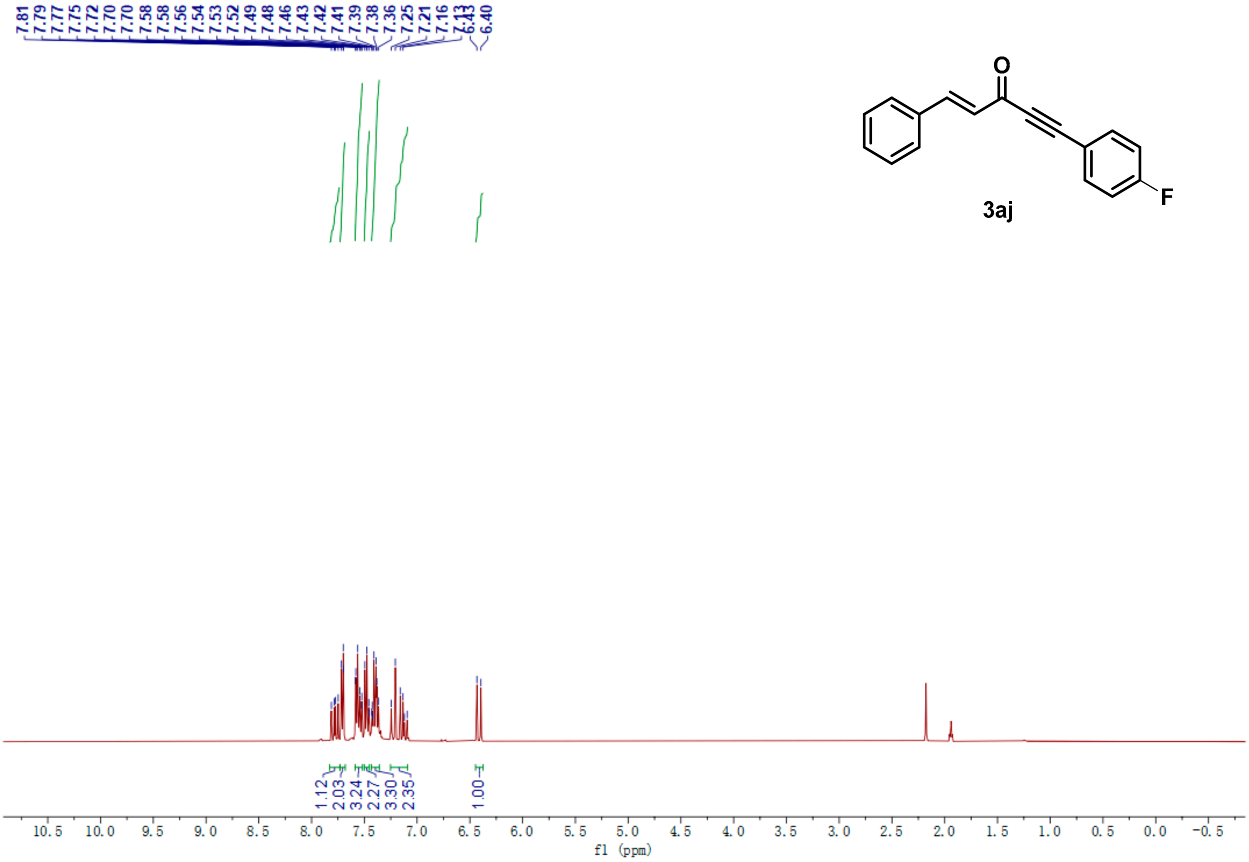

3aj

^{13}C NMR of **3aj**

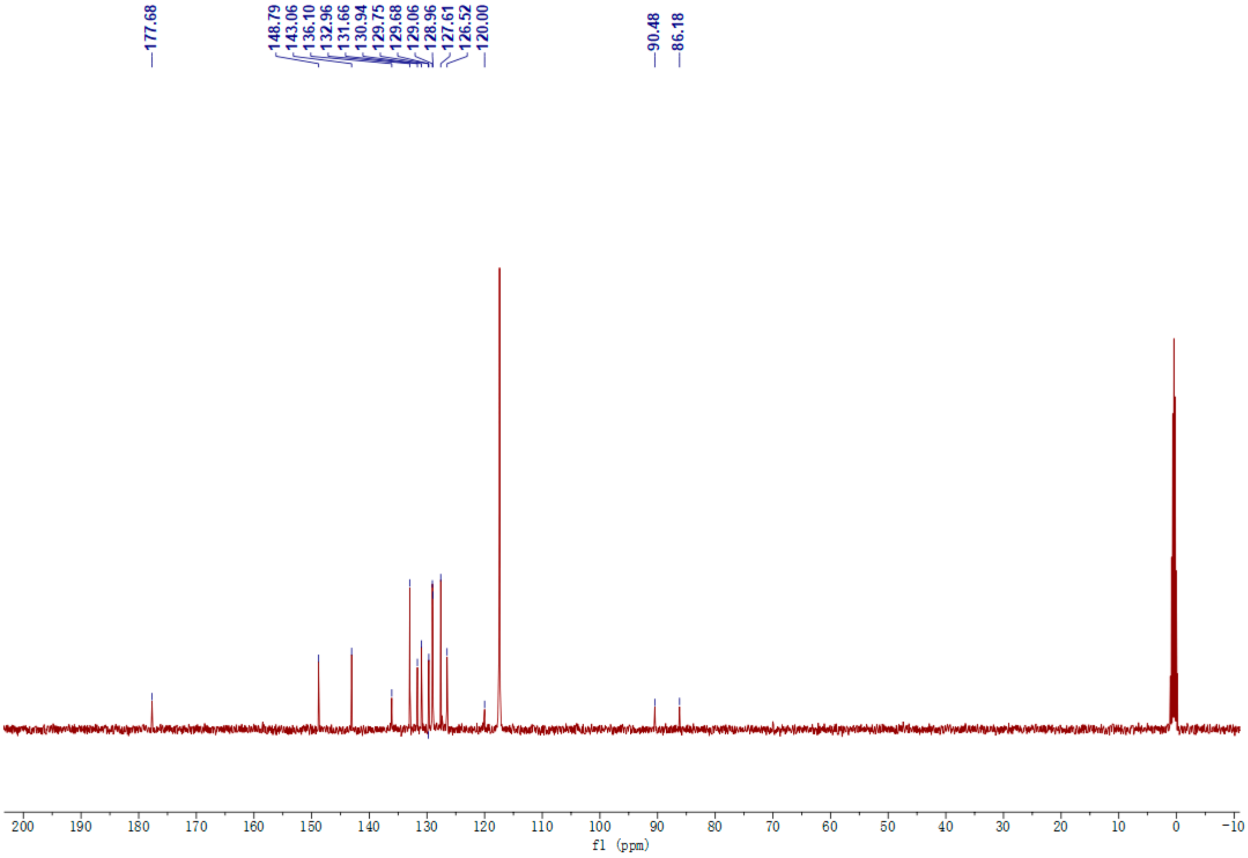

^{1}H NMR of **3ak**

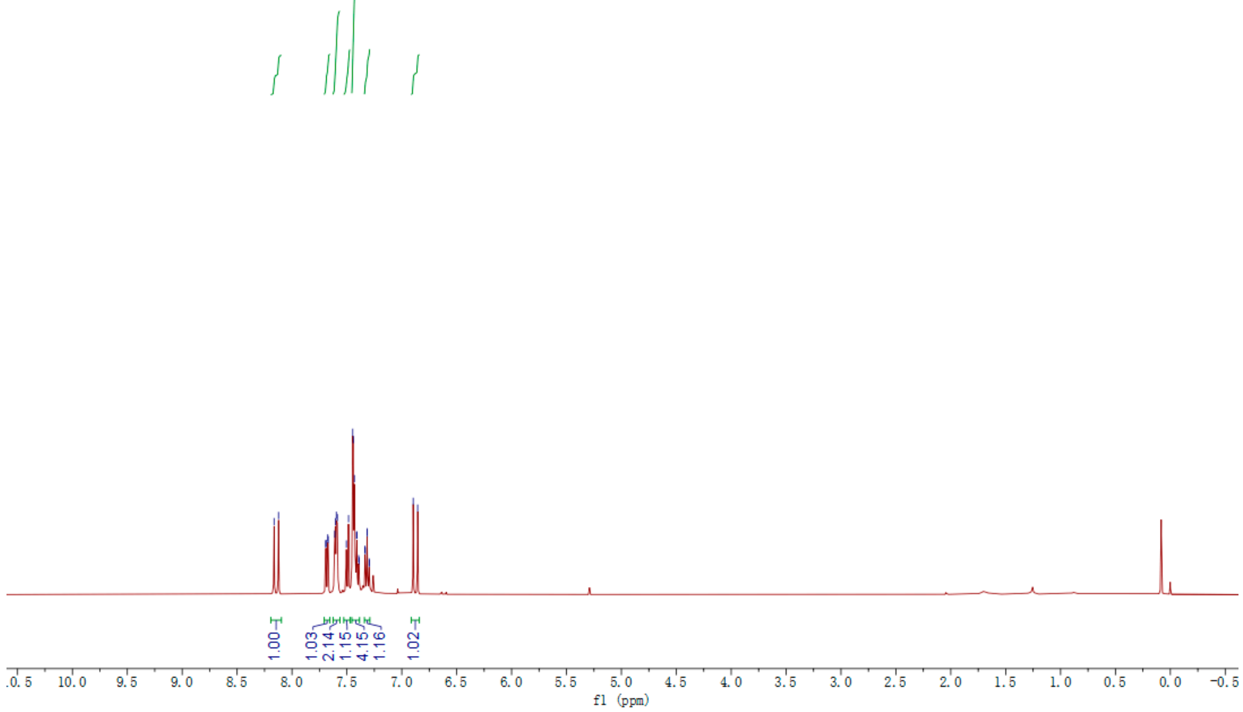

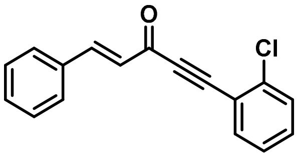

3ak

^{13}C NMR of **3ak**

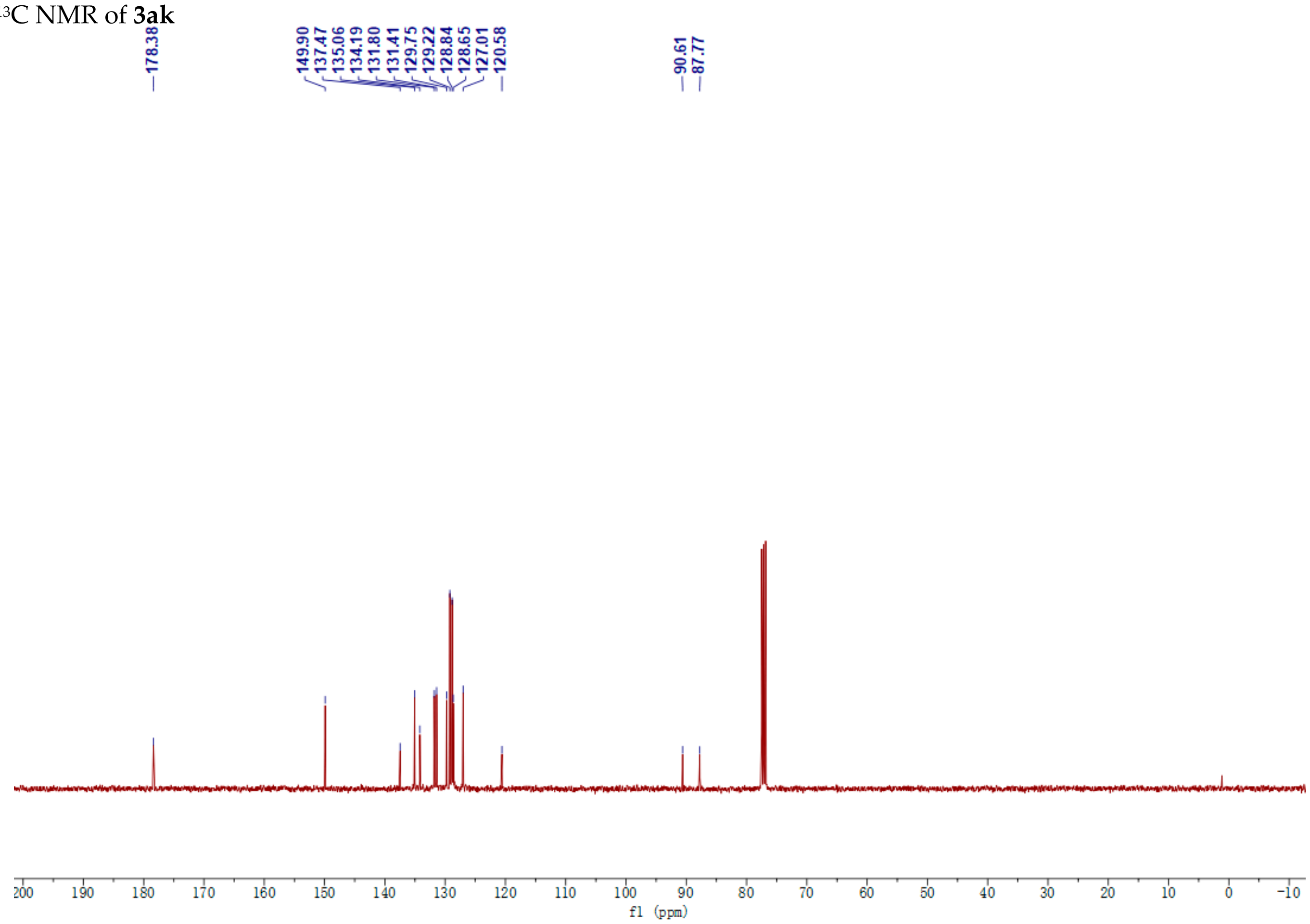

IR of **3ak**

IR spectra data: **IR(KBr)**: ν 3063, 3029, 2962, 2922, 2213, 2156, 1727, 1630, 1487, 1449, 1396, 1331, 1307, 1183, 1087, 1013, 971, 863, 863, 828, 758, 736, 691, 671, 598, 529 cm^{-1}.

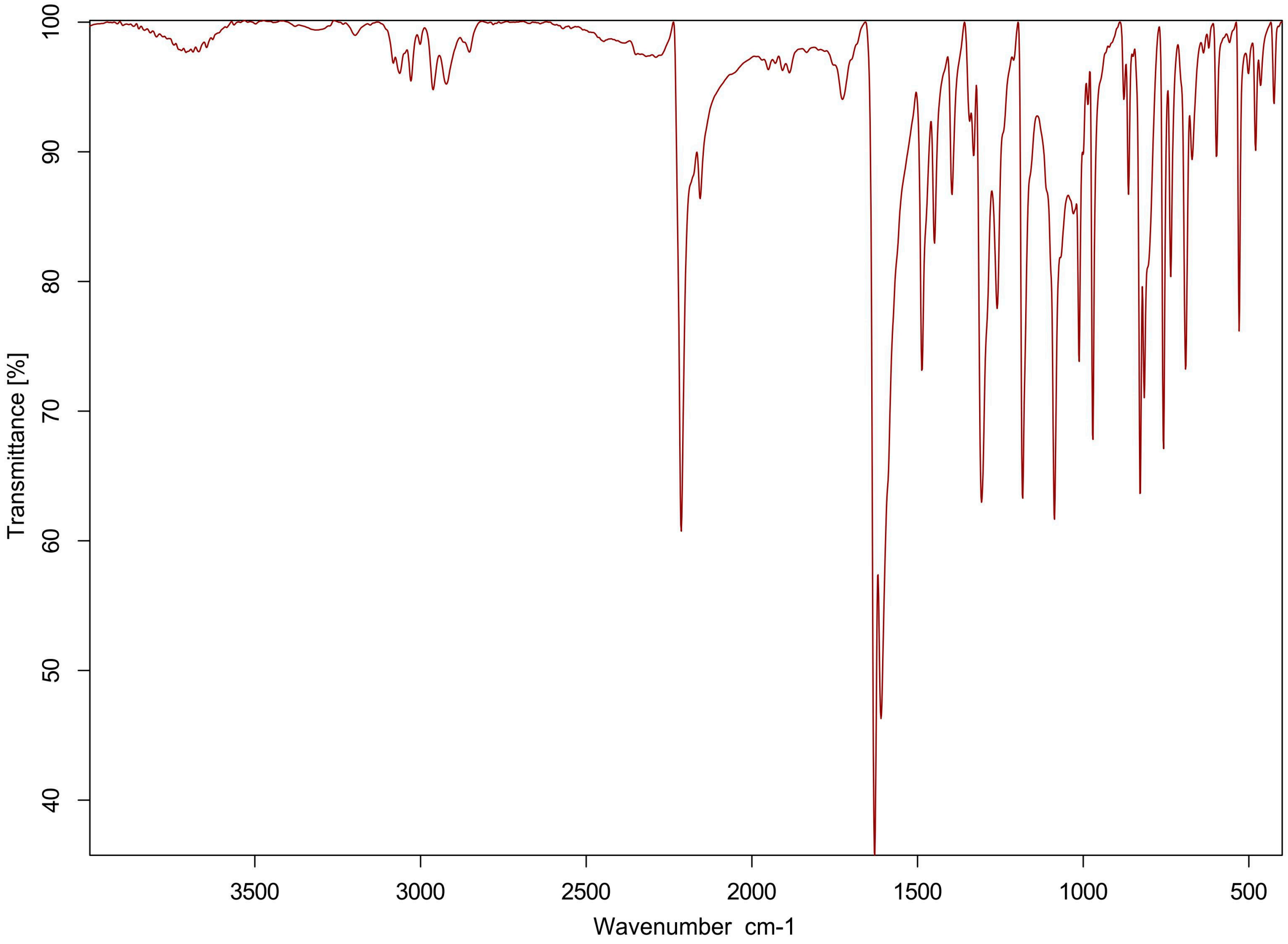

^{1}H NMR of **3al**

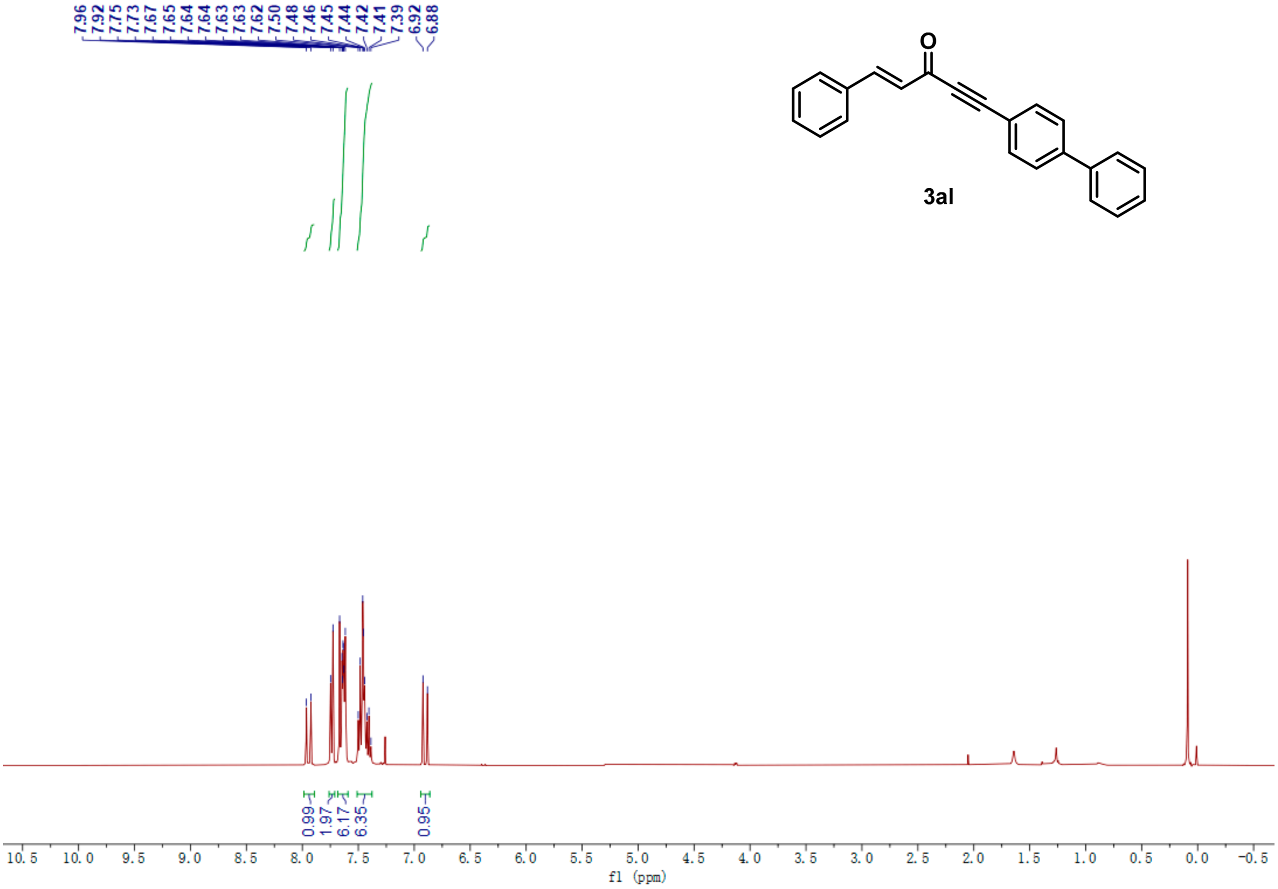

^{13}C NMR of **3al**

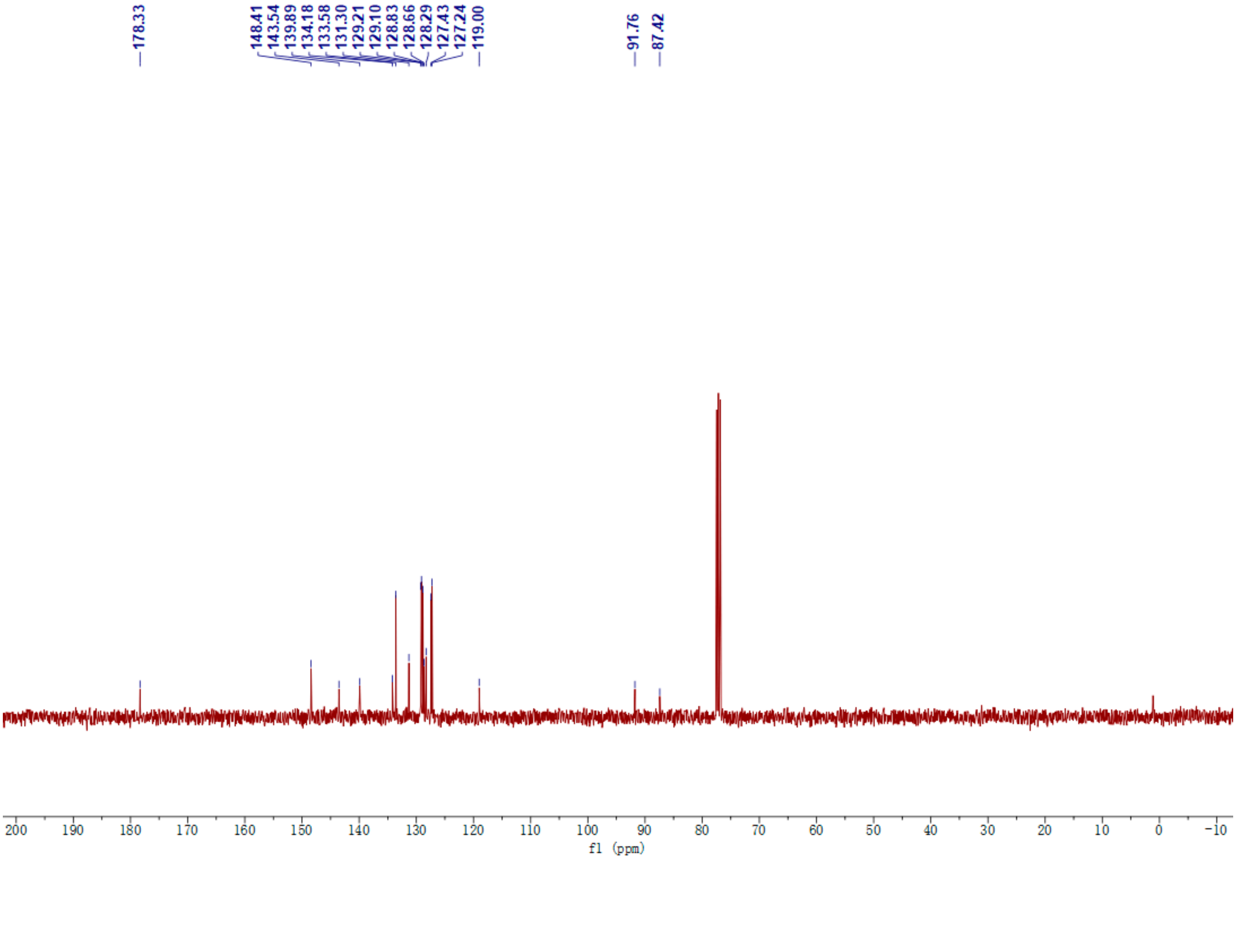

^{1}H NMR of **3am**

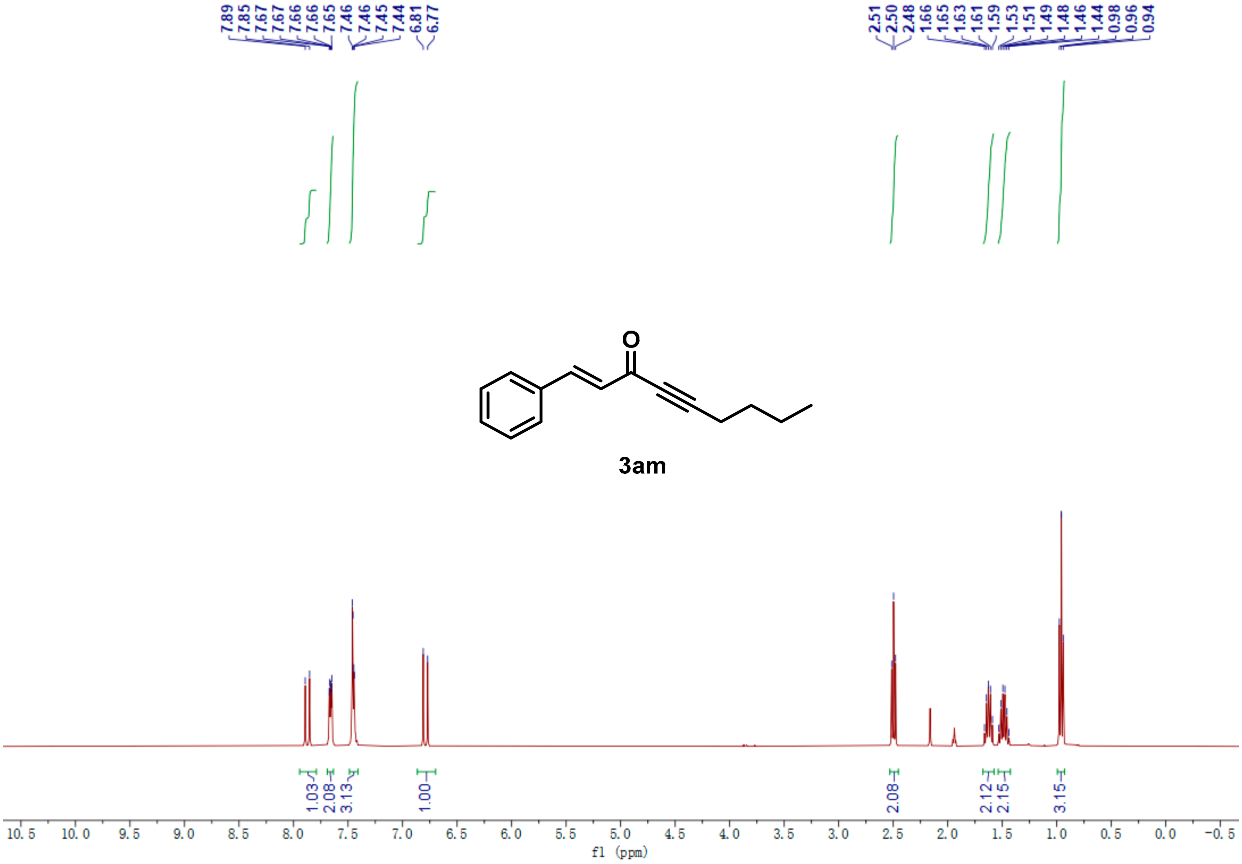

^{13}C NMR of **3am**

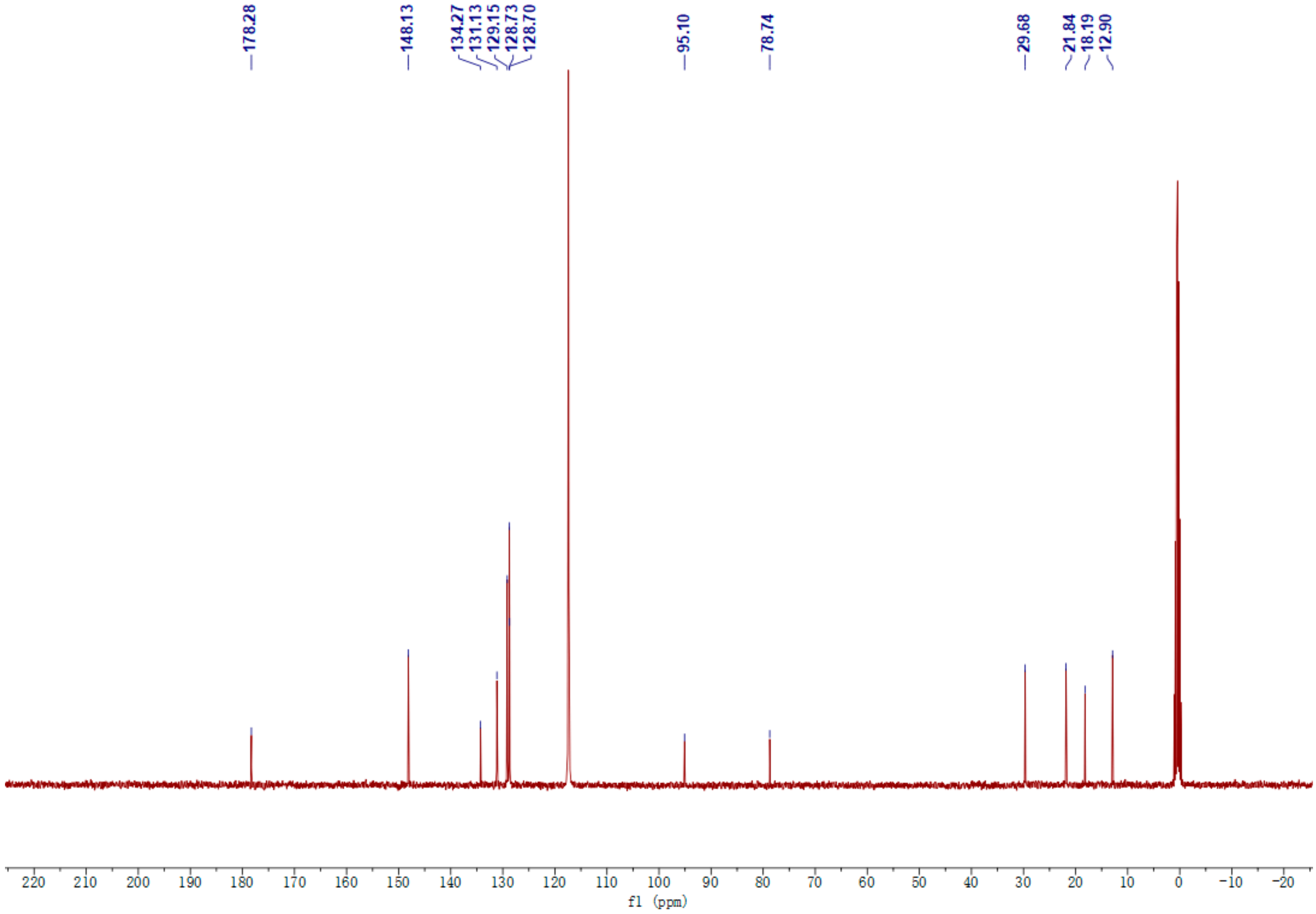

^{1}H NMR of **3an**

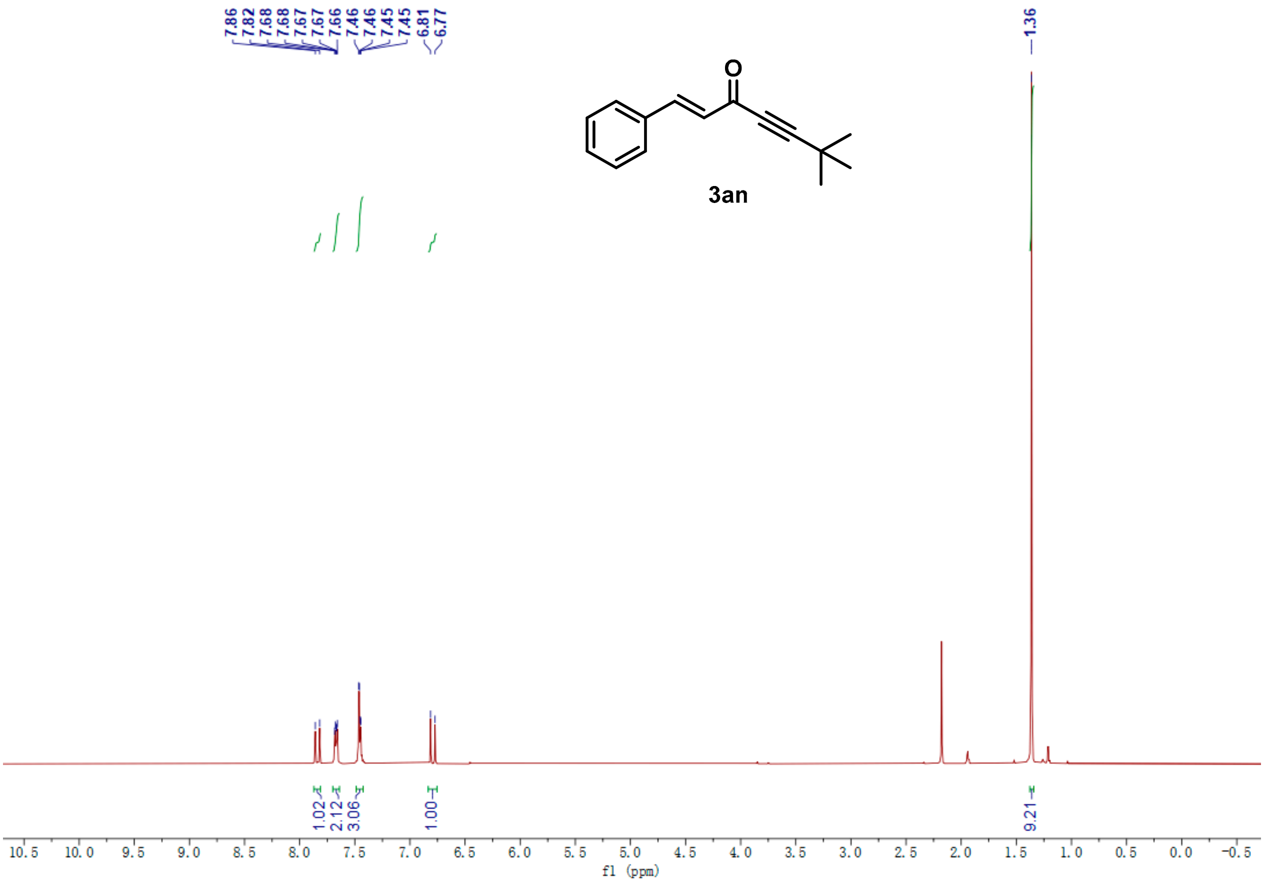

^{13}C NMR of **3an**

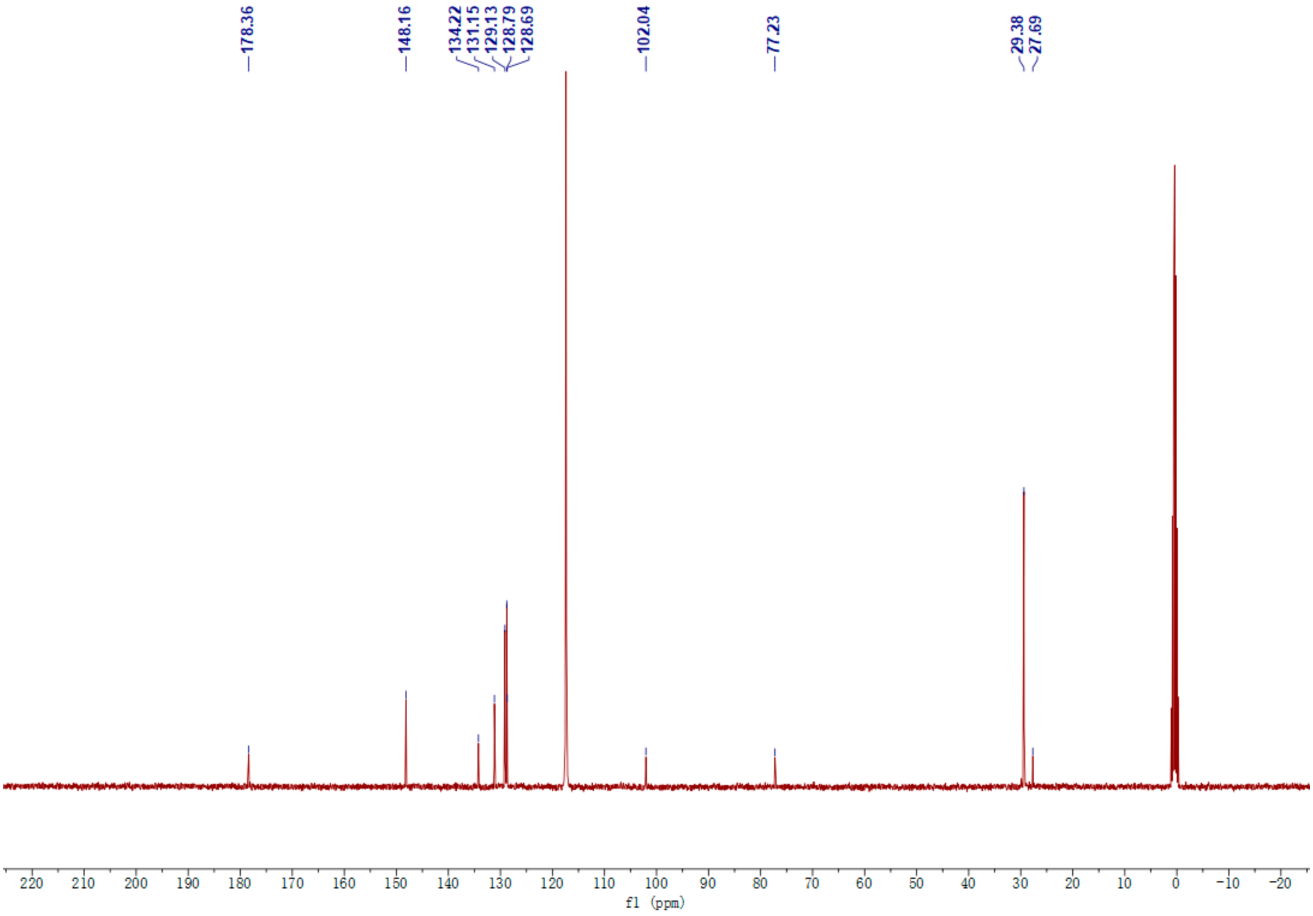

Supplement: Supplementary file 1 [file molecules-28-04364-s001.zip › molecules-2382541-supplementary.pdf]
